# Synthesis of Cyclic Oligomers of Polyether Ketone Ketone (PEKK) for Ring-Opening Polymerisation (ROP) Applications

**DOI:** 10.3390/polym16243465

**Published:** 2024-12-11

**Authors:** David Szmalko, Richard A. Evans, Jessirie Dilag, Stuart Bateman

**Affiliations:** 1 School of Engineering, RMIT University, Melbourne, VIC 3000, Australia; s3517506@student.rmit.edu.au (D.S.); jessirie.dilag2@rmit.edu.au (J.D.); 2 CSIRO Manufacturing, Clayton, VIC 3168, Australia; richard.evans@csiro.au

**Keywords:** PEKK, thermoplastic, ED-ROP, macrocyclic oligomers, *in situ* polymerisation

## Abstract

Entropy-Driven Ring-Opening Polymerisation represents an attractive mechanism to produce high-performance polymeric materials as it can be performed using neat, low-viscosity precursors and without the production of by-products or release of volatiles. Macrocyclic oligomers (MCOs) of polyether ketone ketone (PEKK) were synthesised and investigated as an *in situ* method of forming this high-performance thermoplastic. Cyclic oligomers were successfully synthesised by pseudo-high dilution methods, and the reaction conditions were optimised through careful addition of starting materials and carbonate base selection. These novel compounds were characterised, X-ray crystal structures were obtained, and the synthesis method was extended from the homopolymers to MCOs with the structural isomers predominantly used in industry. PEKK formed from MCOs were characterised by DSC, TGA and GPC and found to have similar glass transitions and molecular weight averages to those of a commercial PEKK polymer.

## 1. Introduction

**Scheme 1:**
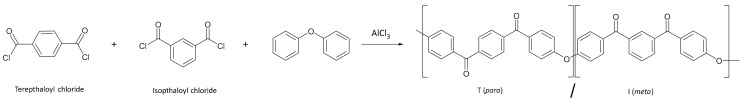
Outline of Friedel–Crafts acylation of diphenyl ether by terepthaloyl and isopthaloyl chloride, producing a PEKK polymer with a mixed amount of *para* and *meta* ketone linkages (i.e., the T/I ratio).

Thermoplastic carbon fibre composites have received considerable attention in aerospace manufacturing as an environmentally friendly alternative to thermosetting (epoxy-based) materials. This is due to their ability to be thermally processed multiple times, thereby providing numerous reuse or recycling options at the end-of-life [1]. Polyaryl ether ketones (PAEKs) are a group of high-performance thermoplastic polymers defined by their structure of repeat ether and ketone linkages between aromatic rings. Such polymers find use as composite matrices in the aerospace industry, due to their high thermal stability, competitive mechanical properties and chemical resistance [2]. From amongst this polymer family, polyether ketone ketone (PEKK) takes prominence as an ultra-high-performance thermoplastic, with high operating temperatures [3], compression resistance and superior thermal resistance [4]. PEKK is primarily produced commercially by employing Friedel–Crafts acylation [5], which is fast and high yielding. Utilising different ratios of terephthaloyl chloride (T) and isophthaloyl chloride (I), which form *para* and *meta* links in the polymer backbone (illustrated in Scheme 1), provides the opportunity to fine-tune the resultant polymer’s physical and thermal properties [6], including crystallinity and rate of crystallization [7]. Whilst PEKK’s thermal stability extends its suitability to a range of high-performance applications, it also complicates the process of manufacturing, due to the high processing temperatures required (>300 °C) and high melt viscosity compared with many thermosetting resins (such as epoxy resins). These limitations impact the production of PEKK carbon fibre composite parts through reduced drapability of the carbon fibre fabric following pre-impregnation and alignment of the carbon-fibre reinforcement following moulding due to the high melt viscosity.

Such challenges could be addressed via the *in situ* polymerisation of lower-viscosity PEKK precursors via an Entropy-Driven Ring-Opening Polymerisation (ED-ROP). ED-ROP concerns the polymerisation of large, strainless ring molecules with 14 or more ring atoms, which are known as macrocyclic oligomers (MCOs). This polymerisation strategy is enabled by the linear polymer having a higher conformational entropy than the MCO at high concentrations. As such, ED-ROP can be performed neat, produces no by-products or volatiles, generates little to no exotherm and utilises MCOs that are typically much lower viscosity than their associated linear polymers: all advantages which are suited to *in situ* polymerisation [8]. In comparison, an *in situ* polymerisation with the current commercial methods for producing PEKK is infeasible due to the necessity to remove toxic by-products and the use of high boiling point solvents such as diphenyl sulfone. ED-ROP has been shown to be suitable for many different types of polymers, including polyesters, polyamides, polyacetals and PAEKs [8]. Therefore, an *in situ* ED-ROP that produces PEKK could provide a desirable new method of producing PEKK composites, with low viscosity MCOs wetting-out the reinforcing fibre without distortion and polymerisation within the mould achievable without the production of by-products or voids from the release of volatiles. Such a method would first require the synthesis of PEKK MCOs, however.

PAEK cyclic oligomers reported previously in the literature utilised high dilution syntheses to preference the formation of cyclics over linear polymers [8,9,10]. Frequently, these reports involve ether ketone or ether ketone ketone linkages interspaced with other functional groups which disrupt the intermolecular interactions and crystallinity of the polymer product and increase its solubility [11], facilitating easier isolation and analysis. However, the structure of these cyclics would also result in a polymer of lower performance compared with commercial PEKK [1]. In contrast, the MCOs of polyether ketone (PEK) reported by Ben-Haida et al. [12] and polyether ether ketone (PEEK) reported by Misasi et al. [13] provide a new mechanism to produce polyaryl ether ketones with similarity to polymers available commercially. Both groups employed high temperature, pseudo-high dilution nucleophilic substitution within a dipolar aprotic solvent to form MCOs of PEK and an *ortho* isomer of PEEK. A distribution of different cyclic oligomer sizes was revealed by both research groups with analysis by MALDI-ToF mass spectrometry. This mixture of cyclic sizes may prove advantageous to polymerisation, as all sizes can be incorporated equally into the polymer chain and an amorphous mixture would be expected to have a lower melting point.

**Scheme 2:**
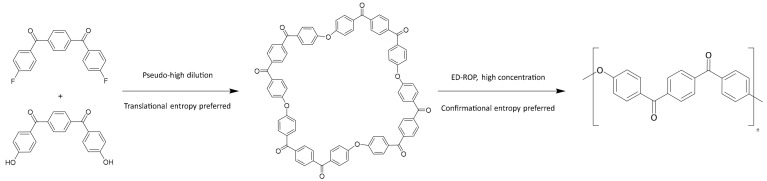
Proposed route for synthesis of PEKK MCOs (such as the example tetramer) and their polymerisation by ED-ROP.

The ED-ROP of MCOs of PAEKs has been proposed to progress via a transetherification reaction [14] and is typically initiated at high temperatures by alkali fluoride salts, such as cesium fluoride [15] or alkali phenolate salts [16], though alkali carbonates have also seen use [17]. Particularly relevant here is work performed by Colquhoun et al. [18] which polymerised a series of PAEK MCOs including a tetramer of *meta*-PEKK, the only example of a PEKK MCO reported previously. In this work polymerisation was conducted within a Differential Scanning Calorimeter (DSC) pan by placing the MCO specimen under an alumina membrane doped with CsF initiator and heating to 350–380 °C. The retrieval of polymer nanofibrils was asserted to show the flowing of the low viscosity cyclics by capillary action into the membrane. However, the full synthesis methodology of the *m-*PEKK MCO is not elucidated, and the success of this polymerisation was assessed by only the toughness and flexibility of the formed fibrils, with no other analysis of the degree of polymerisation, such as by gel permeation chromatography (GPC), reported on PEKK formed this way, to our knowledge. In order to explore the potential of PEKK produced from MCOs, this work proposes a pathway for PEKK polymerisation by ED-ROP (Scheme 2) and describes the synthesis of MCOs of PEKK by pseudo-high dilution methods for the first time, including both the *para* and *meta* homopolymers as well as MCOs with the industry-relevant T/I ratios of 60/40, 70/30 and 80/20. The MCOs are characterised by NMR, MALDI-ToF MS, GPC and IR spectroscopy, and the results of initial ED-ROP experiments are discussed with an analysis of the PEKK polymer by DSC and GPC presented and compared with an industry-standard PEKK polymer. Further investigations into the composite formation and carbon fibre interfacial interactions of PEKK MCOs are ongoing.

## 2. Materials and Methods

### 2.1. Materials and Analytical Equipment

Nuclear magnetic resonance (NMR) spectra (^1^H, ^19^F and ^13^C) were determined with a Bruker Avance 300 MHz spectrometer paired with a Bruker SampleCase automation system.

Matrix-Assisted Laser Desorption/Ionisation Time of Flight (MALDI-ToF) mass spectra were obtained with a Bruker Autoflex Speed MALDI-ToF (Bruker, Billerica, MA, USA). Analytes were prepared by dissolving the matrix, sample and cationising agent in 5% methanol in DCM at a ratio of 10/1/1 and were co-crystallised by spotting on a stainless-steel MALDI sample plate.

Differential scanning calorimetry (DSC) analyses were carried out with a TA instruments DSC 2500 (New Castle, DE, USA) within hermetically sealed aluminium pans and under a nitrogen atmosphere. Thermogravimetric analysis (TGA) studies were performed on a Netzsch STA 449 (NETZSCH Holding, Selb, Germany) high-temperature DSC/TGA within an alumina crucible under a nitrogen atmosphere. TGA-IR analyses were carried out with a Perkin Elmer STA 6000 paired with a Perkin Elmer Spectrum 100 FT-IR (Perkin Elmer, Waltham, MA, USA). The same Spectrum 100 FT-IR was used to record infrared (IR) spectra of the products on KBr discs. All solvents and reagents were purchased from Sigma Aldrich (Sigma Aldrich, St. Louis, MO, USA).

X-ray crystallography was performed on a Rigaku Synergy S diffractometer fitted with a HyPix6000 hybrid photon counting detector (Rigaku, Tokyo, Japan), using CuKα radiation (λ = 1.54184 Å). Representative colourless crystals were mounted on a nylon cryoloop and cooled to −150 °C. Non-hydrogen atoms were modelled with anisotropic displacement parameters, and hydrogen atoms attached to carbon were placed in calculated positions.

Gel permeation chromatography (GPC) was performed on a Shimadzu Prominence UPLC system equipped with an Shimadzu Prominence LC-20AT and SIL-20A Separations Module, a Shimadzu CTO-20AC column heater module, a Shimadzu RID-10A refractive index detector (Shimadzu, Kyoto, Japan) and 4× Agilent (Santa Clara, CA, USA) PL-Gel columns (3× PL-Gel Mixed C (5 μm) and 1× PL-Gel Mixed E (3 μm) columns), each 300 mm × 7.8 mm^2^, providing an effective molar mass range of 10^2^ to 4 × 10^5^. Chloroform (high-purity HPLC grade) was used as an eluent with a flow rate of 1 mL/min at 30 °C. The GPC columns were calibrated with low dispersity polystyrene (PSt) standards (Polymer Laboratories, Church Stretton, UK) ranging from 162 to 3,152,000 g/mol, and molar masses are reported as PSt equivalents. Samples were prepared by dissolving in chloroform at 2 mg/mL with agitation for short periods of time (1 to 2 h), then left static overnight. Samples were filtered through 0.45 µm disposable filters.

### 2.2. Synthetic Method

Synthesis of 1,3-phenylenebis((4-fluorophenyl)methanone) (**1**)

Isophthaloyl dichloride (6.0 g, 29.5 mmol) was dissolved in fluorobenzene (11.4 g, 118 mmol) in a round bottom flask, with anhydrous aluminium chloride (AlCl_3_) (9.85 g, 73.9 mmol) then slowly added. The reaction was then brought to reflux under an inert atmosphere for three hours.

**Scheme 3:**
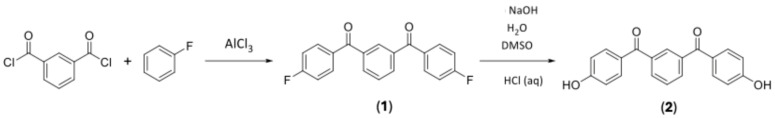
Synthesis of *m-*PEKK precursors by Friedel–Crafts acylation and subsequent hydrolysis.

The resultant thick orange mixture was chilled and quenched with 1M HCl (300 mL). The aqueous layer was extracted with DCM, with the resultant organic layer then being washed, first with 10% NaOH and then with water. The DCM was then removed by a rotary evaporator, and the resultant off-white crystals were dried under high vacuum conditions to give the final product (8.58 g, 90.2% yield). The reaction is shown in Scheme 3. ^1^H NMR spectroscopy (CDCl_3_, 300 MHz): δ = 7.21 (t, 4H, Ar-H *ortho* to -F, J = 8.55 Hz), δ = 7.68 (t, 1H, Ar-H *meta* to carbonyl, J = 7.71 Hz), δ = 7.89 (m, 4H, Ar-H *meta* to -F), δ=8.02 (dd, 2H, Ar-H *ortho* to Ar-H, *ortho* to carbonyl, J = 1.5 Hz, 7.5 Hz), δ = 8.16 (s, 1H, Ar-H *ortho* to carbonyl). ^13^C NMR spectroscopy (CDCl_3,_ 75 MHz): δ = 115.8 (d, J = 21.8 Hz), 128.7, 130.8, 132.7 (d, J = 9.2 Hz), 133.2 (d, J = 2.9 Hz), 133.4, 137.8, 165.7 (d, J = 253.2 Hz), 194.3 ^19^F NMR spectroscopy (CDCl_3,_ 282 MHz): δ = 104.8 (s, Ar-F) MP = 178 °C–181 °C.

Synthesis of 1,3-phenylenebis((4-hydroxyphenyl)methanone) (**2**)

1,3-Phenylenebis((4-fluorophenyl)methanone) (**1**) (3.0 g, 9.31 mmol) was dissolved in DMSO (30 mL) in a round bottom flask, with NaOH (1.49 g, 37.2 mmol) dissolved in deionised water (3 mL) then being added dropwise. The reaction vessel was fitted with a condenser and heated to 70 °C overnight.

The reaction mixture was poured into water (300 mL) and washed with chloroform to remove unreacted starting material. The aqueous layer was then acidified with 5.5 M HCl (60 mL), and the resultant precipitate was collected by vacuum filtration and washed with water to yield the product as a fluffy, off-white powder (2.56 g, 86.5% yield) ^1^H NMR spectroscopy (CD_3_OD, 300 MHz): δ = 6.90 (d, 4H, Ar-H *ortho* to -OH, J = 8.4 Hz), δ = 7.68 (m, 5H, Ar-H *meta* to -OH and Ar-H *meta* to carbonyl), δ = 7.84 (s, 1H), δ = 7.92 (d, 2H, Ar-H *ortho* to Ar-H, *ortho* to carbonyl, J = 7.5 Hz), δ = 10.49 (br s, 2H, -OH). ^13^C NMR spectroscopy (CD_3_OD, 75 MHz): δ = 115.1, 128.1, 128.3, 130.3, 132.5, 132.8, 138.4, 162.4, 195.5.

Synthesis of cyclic oligomers of *m-*PEKK (**3**)

**Scheme 4:**
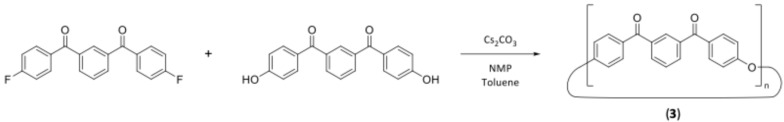
Synthesis of *m-*PEKK MCOs under pseudo high-dilution conditions.

A twin-necked round bottom flask was dried and flushed with nitrogen before being fitted with an attached fritted nitrogen inlet and Dean–Stark apparatus. To this flask, anhydrous NMP (40 mL), distilled toluene (10 mL) and cesium carbonate (0.44 g, 1.36 mmol) were added, and the system was heated to 165 °C. 1,3-phenylenebis((4-fluorophenyl)methanone) (**1**) (0.4 g, 1.24 mmol) and 1,3-phenylenebis((4-hydroxyphenyl)methanone) (**2**) (0.4 g, 1.26 mmol) were dissolved in anhydrous NMP (20 mL) and added to the vessel via a syringe pump over the course of 24 h. Nitrogen gas was bubbled continuously through the solution to aid in the removal of water. Once the addition was completed, the reaction was heated for a further hour, cooled to room temperature and poured into cold methanol (200 mL). The resultant precipitate was then collected by filtration and washed with cold water (200 mL) and methanol (100 mL). The methanol filtrate also had water (100 mL) added to form a second precipitate which was separately collected by filtration and washed with methanol (200 mL) and water (100 mL); this tan powder was later identified as pure cyclic dimer. The reaction is shown in Scheme 4. The mixed oligomer crude product was then purified by Soxhlet extraction with chloroform over 24 h, with the chloroform extract being evaporated to give the mixed cyclic oligomer product as a brown powder. (0.528 g, 71.0%) ^1^H NMR spectroscopy assigned for monomer unit (10% CF_3_COOD, 90% CD_2_Cl_2_, 300 MHz): δ = 7.31 (d, 4H, Ar-H *ortho* to -O-, J = 8.7 Hz), δ = 7.82 (m, 1H, Ar-H *meta* to carbonyl), δ = 8.01 (d, 4H, Ar-H *meta* to -O-, J = 8.7 Hz), δ = 8.17 (d, 2H, Ar-H *ortho* to Ar-H, *ortho* to carbonyl, J = 7.5 Hz), δ = 8.28 (s, 1H, Ar-H *ortho* to carbonyl). ^13^C NMR spectroscopy (CDCl_3_, 300 MHz): δ = 118.7, 130.4, 132.4, 132.6, 132.9, 133.8, 136.6, 160.2, 194.0.

Synthesis of 1,4-phenylenebis((4-fluorophenyl)methanone) (**4**)

Terephthaloyl dichloride (5.0 g, 24.6 mmol) was dissolved in fluorobenzene (9.27 g, 96.6 mmol) with anhydrous aluminium chloride (8.06 g, 60.4 mmol) then slowly added. The reaction was then brought to reflux under an inert atmosphere for three hours.

**Scheme 5:**
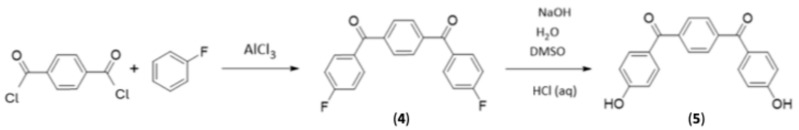
Synthesis of *p-*PEKK precursors by Friedel–Crafts acylation and subsequent hydrolysis.

The resultant thick orange mixture was then quenched with 1M HCl (300 mL). The aqueous layer was extracted with DCM, with the resultant organic layer then being washed first with 10% NaOH and then with water. The DCM was then removed by a rotary evaporator, and the resultant off-white crystals were dried under high vacuum conditions to give the final product (6.92 g, 88.7% yield). The reaction is shown in Scheme 5. ^1^H NMR spectroscopy (CDCl_3,_ 300 MHz): δ = 7.41 (dd, 4H, ArH *ortho* to -F, J = 8.55 Hz), δ = 7.88 (m, 8H, Ar-H *ortho* to carbonyl), ^13^C NMR spectroscopy (CDCl_3_, 75 MHz): δ = 115.8 (d, J = 21.8 Hz), 126.7, 132.8 (d, J = 9.2 Hz), 133.2 (d, J = 2.9 Hz), 137.8, 165.5 (d, J = 253.8 Hz), 194.2 ^19^F NMR spectroscopy (CDCl_3_, 282 MHz): δ = 104.8 (s, C-F) MP = 178 °C–181 °C.

Synthesis of 1,4-phenylenebis((4-hydroxyphenyl)methanone) (**5**)

1,4-Phenylenebis((4-fluorophenyl)methanone) (**5**) (1.8 g, 5.58 mmol) was dissolved in DMSO (20 mL), with NaOH (0.92 g, 22.4 mmol) dissolved in deionised water (2 mL) then being added dropwise. The reaction vessel was fitted with a condenser and heated to 70 °C overnight.

The reaction mixture was poured into water (200 mL) and washed with chloroform to remove unreacted starting material. The aqueous layer was then acidified with 5.5 M HCl (40 mL), and the resultant precipitate was collected by vacuum filtration and washed with water to yield the product as an off-white crystal (1.59 g, 89.5% yield) ^1^H NMR spectroscopy (CD_3_OD, 300 MHz): δ = 7.45 (d, 4H, Ar-H *ortho* to -OH, J = 8.7 Hz), δ = 7.94 (m, 8H, Ar-H *ortho* to carbonyl) ^13^C NMR spectroscopy (CD_3_OD, 75 MHz): δ = 115.8, 127.9, 129.4, 133.1, 141.0, 162.7, 194.4.

Synthesis of cyclic oligomers of *p-*PEKK (**6**)

**Scheme 6:**
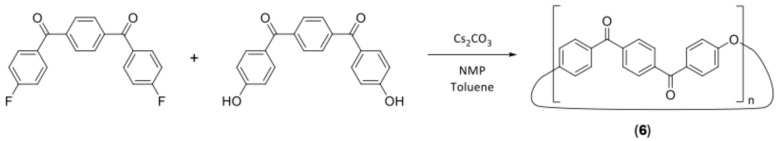
Synthesis of *p-*PEKK MCOs under pseudo high-dilution conditions.

A twin-necked round bottom flask was dried and flushed with nitrogen before being fitted with an attached fritted nitrogen inlet and Dean–Stark apparatus. To this flask, anhydrous NMP (40 mL), distilled toluene (10 mL) and cesium carbonate (0.44 g, 1.36 mmol) were added, and the system was heated to 165 °C. 1,4-Phenylenebis((4-fluorophenyl)methanone) (**4**) (0.4 g, 1.24 mmol) and 1,4-phenylenebis((4-hydroxyphenyl)methanone) (**5**) (0.4 g, 1.26 mmol) were dissolved in anhydrous NMP (25 mL) and added to the vessel via a syringe pump over the course of 24 h. Scheme 6 shows this reaction. Nitrogen was bubbled continuously through the solution to aid in the removal of water. Once the addition was completed, the reaction was heated for a further hour, cooled to room temperature and poured into cold methanol (200 mL). The resultant precipitate was then collected by filtration and washed with cold water (200 mL) and methanol (100 mL). The crude product was then purified by Soxhlet extraction with chloroform over 24 h, with the chloroform extract being evaporated to give the mixed cyclic oligomer product as a brown powder. (0.305 g, 41.0%) ^1^H NMR spectroscopy assigned for monomer unit (15% CF_3_COOD, 85% CD_2_Cl_2,_ 300 MHz): δ = 7.30 (m, 4H, Ar-H *ortho* to -O-), δ = 8.01 (m, 8H, Ar-H *ortho* to carbonyl). ^13^C NMR spectroscopy (15% CF_3_COOD, 85% CD_2_Cl_2,_ 75 MHz): δ = 119.1, 130.1, 131.7, 133.6, 140.6, 161.4, 196.7.

Polymerisation of MCOs

**Scheme 7:**
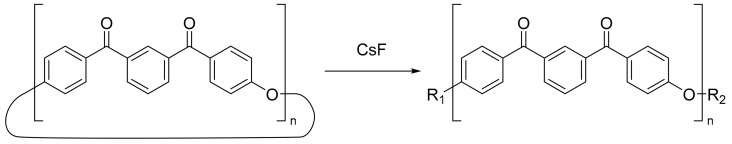
ED-ROP of *m-*PEKK MCO.

A sample of PEKK MCOs (**3** or **6**) (0.33 mmol of monomer unit, 100 mg) was dissolved in DCM with 2 mol% of CsF (1.01 mg) in methanol then added, shown in Scheme 7. The mixture was shaken and sonicated to ensure even dispersion, and the solvents were then evaporated. Samples were dried under low vacuum conditions at 120 °C overnight before being loaded into a tube furnace an evacuated to 10^−5^ mbar. They were then further dried at this higher vacuum condition at 120 °C for three hours before being heated to 350 °C for half an hour; with this higher temperature being chosen to ensure the polymers were forming in a melted state and able to participate in further chain growth. After cooling to RT, the vacuum was released, and the sample was found to be a tough, flexible, brown film that was insoluble in all common solvents including DCM.

Thioketal derivatisation of polymer

A sample of PEKK (0.067 mmol of monomer unit, 0.02 g), either formed from MCOs or commercial, was dissolved in 0.1 mL trifluoroacetic acid and 1 mL DCM under a nitrogen atmosphere. 1,3-propanedithiol (PDT) (0.20 mmol, 0.045 mL) was then added, followed by the addition of 0.025 mL of boron trifluoride diethyl etherate (BF_3_.OEt_2_) (0.54 mmol). The reaction was then stirred at room temperature for 24 h, and an intense red colour developed. Then, the reaction mixture was poured into 25 mL of cold methanol, sand the produced precipitate was collected by vacuum filtration before being washed with methanol and water. The product was then dissolved in chloroform and washed with water and brine solution, with the chloroform then being evaporated to yield the purified product as an off-white inflexible film (0.032 g, 99.4%).

## 3. Results and Discussion

### 3.1. Reaction Optimisation

While pseudo-high dilution reactions for PAEK cyclisation have appeared in the literature previously [8], these methods proved difficult to adapt and several rounds of optimisation were necessary to increase yields (Table 1). Elevated temperatures were required to complete the reaction whilst maintaining the solubility of its components, and long reaction times with the slow addition of the starting materials via a syringe pump were also needed to ensure the desired cyclics were produced. To this end, increasing the reaction temperatures and the reaction times increased yields. Due to the functional similarity between the linear and cyclic oligomers, it was expected that differentiation by NMR could prove difficult when linear oligomers were sufficiently large, and, therefore, 19F NMR was also used to show the absence of any fluorine terminated linear oligomers or unreacted starting material. Another key challenge was the removal of water formed during the reaction. Under these reaction conditions, the difluoro starting material may be partially or fully hydrolysed, either negatively affecting the ratio of starting materials or yielding a monomer which can only lead to chain extension. While the reaction was performed in an inert atmosphere and all materials that were used were kept dry, the desired cyclisation reaction also forms water as a by-product. Therefore, toluene, a Dean–Stark apparatus and a nitrogen bubbler were used to aid in the removal of the formed water which also helped drive the reaction to form cyclics. Other strategies to remove water, such as the addition of 3 Å molecular sieves, were assessed, but these changes lowered yields. This result may be due to the sieves’ capacity to absorb the alkali ions, thereby hindering the reaction. The choice of base also proved to be important. Whilst a potassium carbonate base delivered modest yields, MCO yields were greatly improved by using cesium carbonate instead, with a large reduction in the amount of linear polymer by-product being produced and subsequently removed during Soxhlet purification. This is likely due to the “cesium effect”, where it has been noted that cesium is more likely to promote intramolecular reactions and cyclisations, especially in dipolar aprotic solvents [19]. These investigations allowed for an improvement in yields, and the use of pseudo-high dilution allows for the reaction to be somewhat scalable, though further reaction optimisation would be required to achieve the higher percentage yields that are acceptable in industry applications.

Macrocyclic oligomers of both *p*-PEKK and *m*-PEKK were successfully formed under the same reaction conditions; however, the *m*-PEKK MCOs were noted to form in higher yields and had greater solubility in NMP and chloroform, with *p*-PEKK MCOs having poor solubility in most solvents at room temperature and only being readily soluble in a mixture of 5% TFA in either DCM or chloroform. This is exemplified by the MALDI-ToF mass spectra for both products, with *m*-PEKK MCOs showing a higher range of cyclic oligomers. The equivalently sized *p*-PEKK oligomers are likely lost during the Soxhlet extraction purification due to the aforementioned poor solubility. Complete separation and purification of the individual MCO sizes also proved exceedingly difficult, with incomplete separations resulting from both flash column and Sephadex LH-20 size exclusion chromatography (SEC). As all MCOs have the same repeating units, it should be expected that their polarities will be very similar, as evidenced by very little separation on thin-layer chromatography (TLC), thereby hindering purification by flash column chromatography, while the insolubility of PEKK and its MCOs in many common solvents was a key impediment to separation by SEC. The inability to separate the MCOs would not necessarily be adverse to the polymerisation; however, theoretically, all homologous MCOs should be equally able to participate in ring-opening, and indeed, the mixture of sizes can lower melting points to allow for milder polymerisation conditions [8]. The exception to the difficulty with separation was the *m-*PEKK MCO dimer, which demonstrated sufficiently different solubility from the larger oligomer sizes such as to be purified during work-up as a separate precipitate. The dimer was also shown to be soluble in acetonitrile, whereas the larger *m-*PEKK MCOs were not.

#### 3.1.1. MALDI-ToF Results

**Figure 1:**
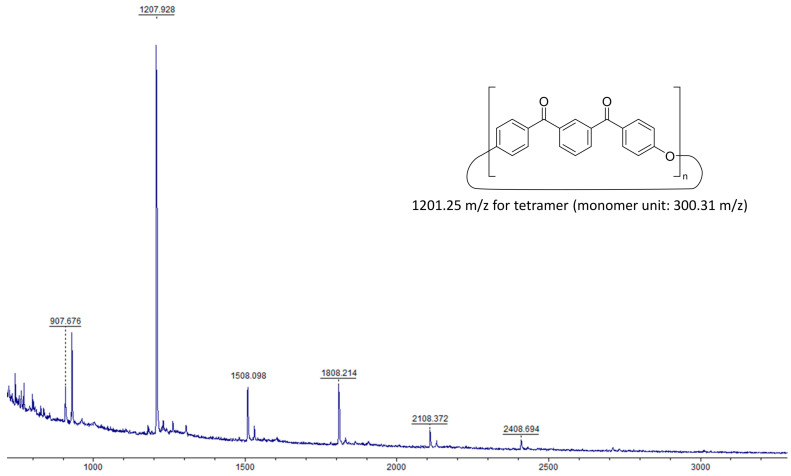
MALDI-ToF spectrum of *m-*PEKK MCOs (**3**) with LiBr cationising agent. [M(cyclic monomer unit)] calculated for C_20_H_12_O_3_ = 300.08 *m*/*z*. [M(tetramer)+Li]^+^ calculated for C_80_H_48_O_12_Li = 1207.33 *m*/*z* and found as 1207.38 *m*/*z*.

Analysis by MALDI-ToF was found to provide the best response when using 2,5-dihydroxybenzoic acid (DHBA) as a matrix with silver TFA salt or LiBr as a cationising agent. It should be noted that while the reactions in Scheme 3 and Scheme 5 would be expected to only yield cyclic oligomers containing an even number of monomer units, i.e., the dimer, tetramer, etc., the MALDI-ToF results (Figure 1) show this not to be the case with macrocycles consisting of odd numbers of monomer units, in particular, with the trimer and pentamer also being present.

**Figure 2:**
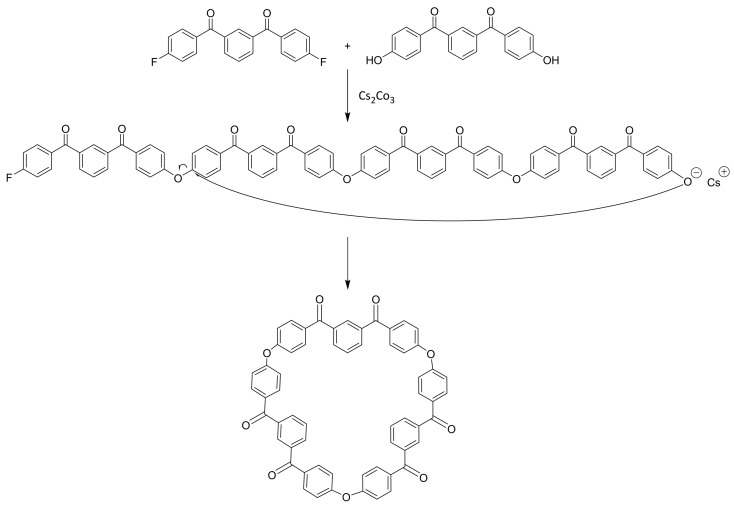
Mechanism of back-biting reaction responsible for presence of odd-numbered oligomers.

While it is possible that these odd-numbered MCOs came about from the reaction of a difluoro monomer with an even-numbered oligomer that has been hydrolysed by formed water, these hydrolysed oligomers were not identified through NMR spectroscopy nor MALDI-ToF mass spectrometry. It is instead proposed that these MCOs with odd repeat units form via a back-biting mechanism (see Figure 2), either during ring formation [12] or by subsequent ring-opening action from the cesium carbonate base, [14] which has been noted to also act as an initiator of ring-opening polymerisations [15]. The odd-numbered cyclic oligomers have less magnitude on both MALDI-ToF and GPC, in keeping with the fact that the transetherification pathway proceeds more slowly than the nucleophilic substitution ring-closing reaction.

#### 3.1.2. X-Ray Crystallography

**Figure 3:**
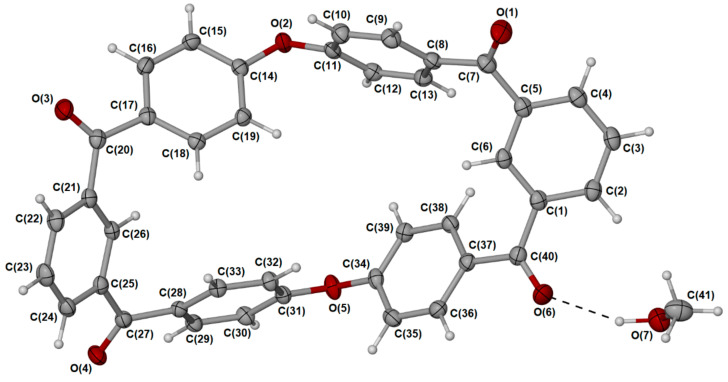
Single-crystal X-ray structure of *m-*PEKK MCO dimer (**3**) with adjacent methanol where the dark (dark red) components represent the oxygen molecules present.

**Figure 4:**
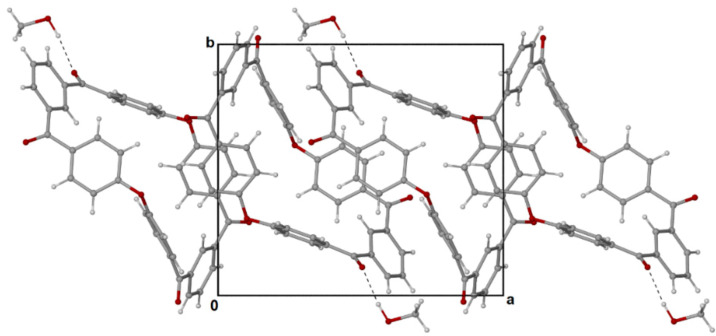
Successive molecules of dimer (**3**) crystalise by stacking in columns parallel to the a-axis (in square outline to show a-b axis) where the dark (dark red) components represent the oxygen molecules present.

The purity and solubility of the *m*-PEKK MCO dimer allowed for it to be dissolved in acetonitrile and for brown, needle-like crystals to be grown by vapour diffusion with methanol. These crystals proved amenable to single-crystal X-ray analysis, revealing a flat-ring structure (Figure 3) that crystallises in a monoclinic system where successive molecules stack into parallel columns (Figure 4).

**Figure 5:**
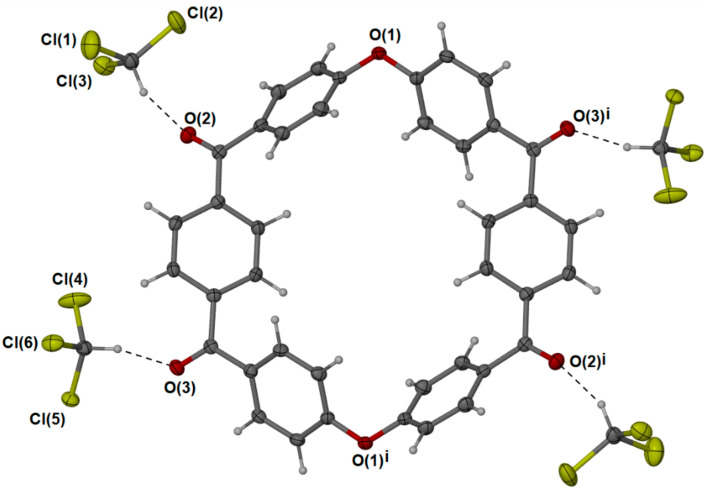
Single-crystal X-ray structure of *p-*PEKK MCO dimer (**6**) with adjacent chloroform.

**Figure 6:**
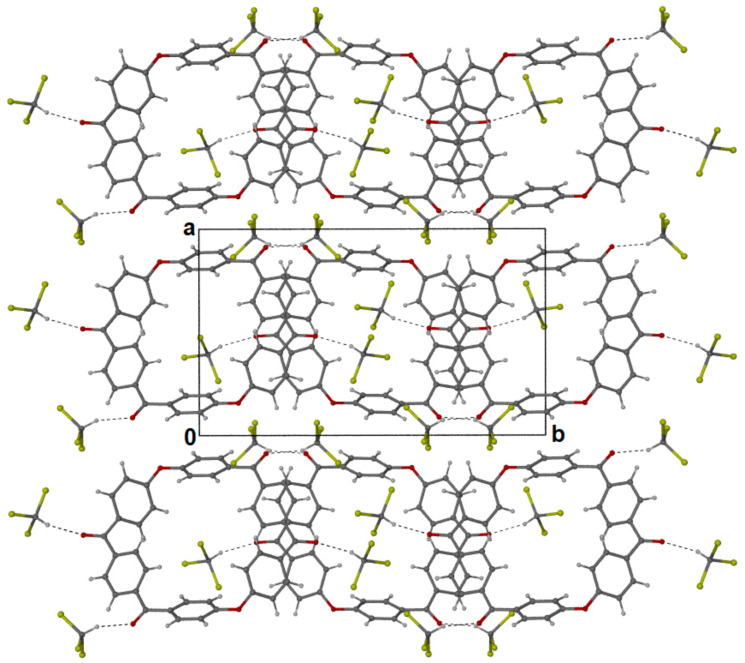
Orthogonally spaced groups of parallel-stacked *p-*PEKK dimers (**6**). The square represents the a-b axis with respect to 0.

As previously mentioned, the *para* PEKK cyclics proved to be insoluble in most common solvents, with attempts to form crystals from a solvent mixture of 5% TFA in chloroform by vapour diffusion with antisolvent or by cooling proving unsuccessful. However, some flat, orthogonal crystals were successfully grown by the very gradual cooling of the bulk Soxhlet solution. Similar to the *meta* PEKK, these crystals were composed of the dimer (Figure 5), with an even-more-planar ring structure in a monoclinic system. The increased flatness of the *p*-PEKK dimer over the *m*-PEKK allows for increased π stacking (Figure 6) and contributes to the *p*-PEKK’s insolubility in most solvents.

### 3.2. Adaptation for Industry-Relevant T/I Blends

While the main body of this work was focussed on producing the novel MCOs of a PEKK homopolymer for further polymerisation, it was also noted that the established synthesis method could be altered to produce MCOs with T/I ratios already commonly used in industry applications. To explore this, several reactions were carried out to form MCOs with 60/40, 70/30 and 80/20 T/I compositions. These syntheses utilised the same reaction conditions as used for the *m*-PEKK and *p*-PEKK MCOs, with the amount of 1,4-phenylenebis((4-fluorophenyl)methanone) (**4**) remaining the same and a ratio of 1,4-phenylenebis((4-hydroxyphenyl)methanone) (**5**) and 1,3-phenylenebis((4-hydroxyphenyl)methanone) (**2**) appropriate to the desired blend, as shown in Table 2.

**Figure 7:**
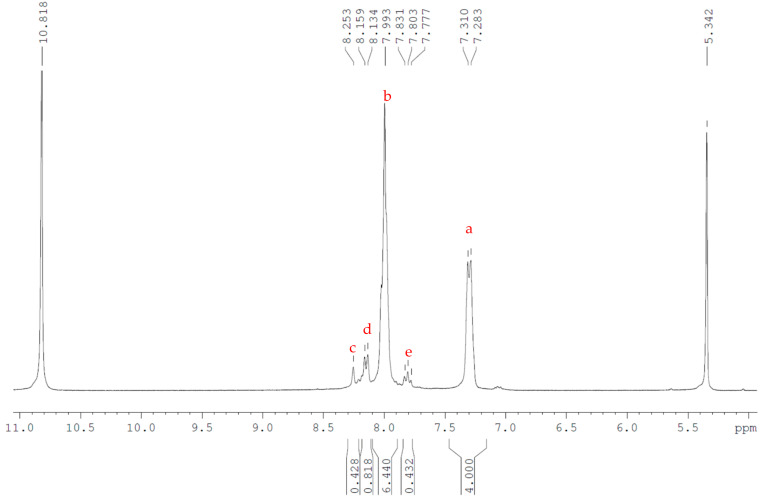
^1^H NMR (300 MHz) of 60/40 T/I PEKK MCOs (**7**) with 10% TFA-d (10.8 ppm) in DCM-d_2_ (5.34 ppm) as solvent. Peaks were annotated with respect to the structure shown.

**Figure 8:**
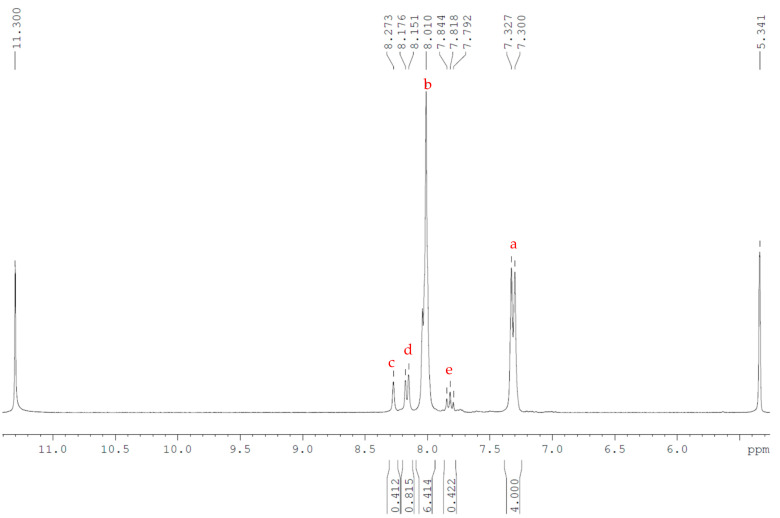
^1^H NMR (300 MHz) of Arkema 6002 PEKK, with 20% TFA-d (11.3 ppm) in DCM-d2 (5.34 ppm) as solvent. Peaks were annotated with respect to the structure shown.

The MCO mixture produced in this way cannot be homologous and consists of ring products with both varying sizes and differing amounts of *para* and *meta* linkages. Some products may be more energetically favoured; for example, molecular modelling of energy minimisation of the tetramer found that a compound with two alternating *para* and *meta* linkages is more favoured than either molecule consisting of entirely either *meta* or *para*, but due to the ratio of starting materials and the aforementioned back-biting reactions, the bulk product should be expected to be a diverse mixture. This provides no adverse effects to the polymerisation nor the final polymer, as an ED-ROP reaction will result in a PEKK polymer with a randomised backbone analogous to that produced by a polymerisation with monomolecular starting materials. Analysis of this MCO product is more difficult than the isomerically pure ones, however, and hence was not the main goal of this work, as the larger number of differing structures renders GPC and X-ray crystallography analysis much more difficult. MALDI-ToF analysis remained unimpaired, with each product forming oligomers of the same size as noted for the *meta* and *para* products and dimer and tetramer oligomers having greater intensities than the trimers or pentamers. NMR analysis was also used to prove that no monomer isomer had been wholly excluded from the reaction and that the bulk product consists of the desired T/I ratio (Figure 7 and Figure 8). The peak at 7.31 ppm, which corresponds to the position adjacent to the ether linkage (environment a), is common to both the *meta* and *para* monomer unit, and, therefore, by calibrating the integral of this peak to the four hydrogens present per monomer unit, the relative abundance of the isomers can be established by the change in the integrals of the other peaks. As illustrated in Figure 7, the peak at 7.80 ppm (for the 60/40 T/I MCO product), corresponding to the single-proton environment of the *meta* to the ketone linkage, which is only found in the *meta*-PEKK (environment e), would be expected to have an integral of only 0.4, being a single hydrogen that is only present in 40% of the monomers.

Following this methodology, all three blends were shown to be close to the predicted T/I ratio in the bulk MCO material (Table 2). This method was also assessed. NMR analysis of the two commercial grade PEKK samples produced by Arkema’s Kepstan^®^ 6002 and 7003, (which have T/I ratios of 60/40 and 70/30, respectively), with the specimens dissolved in 20% TFA-d in DCM-d_2_. As the linear polymer and cyclic oligomers are identical in terms of functional groups, the spectra of the formed MCOs and the related commercial product would be expected to be same and are indeed highly similar, albeit with some loss of sharpness in the peaks of the MCOs due to restriction of molecular movement as a result of the ring system. Similar analysis of the peak integrals of the commercial products also confirmed that the *meta* and *para* linkages were present in the advertised ratios (Figure 8).

### 3.3. Polymerisation and DSC

Initial investigations into the ED-ROP polymerisation were carried out with DSC and TGA, in order to confirm that the MCOs would ring-open and that some degree of polymerisation would occur. Cesium fluoride was chosen as the polymerisation initiator, due to its previous demonstrated use as an ROP initiator for PAEK compounds [15], its commercial availability and its tolerance of high temperatures, and was added to samples at 2 mol% per monomer unit (i.e., 0.101 mg of initiator for 10 mg of MCOs). The samples were suspended in DCM with 10% methanol and were sonicated, and the solvent was evaporated to ensure an intimate mixture of the sample and initiator, and the samples were then dried under vacuum conditions to limit the presence of water. DSC measurements were also taken for samples of Arkema Kepstan^®^ 6002 and 7003 to act as points of comparison.

**Figure 9:**
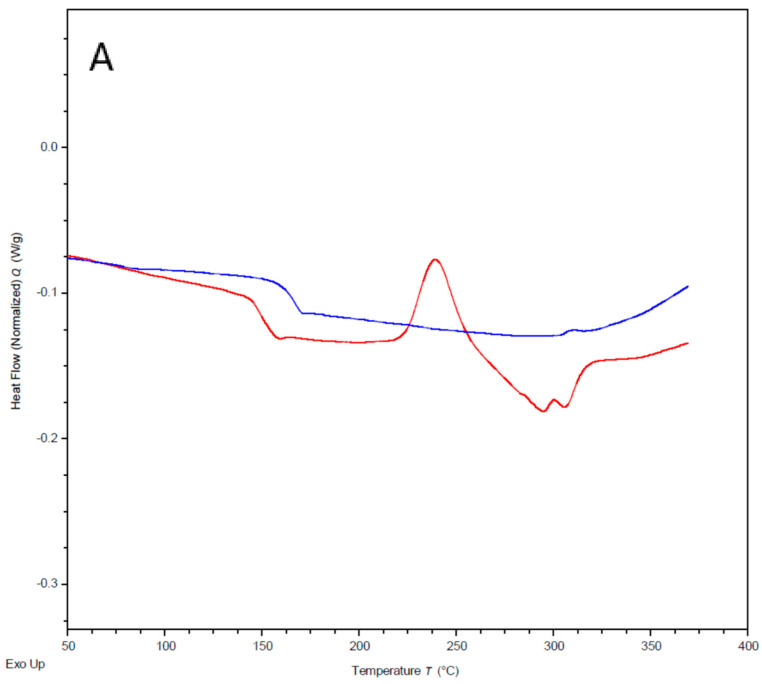

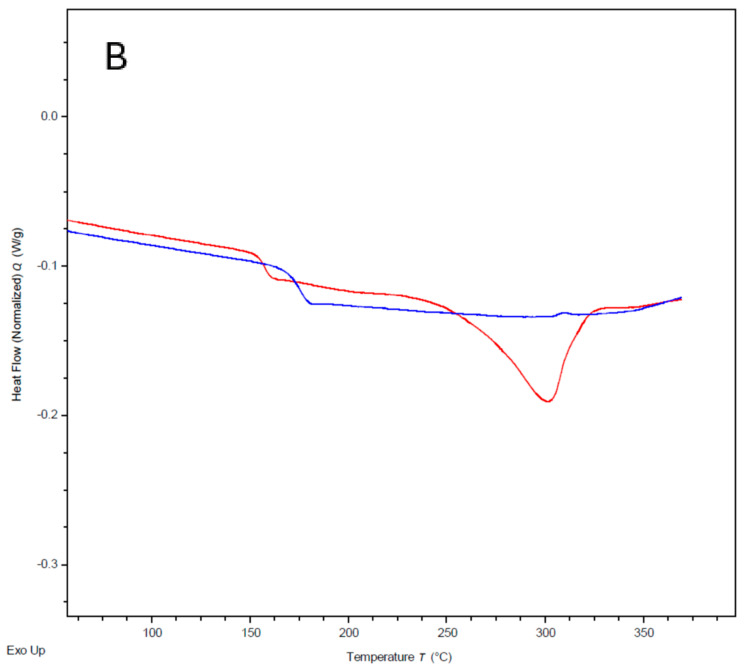
DSC comparison of Arkema 6002 (red) with MCO-derived 60/40 T/I PEKK (blue) for first (**A**) and second (**B**) consecutive heating cycles to 350 °C at a rate of 5 °C/min, demonstrating a similarity in glass transitions.

DSC analyses were performed under nitrogen with a thermal scan up to 400 °C and then back to 25 °C with a temperature rate change of 5 °C/min. Samples that had been mixed with the initiator and recovered after this cycle were found to have changed from a powder into a tough, brown film that was insoluble in all common solvents including DCM, which the MCOs had originally been soluble in. This material was also now completely insoluble in chloroform, while samples without the initiator remained somewhat soluble. The thermal cycle was then repeated to analyse the thermal properties of the now-formed polymer. Both *m*-PEKK and *p*-PEKK MCOs displayed the appearance of a glass transition on the second thermal cycle which, combined with changes to the material’s morphology and solubility post heating, were similar to other MCO-derived PAEKs [12], indicating the formation of a polymer. This analysis was then also performed on the MCOs with varying T/I ratios, and the glass transition temperature of the Arkema Kepstan^®^ 6002 and the polymer product of the 60/40 T/I MCO compared favourably with each other (150–160 °C) and with reported values in the literature [7] (Figure 9). Importantly, the Arkema sample also displayed a large cold-crystallisation peak at 142 °C on the first thermal cycle which was not present in the polymerised MCOs and a broad crystalline melting point on the second cycle, while the PEKK formed from MCOs only yielded small and broad melting points. This result, however, was not unexpected given the large variability that PEKK crystallinity can have based on thermal history [7], impurities from the synthesis, remnant unpolymerized MCOs and the CsF initiator and the potential for cross-linking after repeated thermal cycling [20]. In addition, while both the PEKK formed from MCOs and the commercial product have a T/I ratio of 60/40, as demonstrated with NMR, the distribution of these linkages may be different, as the Arkema sample is synthesised by a proprietary method while the MCO chain would be further randomised by back-biting activity by the polymerisation initiator. As such, the ED-ROP polymer may be much more amorphous. These effects are likely responsible for the differences in colour between the PEKK formed from MCOs and the commercial product.

All other samples were tested with the same method, with all displaying the appearance of a glass transition after heating, albeit with some degree of variance due to their thermal histories, structures and small variances in the amount of initiator, and are displayed in the Table 3.

**Figure 10:**
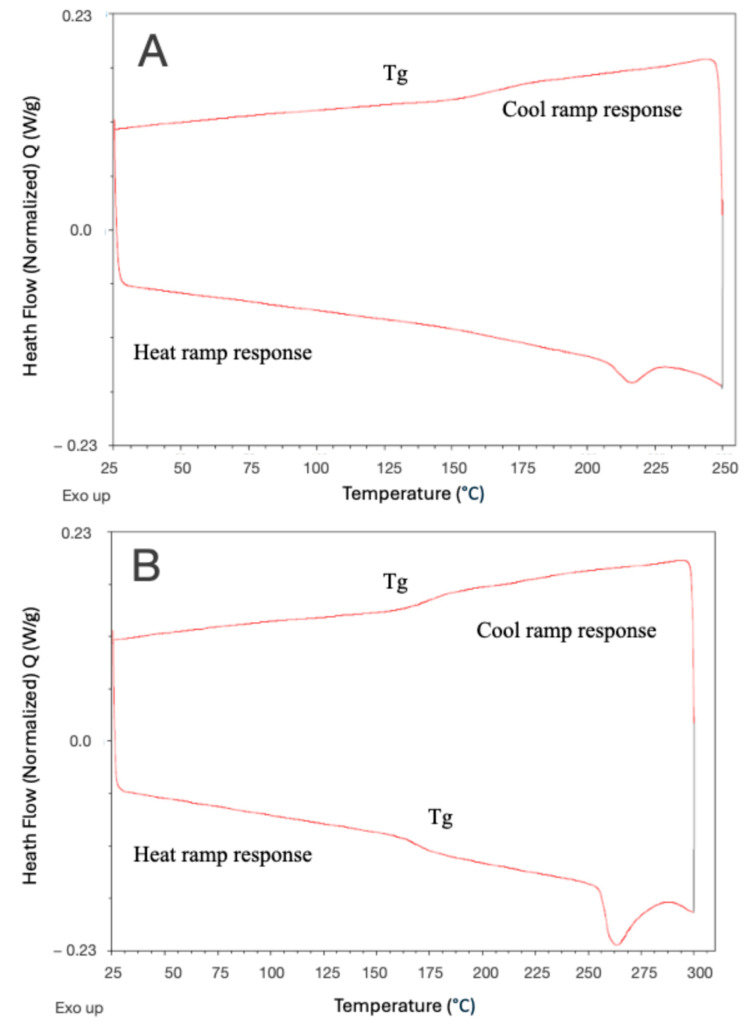
DSC results of heating a sample of 60/40 T/I PEKK MCO (**7**) with a CsF initiator to 250 °C and then cooling and subsequently heating to 300 °C and cooling again, showing (**A**) Tg on the initial heat ramp, with the appearance of a glass transition when heated above 250 °C on the cooling response in and (**B**) in both the heat and cooling response.

The transetherification-based polymerisation mechanism is expected to be possible at temperatures around 150 °C, as the back-biting reaction previously observed also proceeds by the same mechanism. However, in the absence of solvent, the MCOs must first melt before they are able to react, and the forming polymer chains must remain melted to participate in chain extension. Therefore, while the optimal temperature for polymerisation is likely above the melting point for the PEKK polymer, further exploration of the temperature at which the polymerisation initiates was warranted. As ED-ROP should not release heat during polymerisation, it remains difficult to examine by DSC. To address this, a fresh sample of the same 60/40 T/I MCO with the initiator was cycled through increasing temperatures of 50 °C increments, with the emergence of a glass transition during cooling being used to determine when the polymerisation had taken place (Figure 10A,B). A glass transition at 165 °C appeared once the sample had been heated to 250 °C, indicating that the polymerisation begins between 200 and 250 °C and corresponding with a suspected macrocycle melting point at 216 °C observed on the sample with no initiator. Upon heating to higher temperatures, this glass transition increased in temperature, demonstrating an increase in polymerisation as nascent polymer is able to stay in a melted state. Thermal experiments were also conducted on the isolated *m*-PEKK cyclic dimer and *m*-PEKK MCOs with the dimer removed to investigate whether it has an adverse effect on polymerisation, as has been found in previous PAEK MCO studies [13]. TGA/DSC analysis of the *m-*PEKK dimer noted that the material melts at 417 °C (see Supporting Information), which is also within the region where significant weight loss due to thermal degradation begins to occur. More complete polymerisations were also performed with the dimer removed, suggesting that the dimer is not sufficiently solvated by the other melted MCOs as to be able to participate in the ED-ROP.

As ED-ROPs involve the re-arranging of cyclic rings into linear chains by the favourable change in entropy, there is no change in the weight of the material before or after polymerisation and by-products released by the reaction. To examine this with PEKK MCOs, a series of polymerisations were carried out with TGA analysis to demonstrate the lack of volatiles that evolved or changed in weight. The polymer product was then removed from the crucible and found to be a tough, glass-like material which was no longer soluble in chloroform (as the MCO was) and only partly soluble in TFA. As expected, almost no weight loss was observed during this heating cycle (see Supporting Information).

### 3.4. GPC Analysis of Polymers

**Scheme 8:**
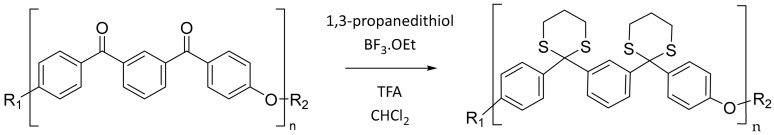
Thioketal derivatisation of *m-*PEKK.

Larger scale polymerisations were conducted to better analyse the optimal polymerisation conditions for the *m*-PEKK MCOs. GPC analysis was necessary to assess the degree of polymerisation of the MCOs; however, this proved difficult due to the poor solubility of PEKK in most common GPC solvents, often requiring exotic solvent systems such as chlorobenzene/phenol to be used. Instead, a strategy of derivatising of the polymer to a more soluble form was employed (Scheme 8), utilising a thioketalisation methodology reported by Colquhoun et al. [21] and described in the Methods Section. By fully converting the polymer into a derivative soluble in chloroform, GPC could successfully be performed, and the molecular weights from this analysis could be corrected for the conversion, giving the molecular weight of the original PEKK polymer.

**Figure 11:**
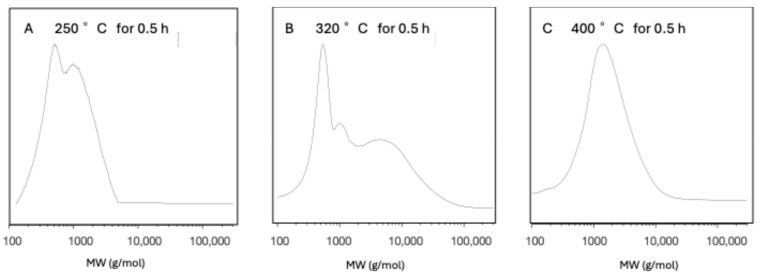
GPC chromatograms of MCO ED-ROP experiments conducted within a DSC pan with a 2 mol% CsF initiator, demonstrating that heating to 320 °C (**B**) could form a higher-molecular-weight polymer shoulder. Heating to >400 °C (**C**) or <300 °C (**A**) yielded chromatograms with molecular weights similar to the starting MCOs, though in the samples heated at higher temperatures, there was a lower detector response and much more insoluble material had been filtered off.

This reaction was successfully applied to the Arkema sample, resulting in a white plastic which was freely soluble in chloroform. ^13^C NMR analysis showed a complete conversion of the thioketal through the disappearance of the ketone carbonyl peak, allowing further analysis to be easily corrected to account for the additional mass. A series of polymerisations with a 2 mol% CsF initiator were then conducted within the nitrogen atmosphere of the DSC with the goal of exploring the optimal temperature and heating time required to form PEKK polymers by ED-ROP. These samples where then thioketalised, and their degree of polymerisation was assessed by GPC. However, while there was some evidence of polymerisation from the formation of a higher-molecular-weight shoulder in samples heated to 300–350 °C, no molecular weights comparable to those in the commercial polymer were observed (Figure 11). Samples heated to or above 400 °C largely produced a charred or crosslinked material, which was insoluble in the 20% TFA in the DCM solvent mixture that was being used for the thioketalisation reaction and that could successfully dissolve commercial PEKK. As such, GPC analysis of these materials did not show much evidence of polymerisation, with it being likely that any ring-opened polymer material had likely been filtered out during work-up or before injection into the GPC. In contrast, samples heated to below 300 °C likely had nascent polymer that resolidified at lower molecular weights.

**Figure 12:**
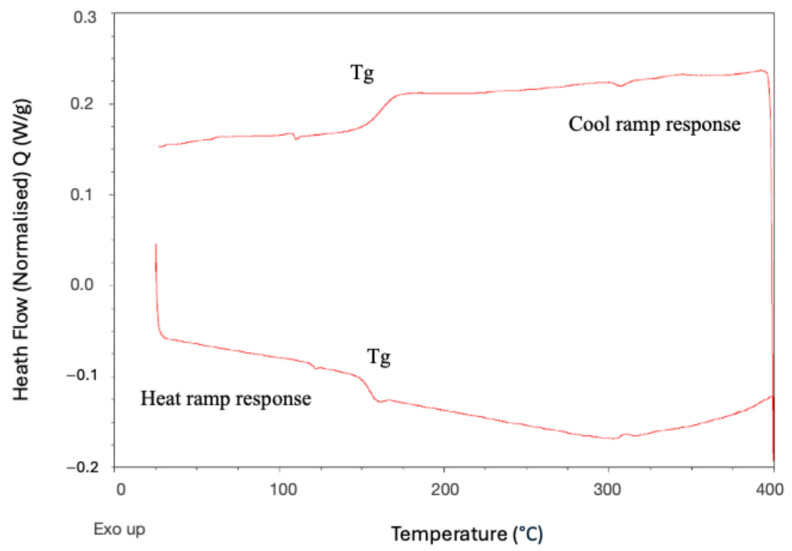
DSC of *m-*PEKK formed under high-vacuum conditions, displaying a glass transition on heating (lower line) and cooling (upper line) similar to both previous DSC experiments and the commercial comparison.

Despite efforts to dry the materials and conducting the DSC measurements under nitrogen conditions, these initial samples that polymerised under DSC conditions did not yield high-molecular-weight polymers when analysed by GPC, so further polymerisation of the oligomeric samples was carried out under high-vacuum conditions to avoid the interference of water and oxygen during the transestherification and at higher temperatures in order ensure full mobility of the material, as has previously been reported for other PAEKs [22]. Based on the previous thermal analyses, the samples used were of *m*-PEKK MCOs with the dimer already removed. DSC of the material polymerised in this way was conducted under the same conditions as previous experiments and displayed a similar glass transition temperature (Figure 12).

**Figure 13:**
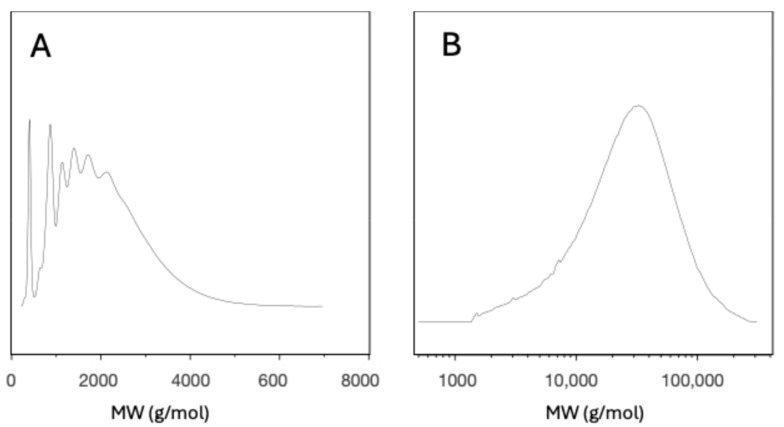
GPC chromatogram of (**A**) *m-*PEKK MCO sample and (**B**) derivatised *m-*PEKK polymerised from MCOs under high-vacuum conditions (runcorrected for derivatisation).

GPC analysis of these compounds could then be successfully performed, with the polymer formed from MCOs under the high-vacuum conditions being compared to both the starting oligomers (Figure 13) and the commercial sample (see Supporting Information). GPC of the polymerised *m*-PEKK, once corrected for the derivatisation and detector response, showed that the number average molar mass had increased to 22,000 g/mol. This compares favourably with the corrected M_n_ of the Arkema 6002 sample, although the commercial sample had a much higher M_w_ and greater polydispersity (Table 4). These results, along with the previously discussed appearance of a glass transition and change in solubility of the material, demonstrate a successful ED-ROP of the MCOs into PEKK. Optimisation of this polymerisation, by both investigation of the conditions and the initiator, will form the basis of future work.

## 4. Conclusions

The synthesis of two novel groups of compounds, the MCOs of *p*-PEKK and *m*-PEKK, was successfully achieved with the reaction optimised and extended to form MCOs with T/I ratios analogous to those of polymers used in industry applications. Despite the synthesis pathway initially only allowing for even-numbered oligomers, odd-numbered oligomers were observed in lower intensities through MALDI-ToF, indicating a simultaneous back-biting reaction. The macrocyclic dimers of both *m*-PEKK and *p*-PEKK were also successfully isolated, crystallised and analysed by X-ray crystallography. Methods were developed for the polymerisation of MCO samples by ED-ROP and their subsequent analysis by GPC, with initial results of DSC and GPC showing promising similarities to the industrial counterparts and providing an avenue for ongoing work into the full investigation of polymers formed by such methods and to their suitability for the *in situ* formation of high-performance thermoplastic composite.

## Figures and Tables

**Table 1:** Effects of carbonate and water removal by molecular sieves on the yield during MCO synthesis (compounds (**6**) and (**3**)).

Polymer	Purified Yield (%) Against Molecular Sieve Type			
*p*-PEKK MCO (**6**)	**K_2_CO_3_**	**K_2_CO_3_ + 3 Å Sieves**	**Cs_2_CO_3_**	**Cs_2_CO_3_ + 3 Å Sieves**
	22.6%	15.9%	41.0%	11.2%
*m*-PEKK MCO (**3**)	**K_2_CO_3_**	**Cs_2_CO_3_**		
	60.2%	91.4%		

**Table 2:** Starting material amounts and results of T/I blend MCO synthesis.

Desired T/I Ratio of MCO	1,4-Phenylenebis((4-hydroxyphenyl)methanone) (5)	1,3-Phenylenebis((4-hydroxyphenyl)methanone) (2)	Yield	NMR Determination of T/I Ratio
60/40	0.25 mmol	1.01 mmol	71.6%	57/43
70/30	0.50 mmol	0.75 mmol	84.3%	67/33
80/20	0.75 mmol	0.50 mmol	80.6%	85/15

**Table 3:** Results of DSC analysis of indicated MCO samples with 2 mol% CsF initiator.

MCO Sample	T_g_
*m*-PEKK (**3**)	151 °C
*p*-PEKK (**6**)	162 °C
60/40 T/I PEKK (**7**)	154 °C
70/30 T/I PEKK (**8**)	155 °C
80/20 T/I PEKK **(9**)	160 °C

**Table 4:** GPC results of derivatised *m-*PEKK and Arkema 6002 (values corrected to original polymer).

Polymer Sample	Peak	M_n_	M_w_	Polydispersity
*m*-PEKK	20,000 g/mol	22,000 g/mol	38,000 g/mol	1.77
Arkema 6002	37,000 g/mol	19,000 g/mol	46,000 g/mol	2.45

## Data Availability

The original contributions presented in the study are included in the article/Supplementary Materials, further inquiries can be directed to the corresponding author.

## Supplementary Materials

The following supporting information can be downloaded at https://www.mdpi.com/article/10.3390/polym16243465/s1, Characterisation spectra (^1^H, ^13^C, ^19^F and Heteronuclear Multiple Bond Correlation (HMBC) NMR, MALDI, IR, DSC, TGA and GPC). X-ray crystallography data deposited at CCDC 2348288 and CCDC 2348289.

## Abbreviations

Dichloromethane (DCM), deuterated dichloromethane (DCM-d_2_), deuterated chloroform (CDCl_3_), deuterated trifluoroacetic acid (TFA-d), dimethyl sulfoxide (DMSO), differential scanning calorimetry (DSC), hydrochloric acid (HCl), ln-methyl pyrrolidone (NMP), sodium hydroxide (NaOH), trifluoroacetic acid (TFA), thermogravimetric analysis (TGA), thin layer chromatography (TLC).
