# Supplementary material for: Synthesis of Cyclic Oligomers of Polyether Ketone Ketone (PEKK) for Ring-Opening Polymerisation (ROP) Applications
Source: Polymers (Basel). 2024 Dec 11;16(24):3465. doi: 10.3390/polym16243465 (PMC11728470; doi:10.3390/polym16243465)
Supplement: Supplementary file 1 [file polymers-16-03465-s001.zip › Supporting information for Synthesis of cyclic oligomers of polyether ketone ketone (PEKK) for ring-opening polymerisation (ROP) applications.pdf]

Supporting information

Synthesis of cyclic oligomers of polyether ketone ketone (PEKK) for ring-opening polymerisation (ROP) applications.

Characterisation information for 1,3-phenylenebis((4-fluorophenyl)methanone) (1)....... 4
NMR (^1H, ^{13}C, ^{19}F) spectra 4
IR spectrum 5
Characterisation information for 1,3-phenylenebis((4-hydroxyphenyl)methanone) (2)... 6
NMR (^1H, ^{13}C, ^{19}F) spectra 6
IR spectrum 7
Characterisation information for m-PEKK MCOs (5) and their thermal properties............ 8
NMR (^1H, ^{13}C, ^{19}F) spectra 8
MALDI-ToF spectrum 9
IR spectrum 10
GPC chromatogram 10
DSC spectrum 11
TGA spectrum of polymerisation 11
Characterisation information for 1,4-phenylenebis((4-fluorophenyl)methanone) (4)..... 13
NMR (^1H, ^{13}C, ^{19}F) spectra 13
IR spectrum 14
Characterisation information for 1,4-phenylenebis((4-hydroxyphenyl)methanone) (5). 15
NMR (^1H, ^{13}C, ^{19}F) spectra 15
IR spectrum 16
Characterisation information for p-PEKK MCOs (6) and their thermal properties........... 17
NMR (^1H, ^{13}C, HMBC) spectra 17
MALDI-ToF spectrum 18
IR spectrum 19
GPC chromatogram 19
DSC spectrum 20
Characterisation information for 60/40 T/I MCOs and their thermal properties.............. 21
NMR (^1H, ^{13}C, HMBC) spectra 21
MALDI-ToF spectrum 22
Characterisation information for 70/30 T/I MCOs and their thermal properties.............. 23
NMR (^1H, ^{13}C) spectra 23
MALDI-ToF spectrum 24
DSC spectrum 24
Characterisation information for 80/20 T/I MCOs and their thermal properties............. 25
NMR (^1H, ^{13}C) spectra 25
MALDI-ToF spectrum 26
DSC spectrum 26

Characterisation information of ARKEMA Kepstan 6002 for comparative purposes......... 27

NMR (^1H, ^{13}C, HMBC) spectra 27

Characterisation information for the thioketal derivative of the polymer produced from m-PEKK MCOs.. 29

NMR (^1H, ^{13}C) spectra 29

GPC (uncorrected for thioketal derivatisation).. 30

Characterisation information for the thioketal derivative of ARKEMA Kepstan 6002 for comparative purposes... 31

NMR (^1H, ^{13}C) spectra 31

GPC (uncorrected for thioketal derivatisation).. 32

Characterisation information for 1,3-phenylenebis((4-fluorophenyl)methanone) (1)

NMR (^{1}H, ^{13}C, ^{19}F) spectra

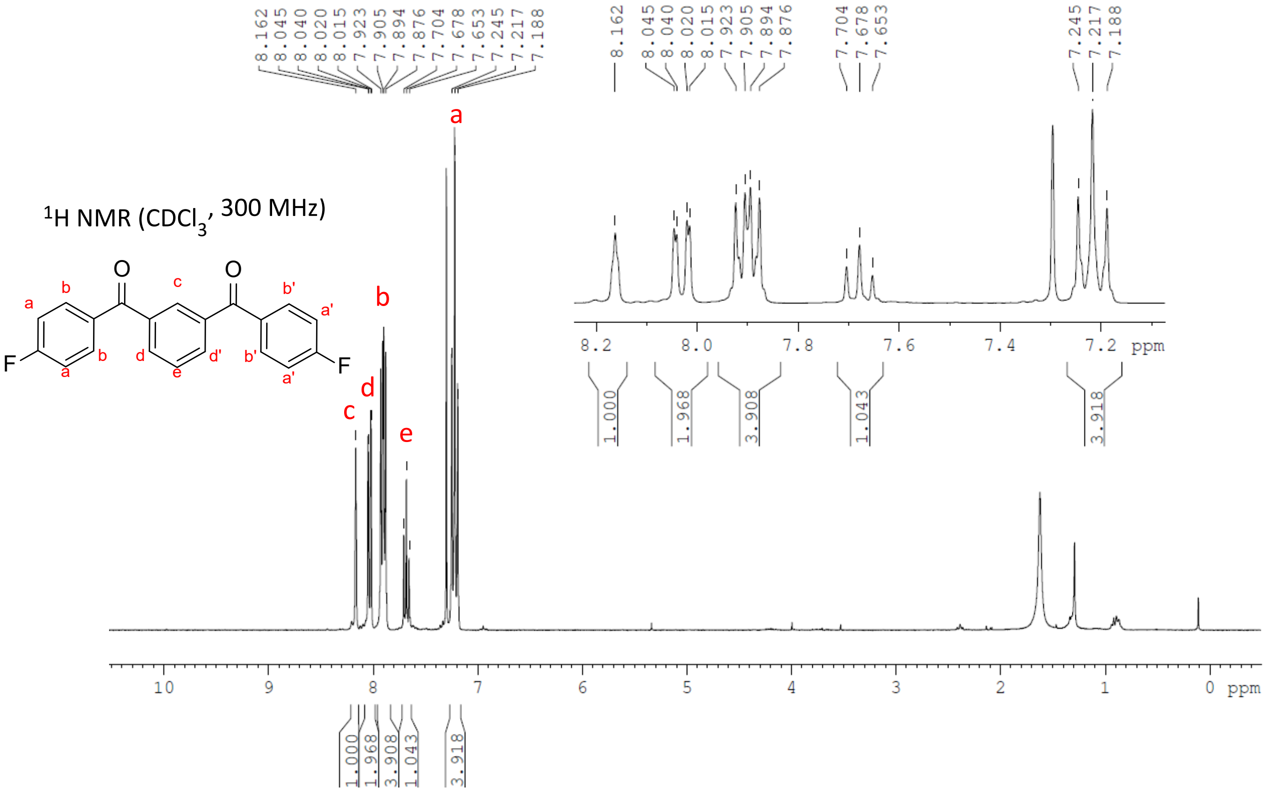

*Figure S1: ^{1}H NMR (CDCl3, 300 MHz) of 1,3-phenylenebis((4-fluorophenyl)methanone) (**1**)*

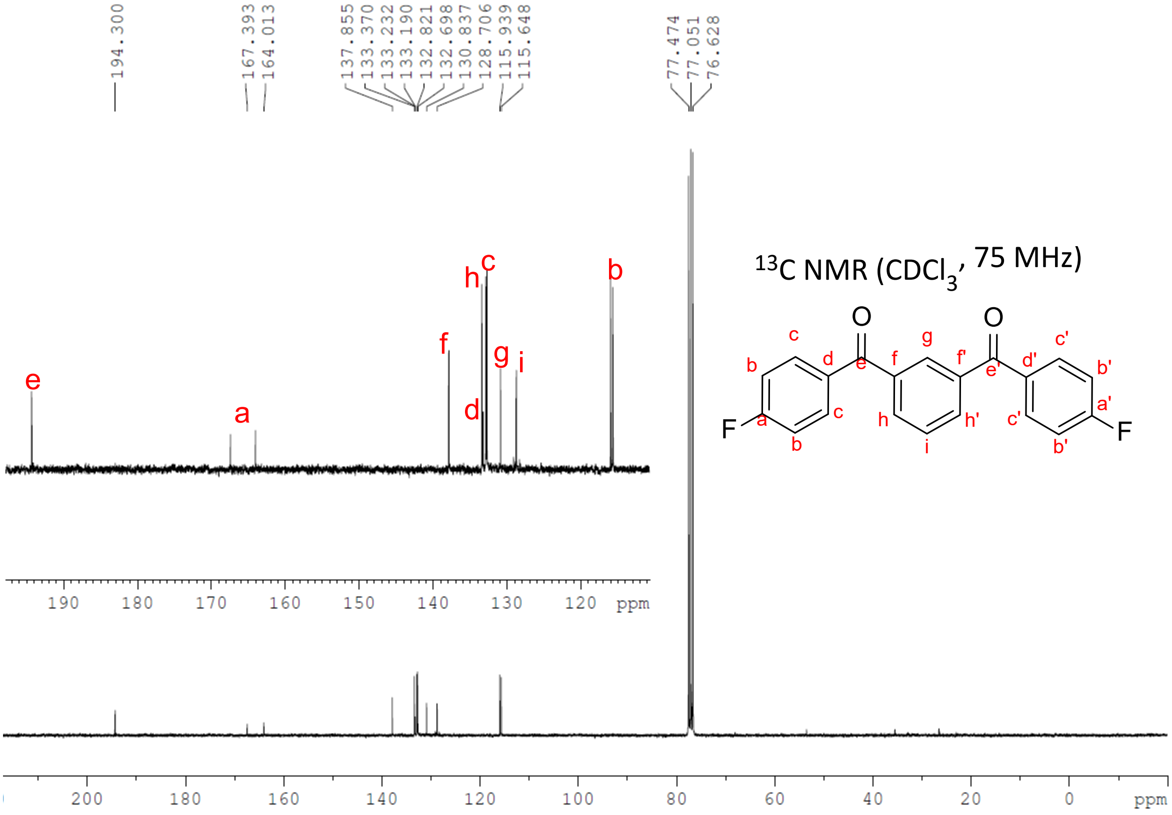

*Figure S2: ^{13}C NMR ($CDCl_3$, 75 MHz) of 1,3-phenylenebis((4-fluorophenyl)methanone) (**1**)*

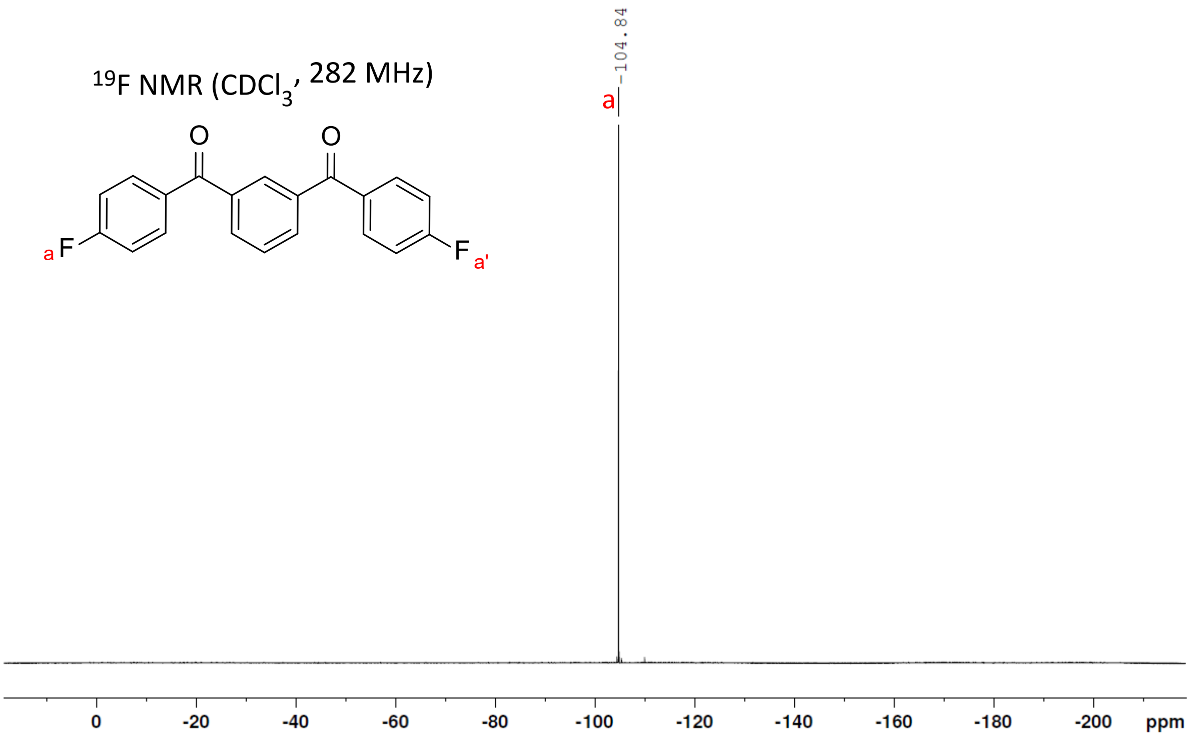

*Figure S3: ^{19}F NMR ($CDCl_3$, 282 MHz) of 1,3-phenylenebis((4-fluorophenyl)methanone) (**1**)*

IR spectrum

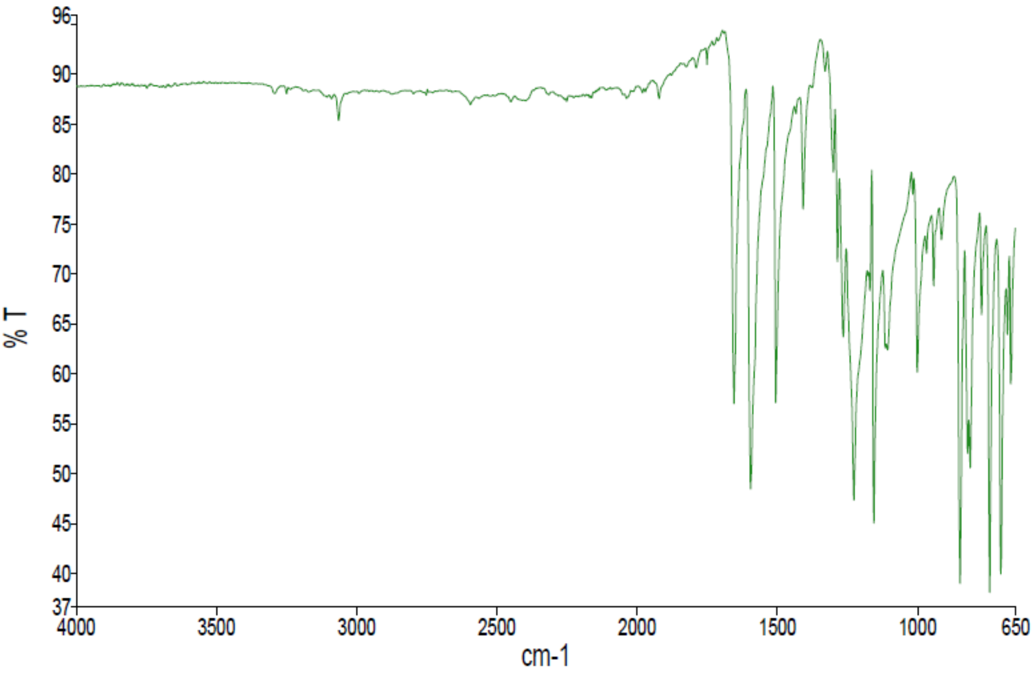

*Figure S4: IR spectrum of 1,3-phenylenebis((4-fluorophenyl)methanone) (**1**)*

Characterisation information for 1,3-phenylenebis((4-hydroxyphenyl)methanone) (2)

NMR (^{1}H, ^{13}C, ^{19}F) spectra

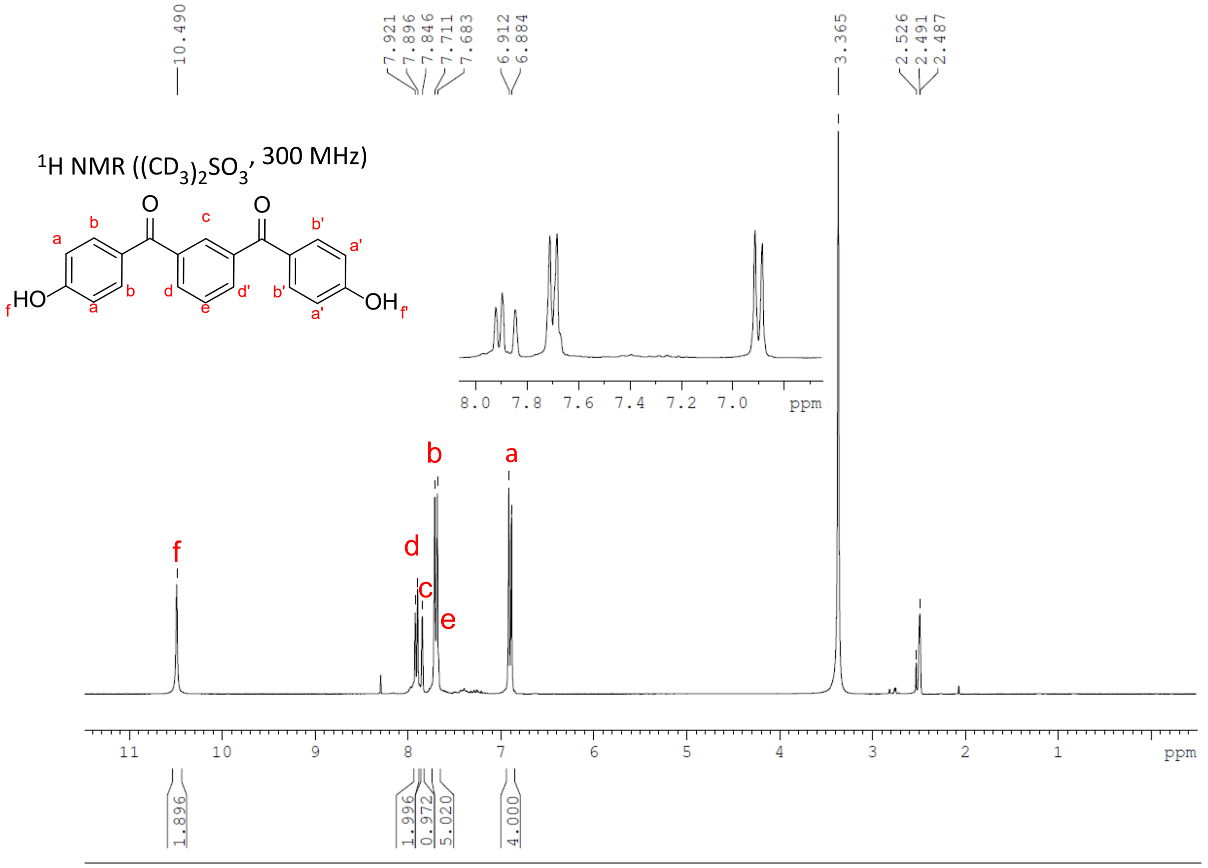

*Figure S5: ^{1}H NMR ($(CD_3)_2SO_3$, 300 MHz) of 1,3-phenylenebis((4-hydroxyphenyl)methanone) (**2**)*

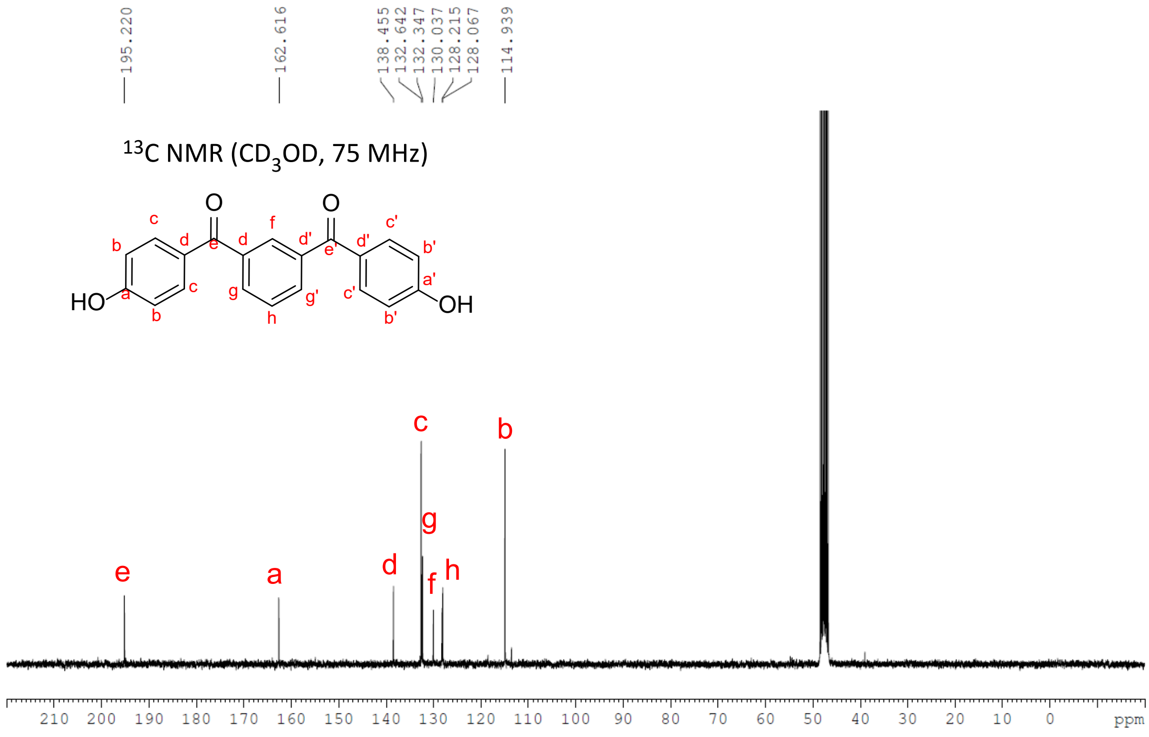

*Figure S6: ^{13}C NMR (CD_3OD, 75 MHz) of 1,3-phenylenebis((4-hydroxyphenyl)methanone) (**2**)*

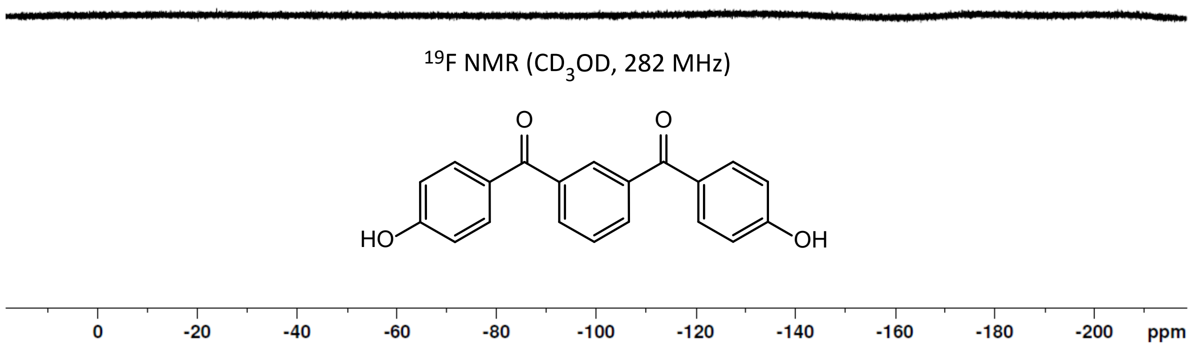

*Figure S7: ^{19}F NMR (CD_3OD, 282 MHz) of 1,3-phenylenebis((4-hydroxyphenyl)methanone) (**2**)*

IR spectrum

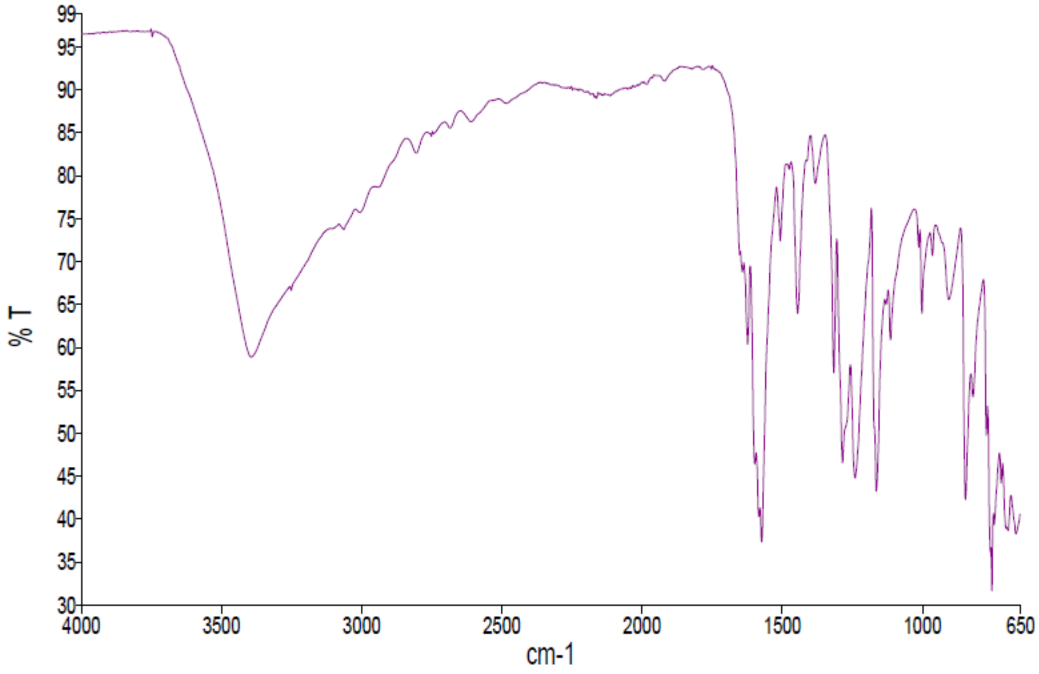

*Figure S8: IR spectrum for 1,3-phenylenebis((4-hydroxyphenyl)methanone) (**2**)*

Characterisation information for m-PEKK MCOs (5) and their thermal properties

NMR (^{1}H, ^{13}C, ^{19}F) spectra

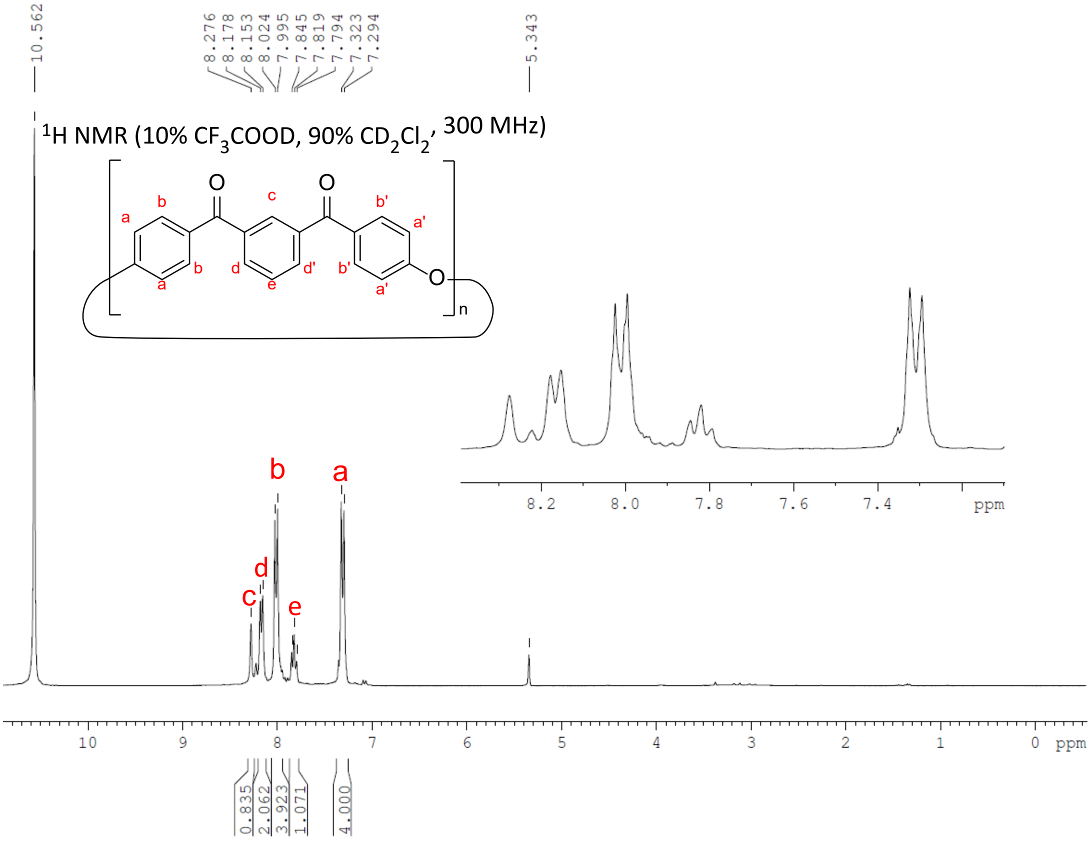

*Figure S9: ^{1}H NMR (10% CF_3COOD, 90% CD_2Cl_2, 300 MHz) of m-PEKK MCOs (**3**)*

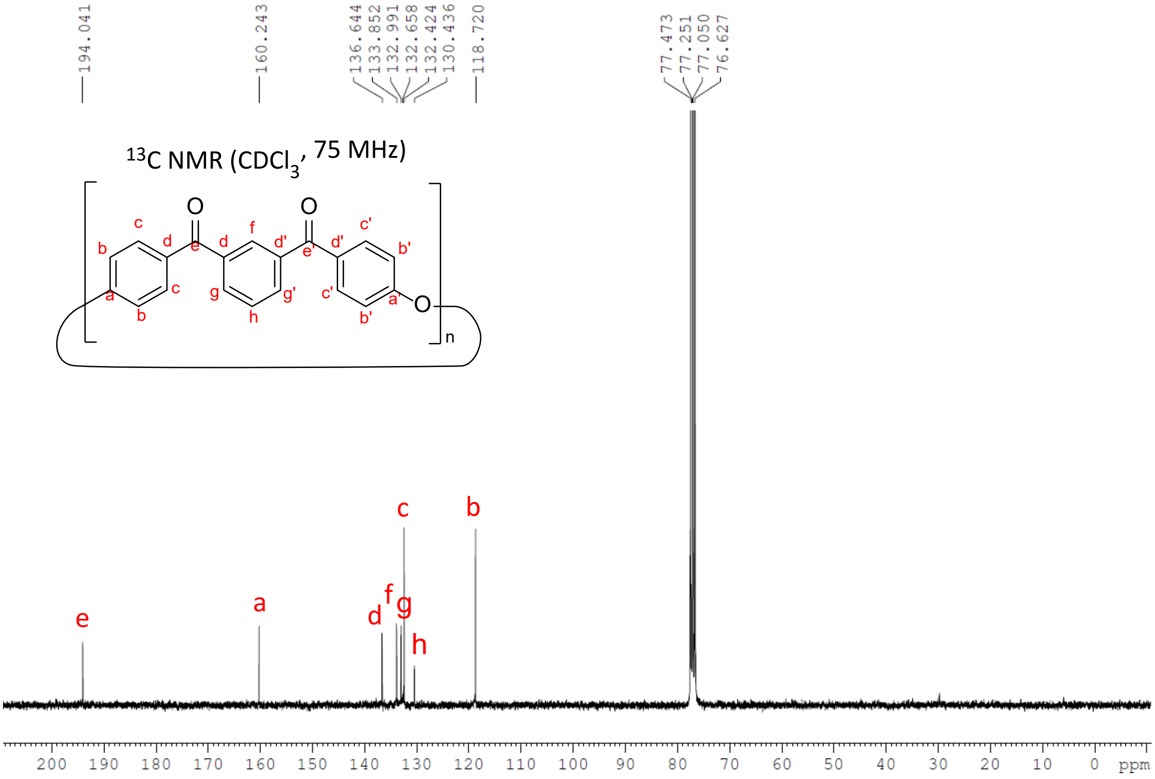

*Figure S10: ^{13}C NMR ($CDCl_3$, 75 MHz) of m-PEKK MCOs (**3**)*

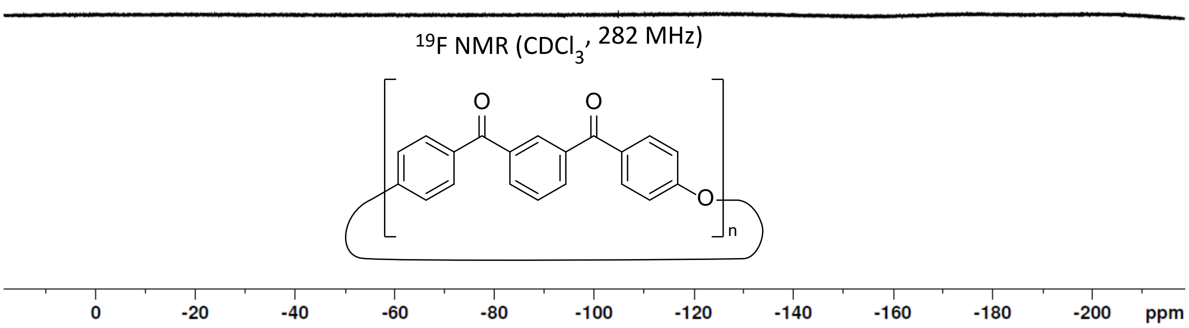

*Figure S11: ^{19}F NMR ($CDCl_3$, 282 MHz) of m-PEKK MCOs (**3**)*

MALDI-ToF spectrum

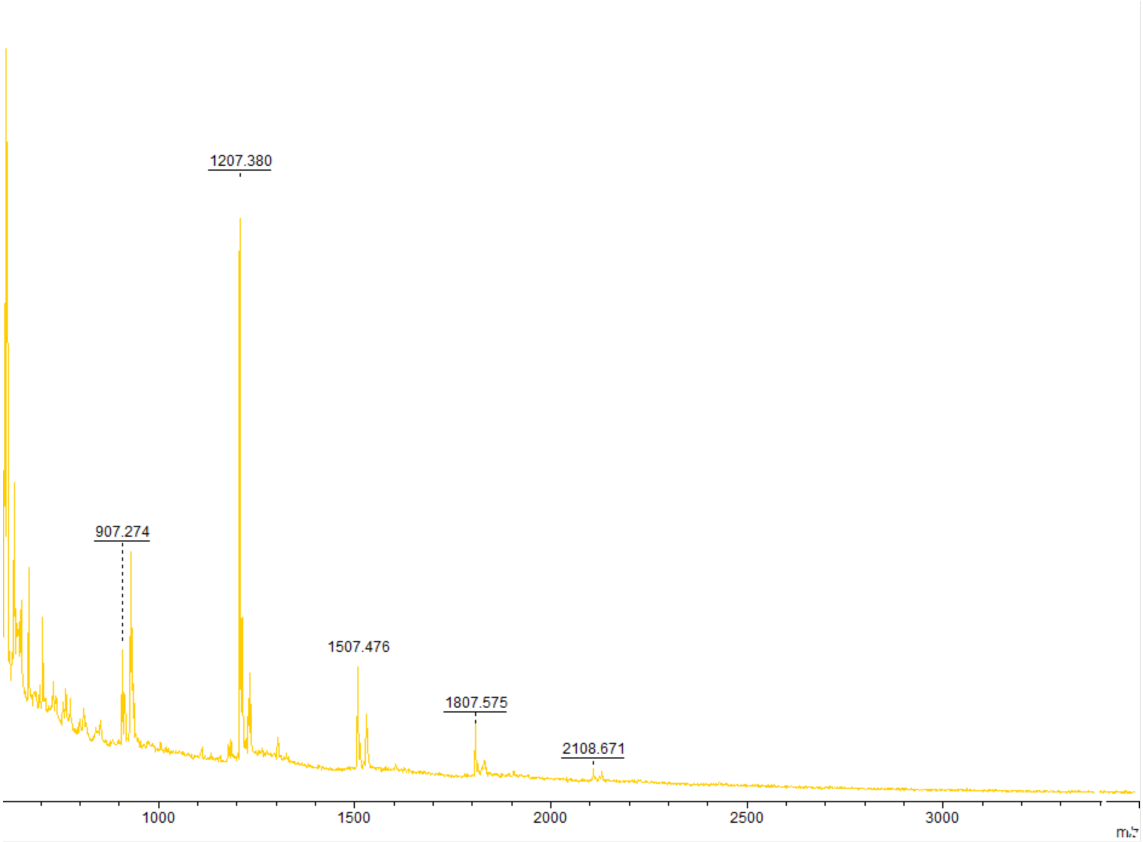

*Figure S12: MALDI-ToF spectrum of m-PEKK MCOs (**3**) with LiBr cationising agent. [M(cyclic monomer unit] Calcd for $C_{20}H_{12}O_3$=300.08 m/z. [M(tetramer)+Li]$^+$ Calcd for $C_{80}H_{48}O_{12}Li$ =1207.33 m/z, found 1207.38 m/z*

IR spectrum

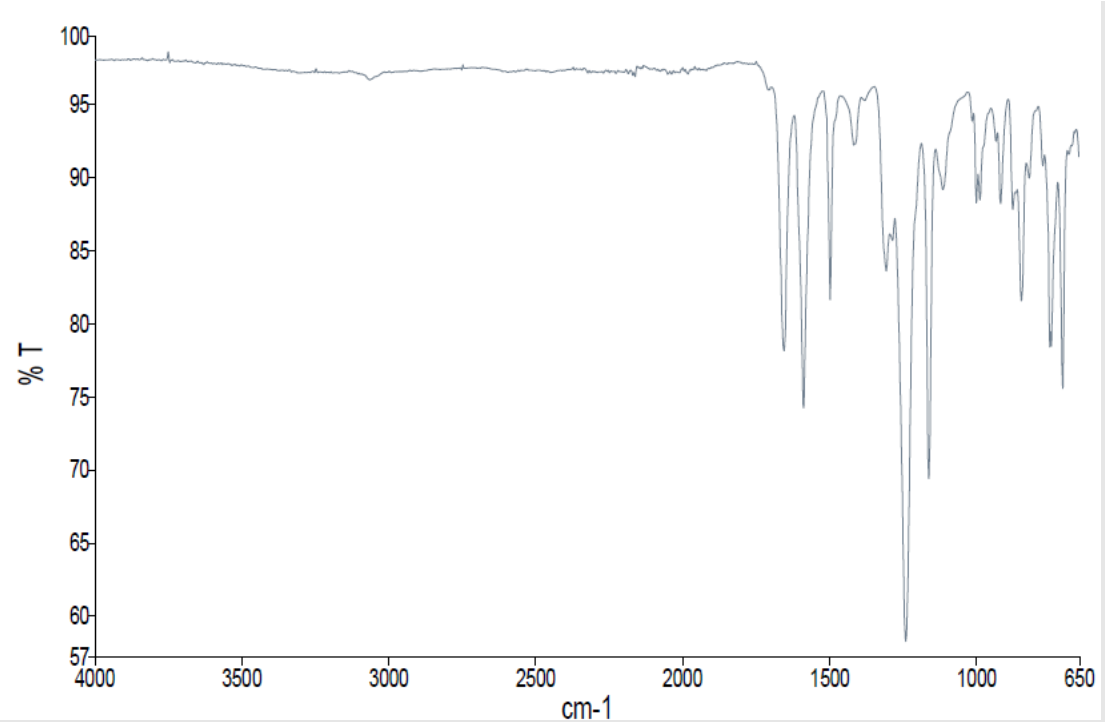

*Figure S13: IR spectrum of m-PEKK MCOs (**3**)*

GPC chromatogram

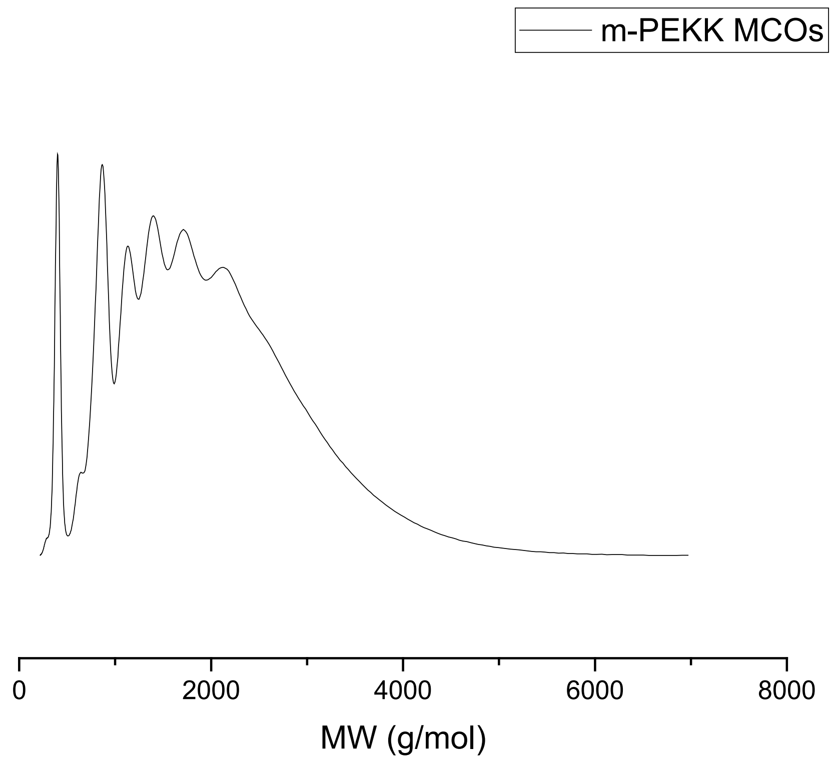

*Figure S14: GPC chromatogram of m-PEKK MCOs (**3**)*

DSC spectrum

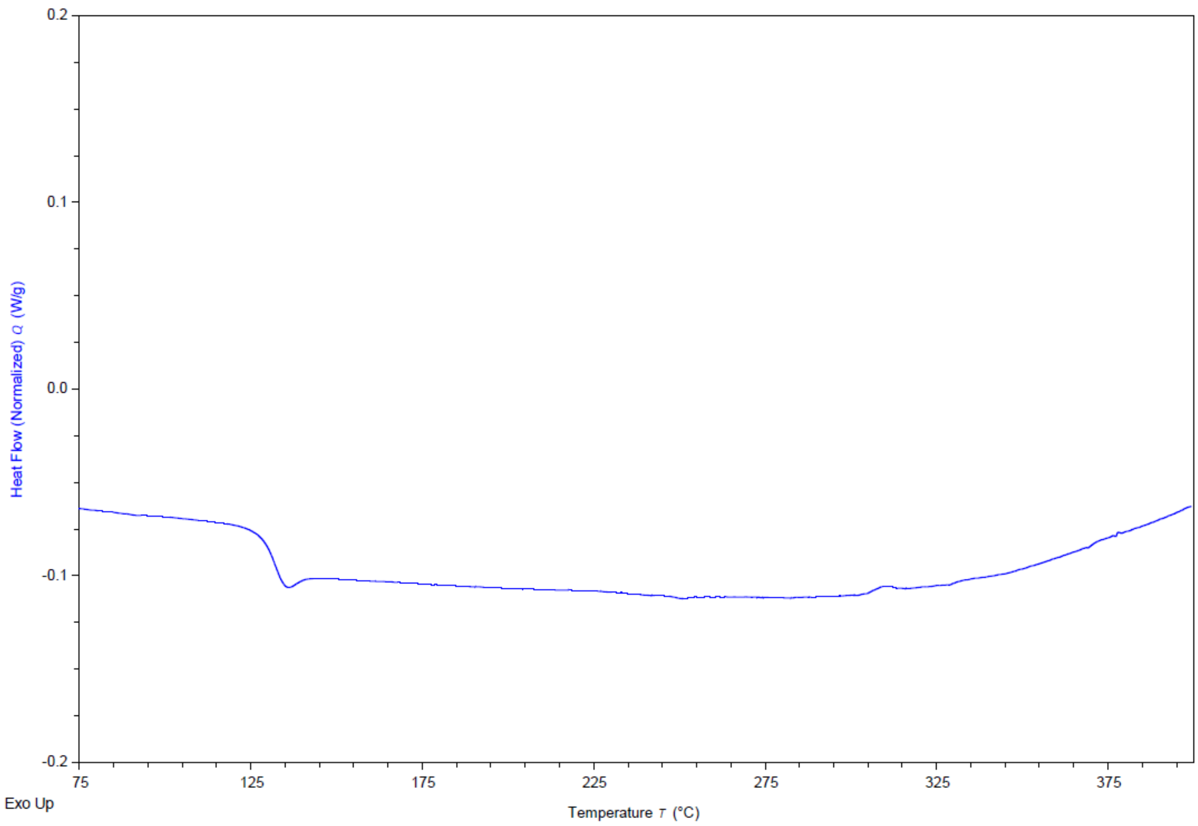

*Figure S15: DSC results of m-PEKK MCOs (**3**) polymerised with 2 mol% CsF initiator, displaying a glass transition at 131 °C*

TGA spectrum of polymerisation

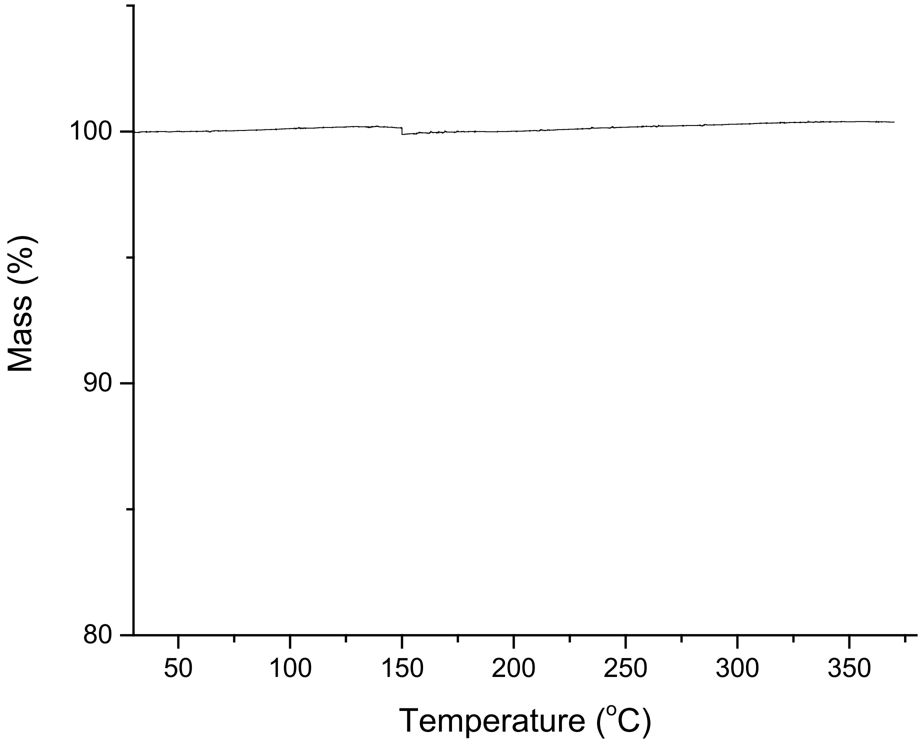

*Figure S16: TGA (heated to 370 °C at 5 °C/min in an alumina crucible and N_2 atmosphere) of the polymerisation of m-PEKK MCOs (**3**) with 2 mol% CsF initiator, demonstrating no significant change in mass during polymerisation.*

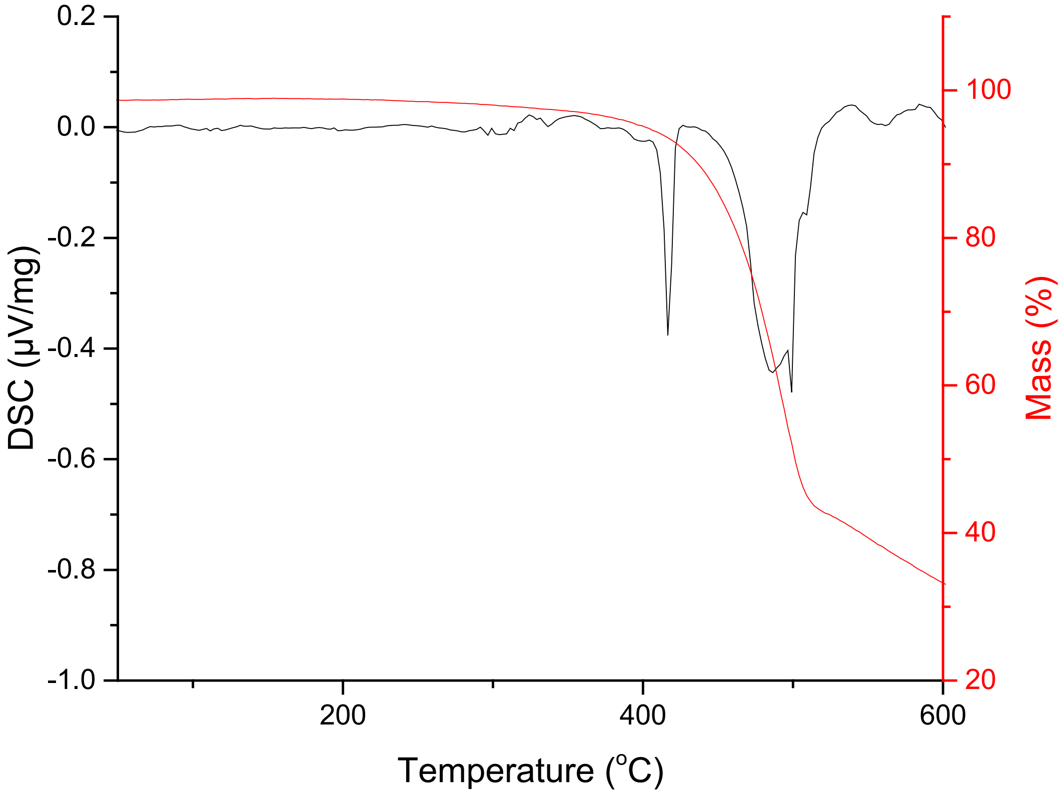

Figure S17: Combined DSC (black line, exo up) and TGA (red line) of purified m-PEKK dimers (heated to 600 °C at 10 °C/min in an alumina crucible and N_2 atmosphere), demonstrating a melting point at 417 °C coinciding with the begin of mass loss due to thermal decomposition.

Characterisation information for 1,4-phenylenebis((4-fluorophenyl)methanone) (4)

NMR (¹H, ¹³C, ¹⁹F) spectra

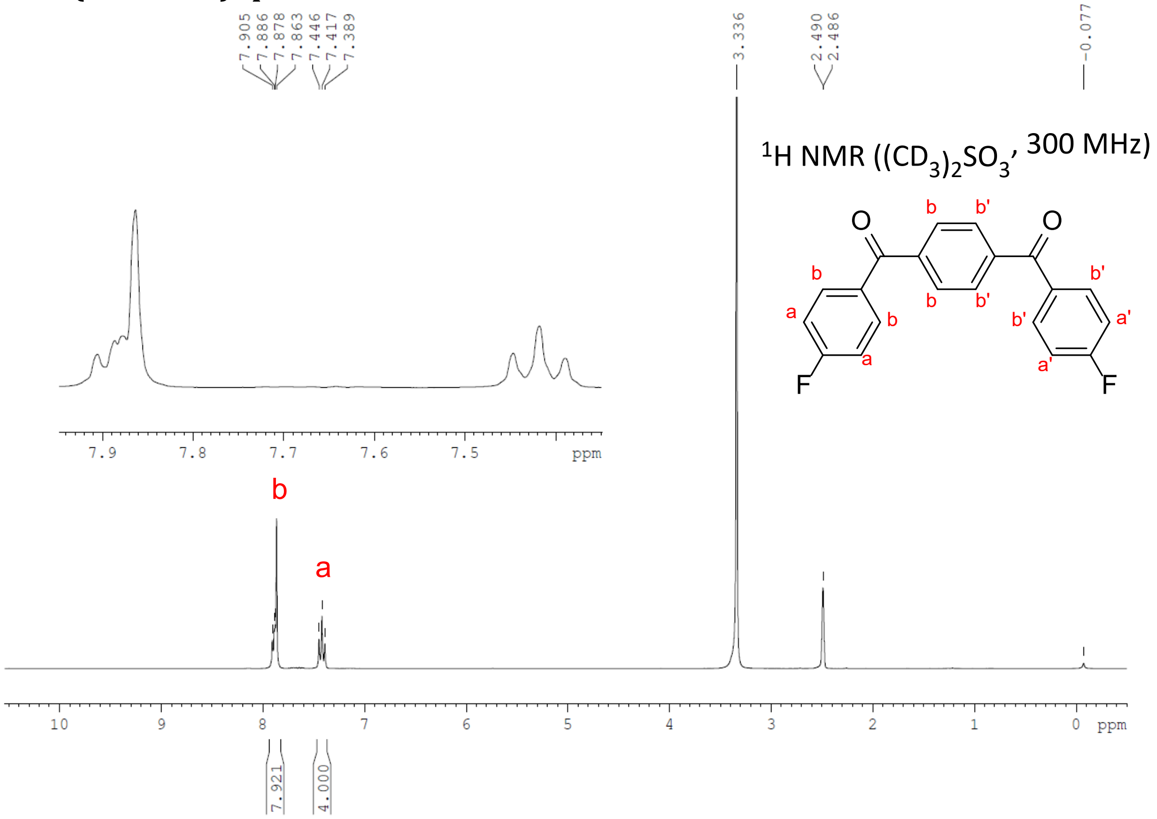

*Figure S18: ^{1}H NMR ($CDCl_3$, 300 MHz) of 1,4-phenylenebis((4-fluorophenyl)methanone) (**4**)*

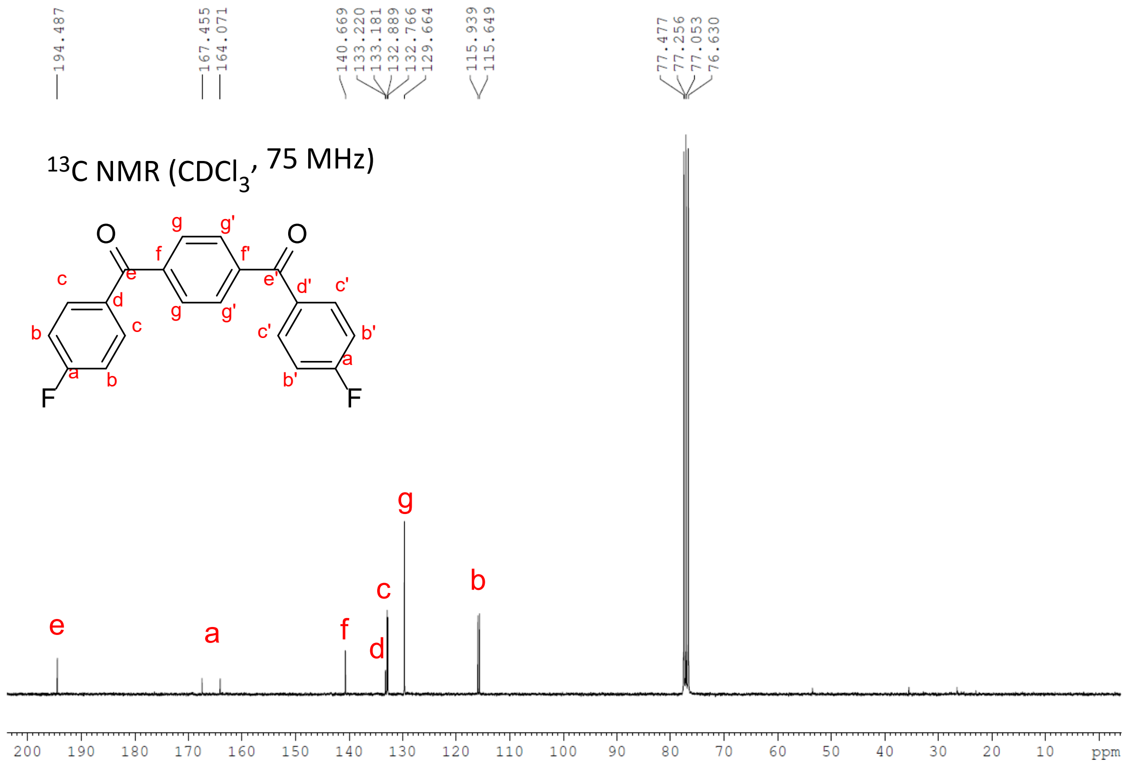

*Figure S19: ^{13}C NMR ($CDCl_3$, 75 MHz) of 1,4-phenylenebis((4-fluorophenyl)methanone) (**4**)*

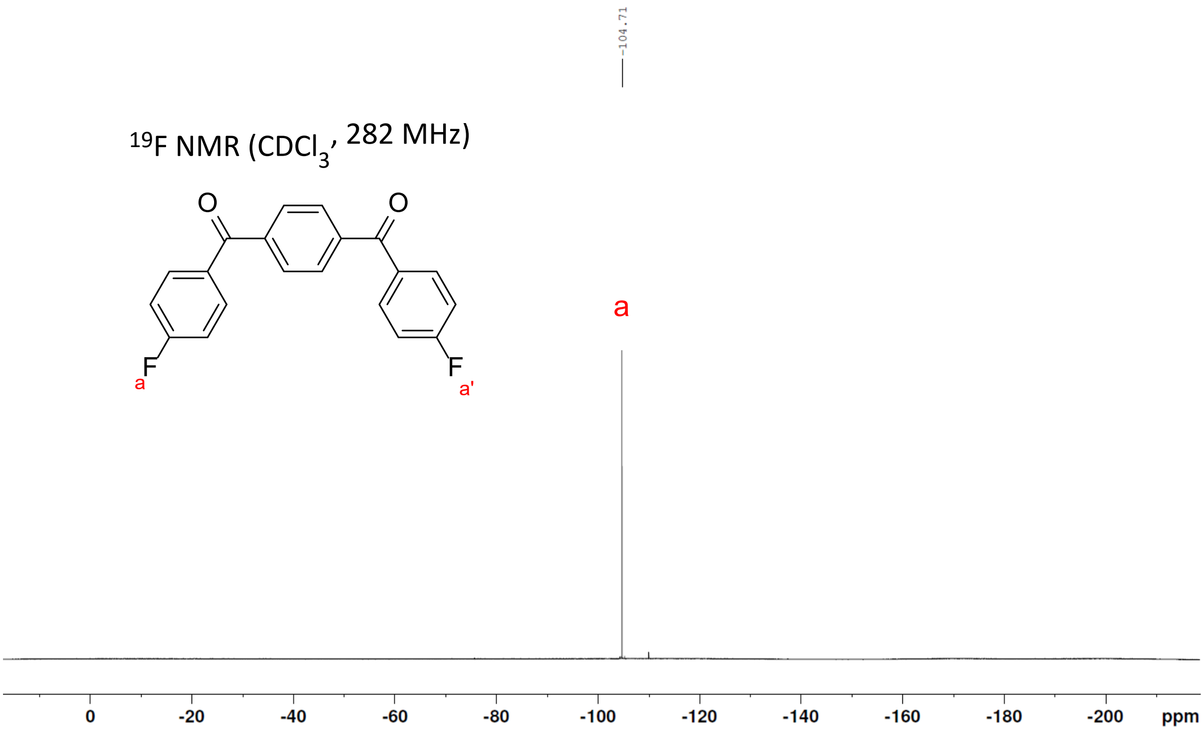

*Figure S20: ^{19}F NMR ($CDCl_3$, 282 MHz) of 1,4-phenylenebis((4-fluorophenyl)methanone) (**4**)*

IR spectrum

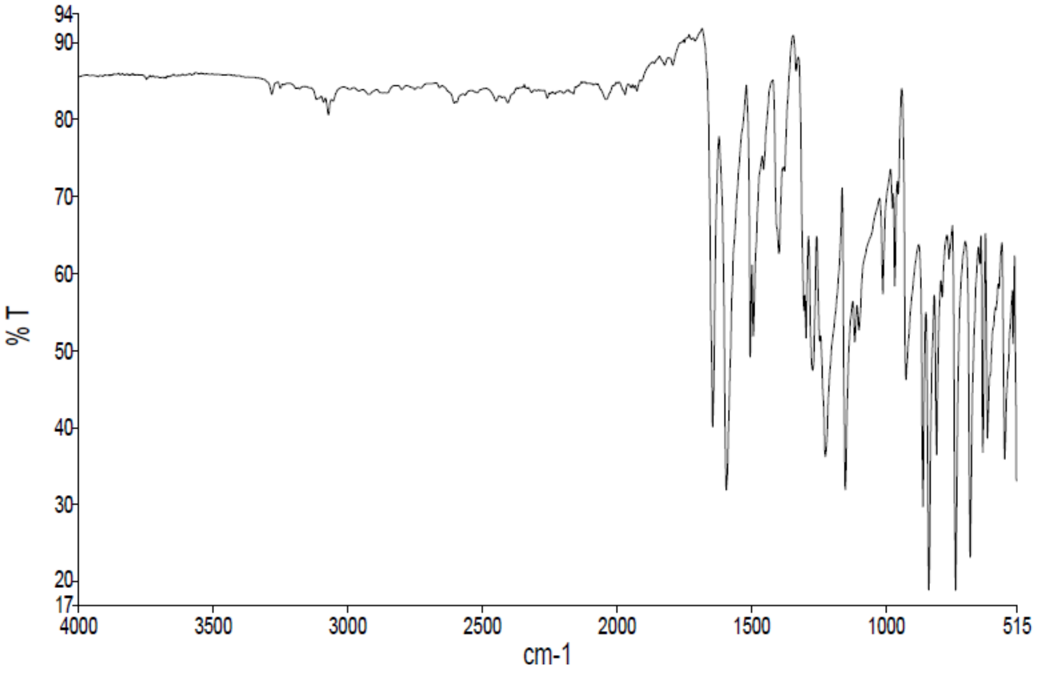

*Figure S21: IR spectrum of 1,4-phenylenebis((4-fluorophenyl)methanone) (**4**)*

Characterisation information for 1,4-phenylenebis((4-hydroxyphenyl)methanone) (5)

NMR (^{1}H, ^{13}C, ^{19}F) spectra

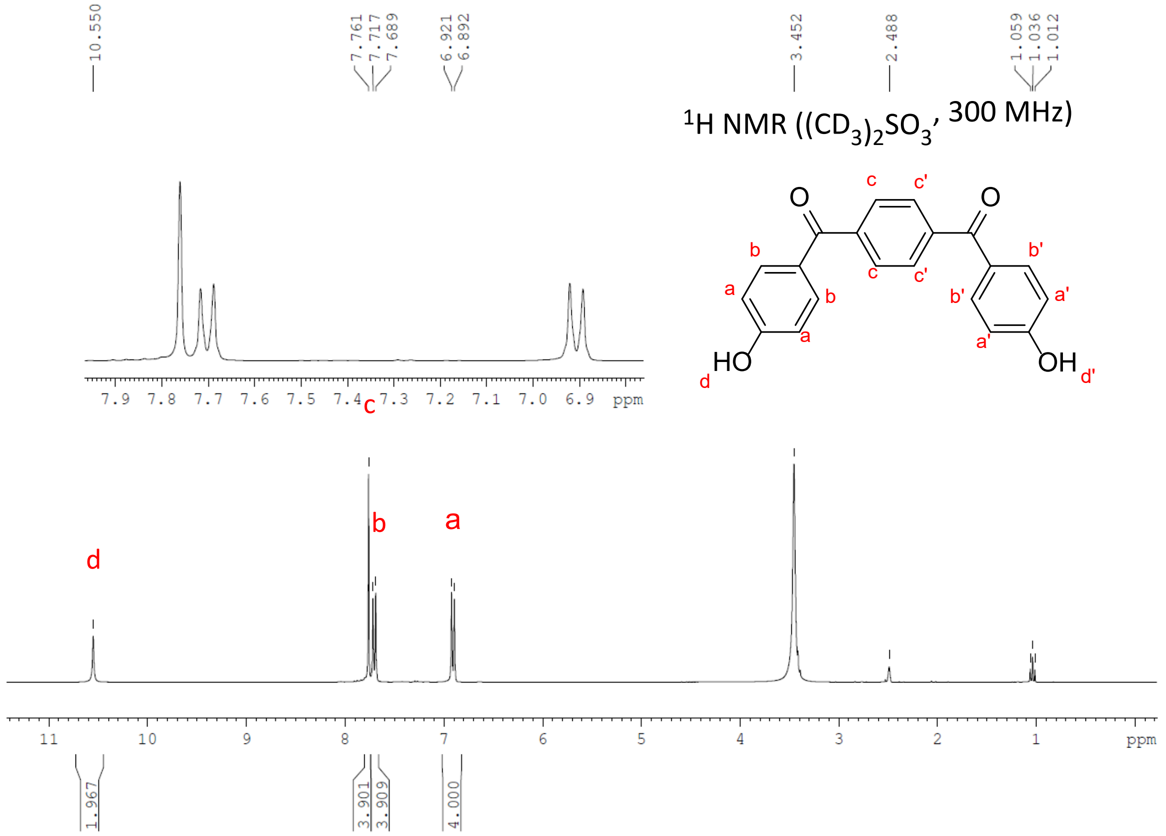

*Figure S22: ^{1}H NMR ($(CD_3)_2SO_3$, 300 MHz) of 1,4-phenylenebis((4-hydroxyphenyl)methanone) (**5**)*

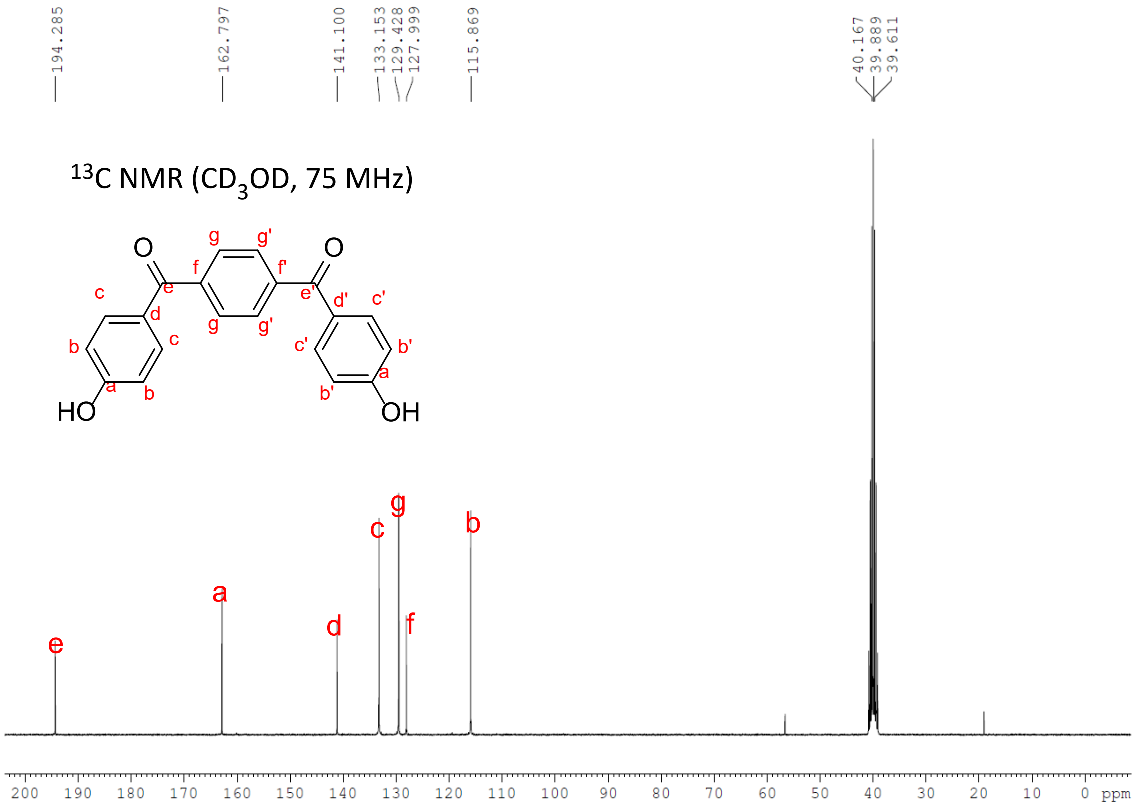

*Figure S23: ^{13}C NMR (CD_3OD, 75 MHz) of 1,4-phenylenebis((4-hydroxyphenyl)methanone) (**5**)*

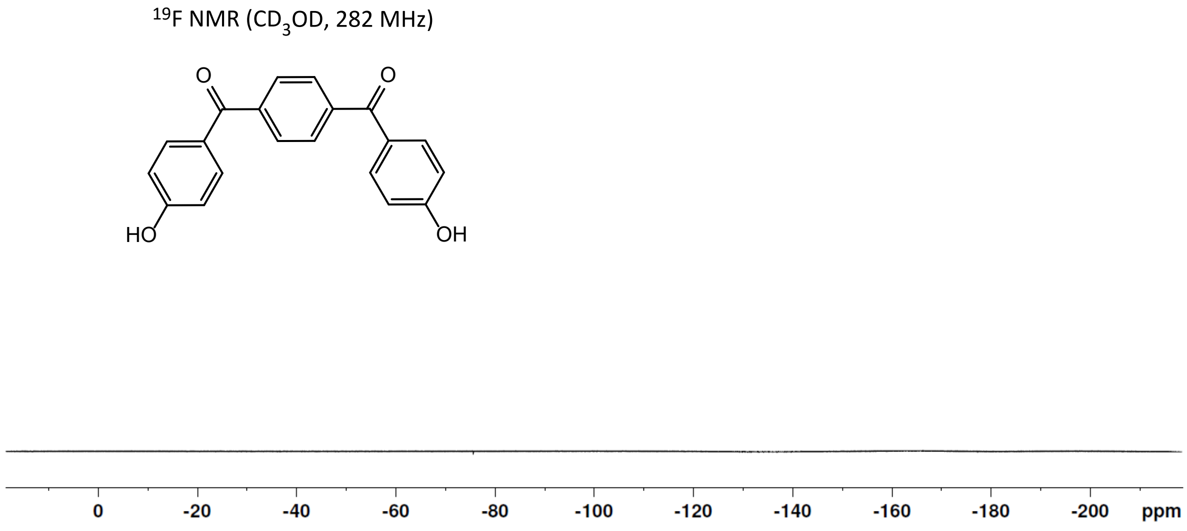

*Figure S24: ^{19}F NMR (CD_3OD, 282 MHz) of 1,4-phenylenebis((4-hydroxyphenyl)methanone) (**5**)*

IR spectrum

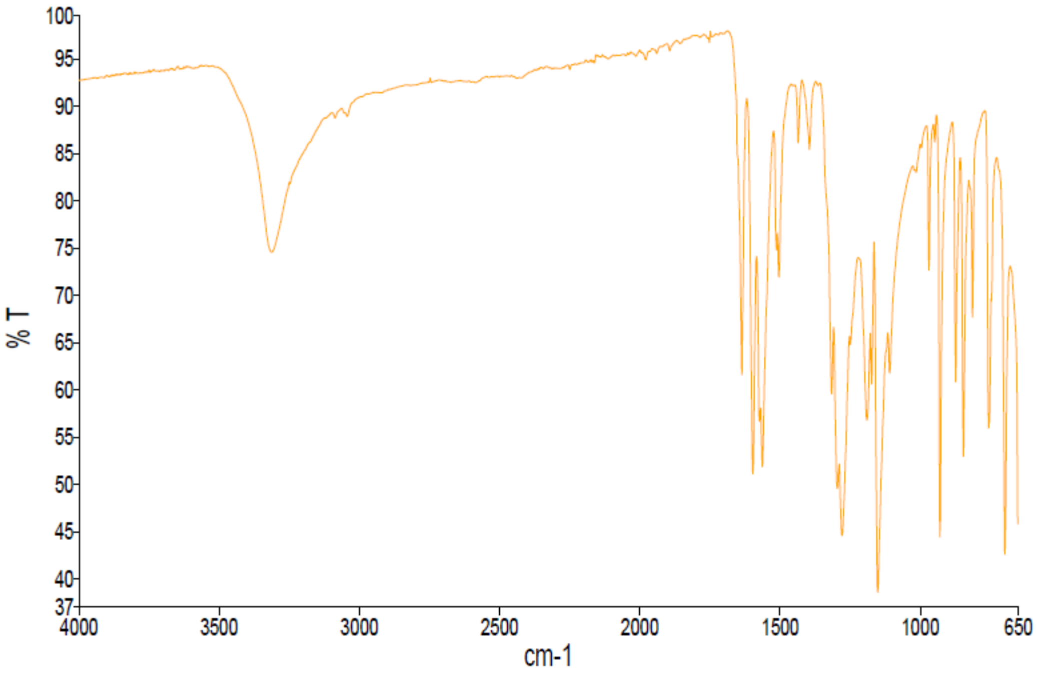

*Figure S25: IR spectrum of 1,4-phenylenebis((4-hydroxyphenyl)methanone) (**5**)*

Characterisation information for p-PEKK MCOs (6) and their thermal properties

NMR (^{1}H, ^{13}C, HMBC) spectra

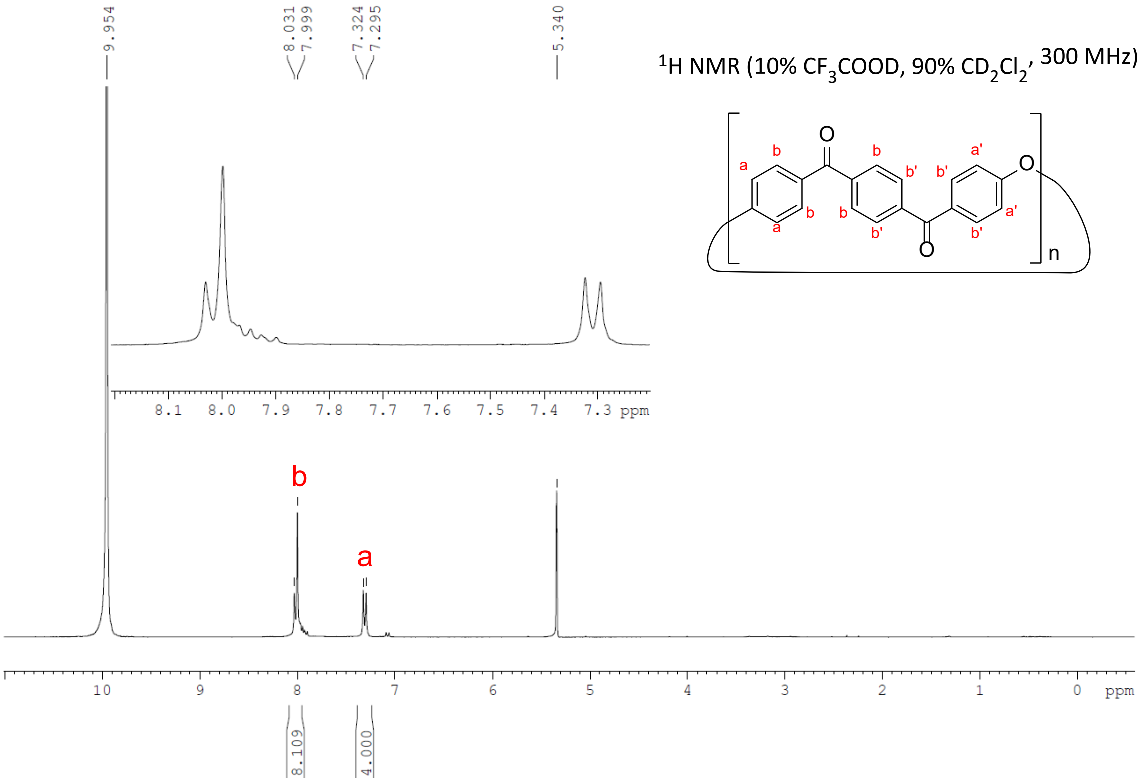

*Figure S26: ^{1}H NMR (10% CF_3COOD, 90% CD_2Cl_2, 300 MHz) of p-PEKK MCOs (**6**)*

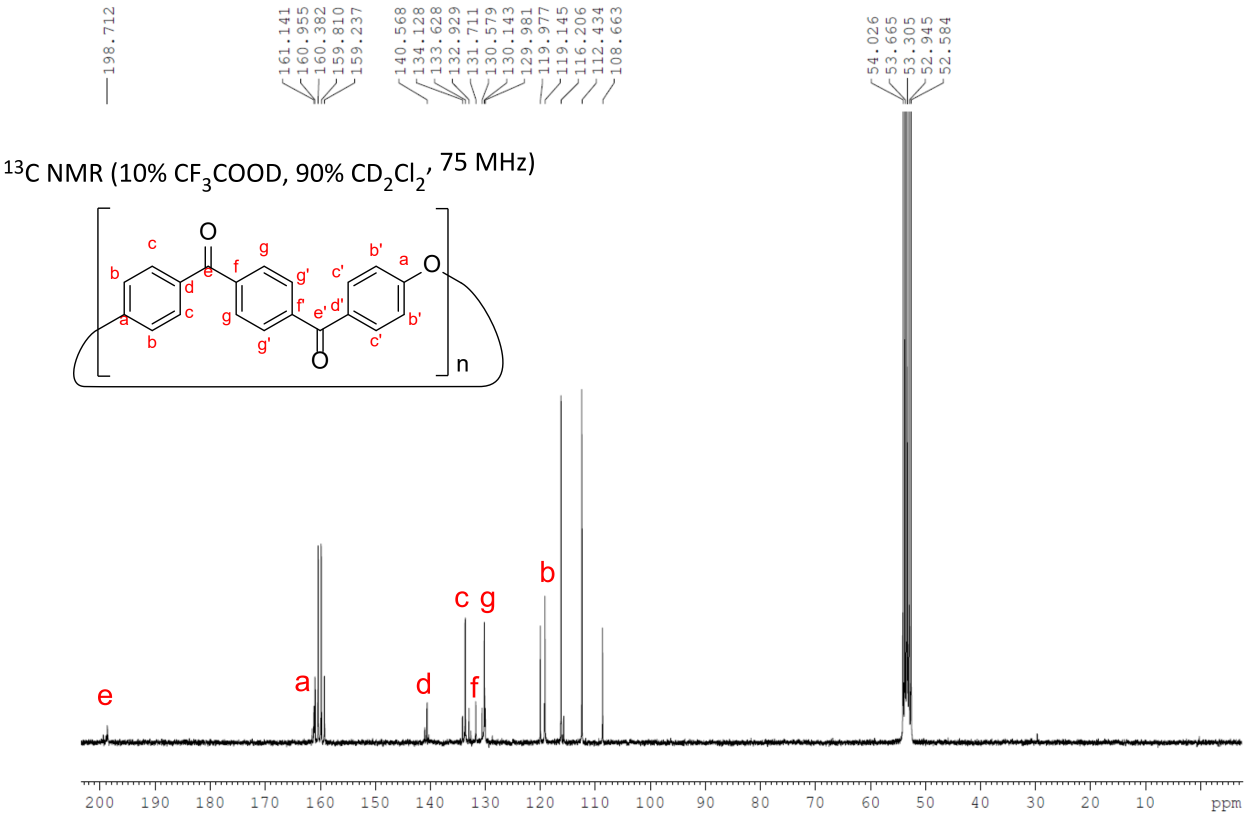

*Figure S27: ^{13}C NMR (10% CF_3COOD, 90% CD_2Cl_2, 75 MHz) of p-PEKK MCOs (**6**)*

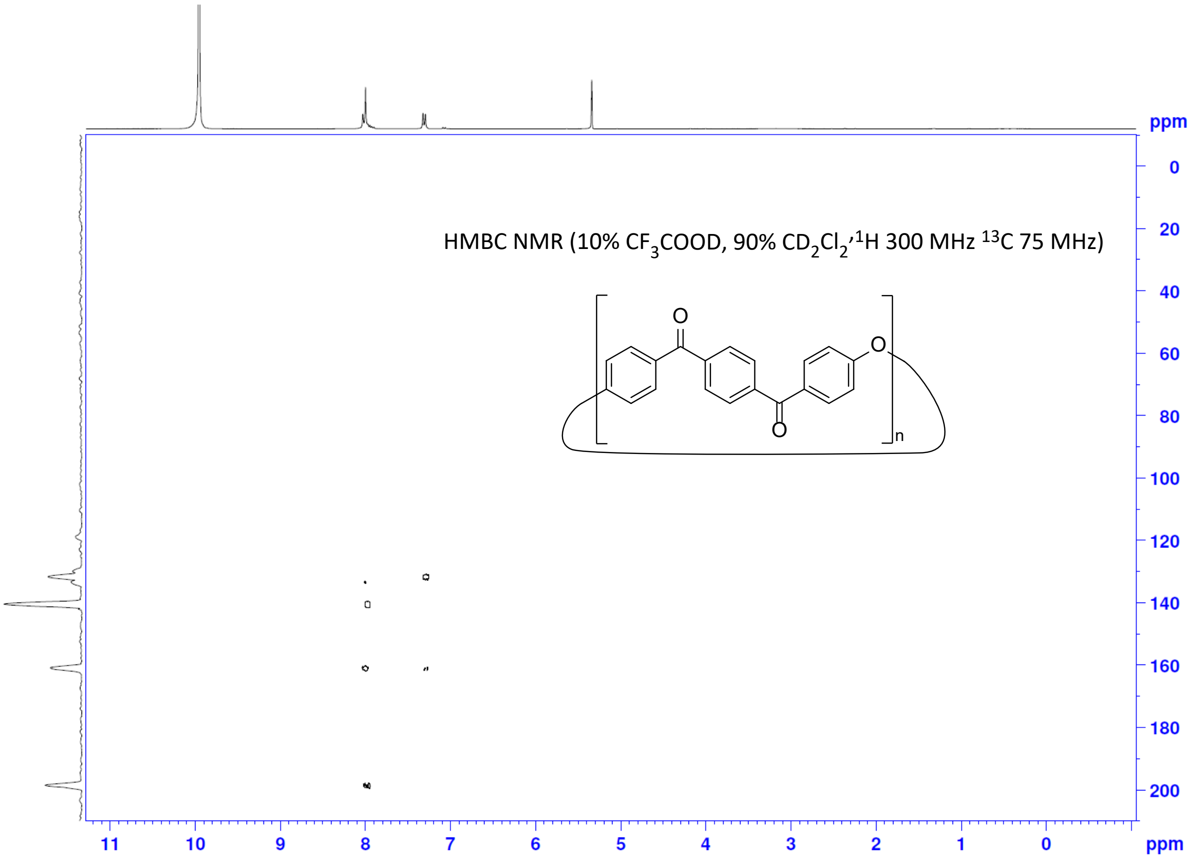

*Figure S28: HMBC NMR (10% CF_3COOD, 90% CD_2Cl_2, ^1H 300 MHz ^{13}C 75 MHz) of p-PEKK MCOs (**6**)*

MALDI-ToF spectrum

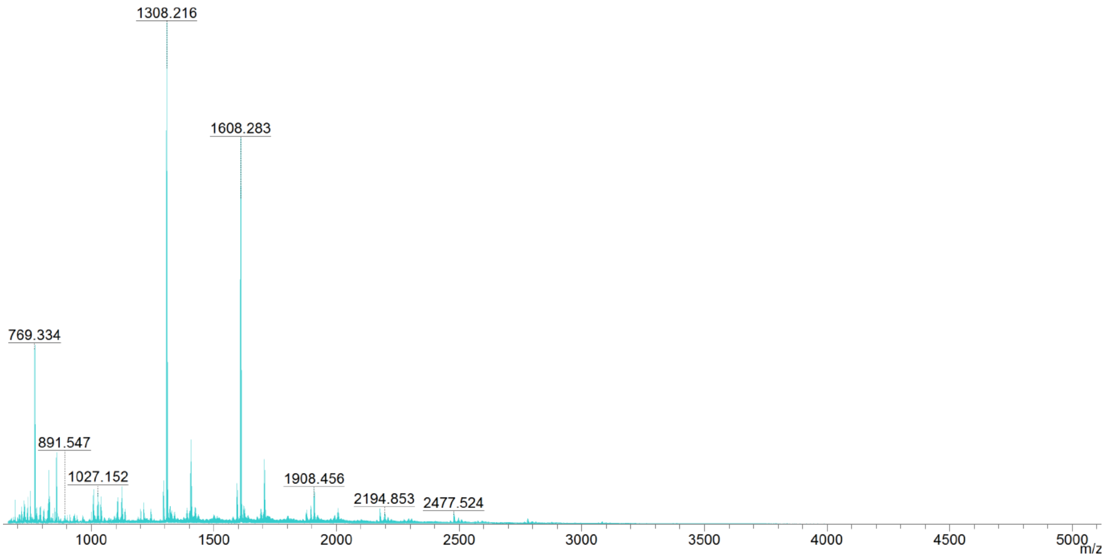

*Figure S29: MALDI-ToF spectrum of p-PEKK MCOs (**6**) with silver trifluoroacetate cationising agent. [M(cyclic monomer unit] Calcd for $C_{20}H_{12}O_3$=300.08 m/z. $[M(tetramer)+Ag]^+$ Calcd for $C_{80}H_{48}O_{12}Ag$ =1307.22 m/z, found 1308.22 m/z*

IR spectrum

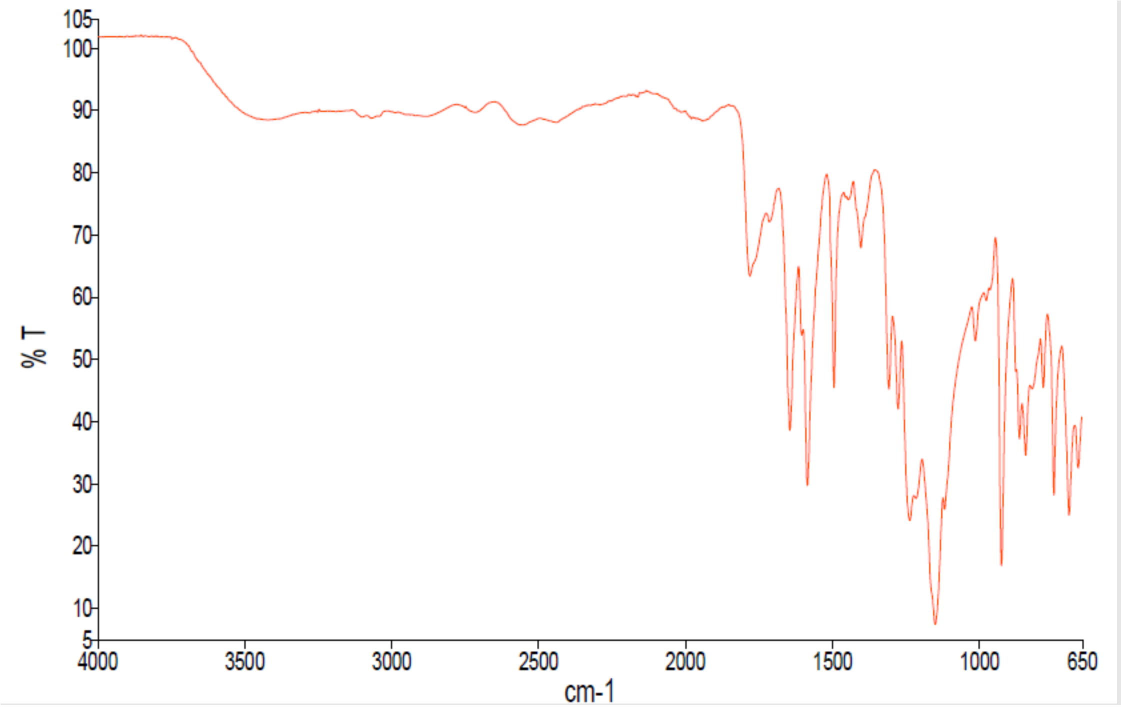

*Figure S30: IR spectrum of p-PEKK MCOs (**6**)*

GPC chromatogram

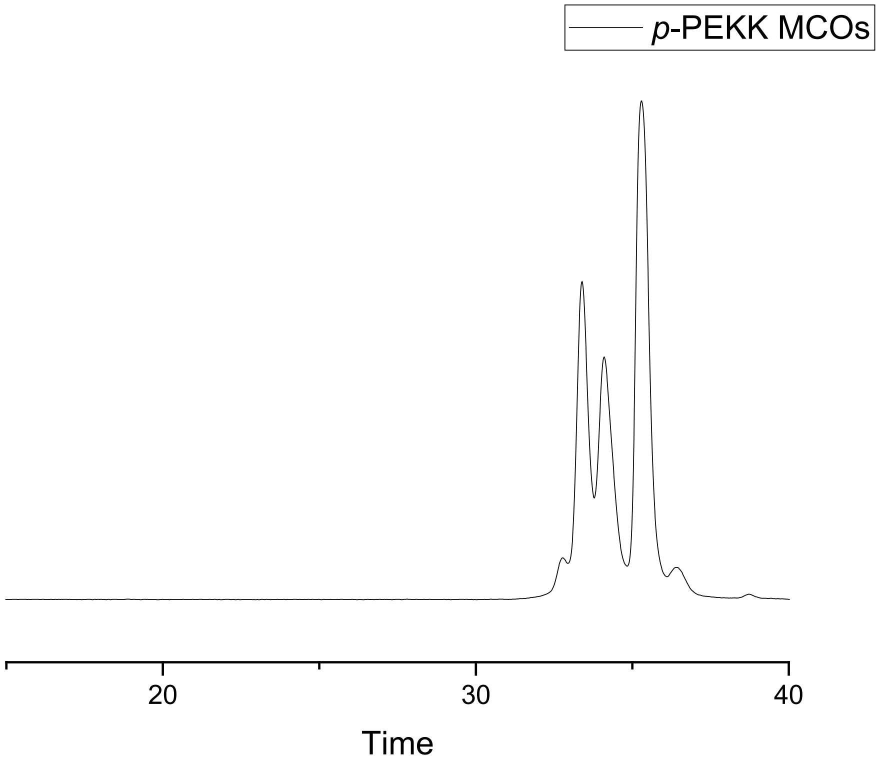

*Figure S 31: GPC chromatogram of p-PEKK MCOs (**6**)*

DSC spectrum

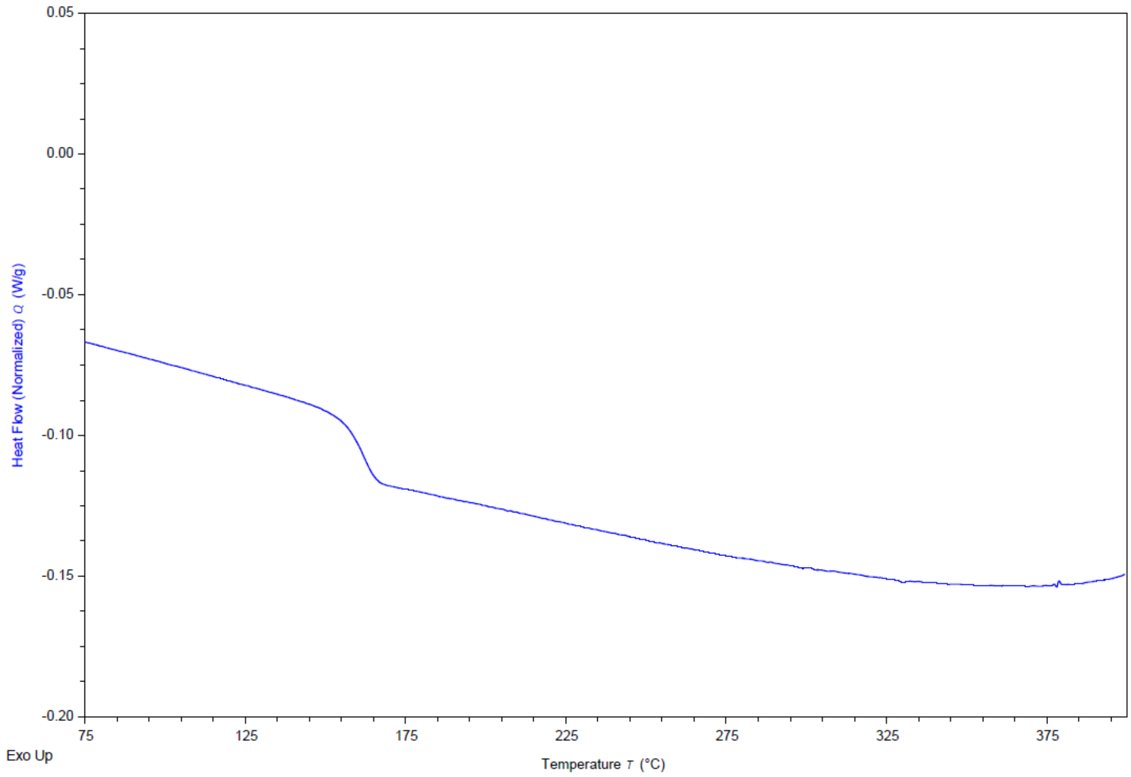

*Figure S32: DSC results of p-PEKK MCOs (**6**) polymerised with 2 mol% CsF initiator, displaying a glass transition at 162 °C*

Characterisation information for 60/40 T/I MCOs and their thermal properties

NMR (^{1}H, ^{13}C, HMBC) spectra

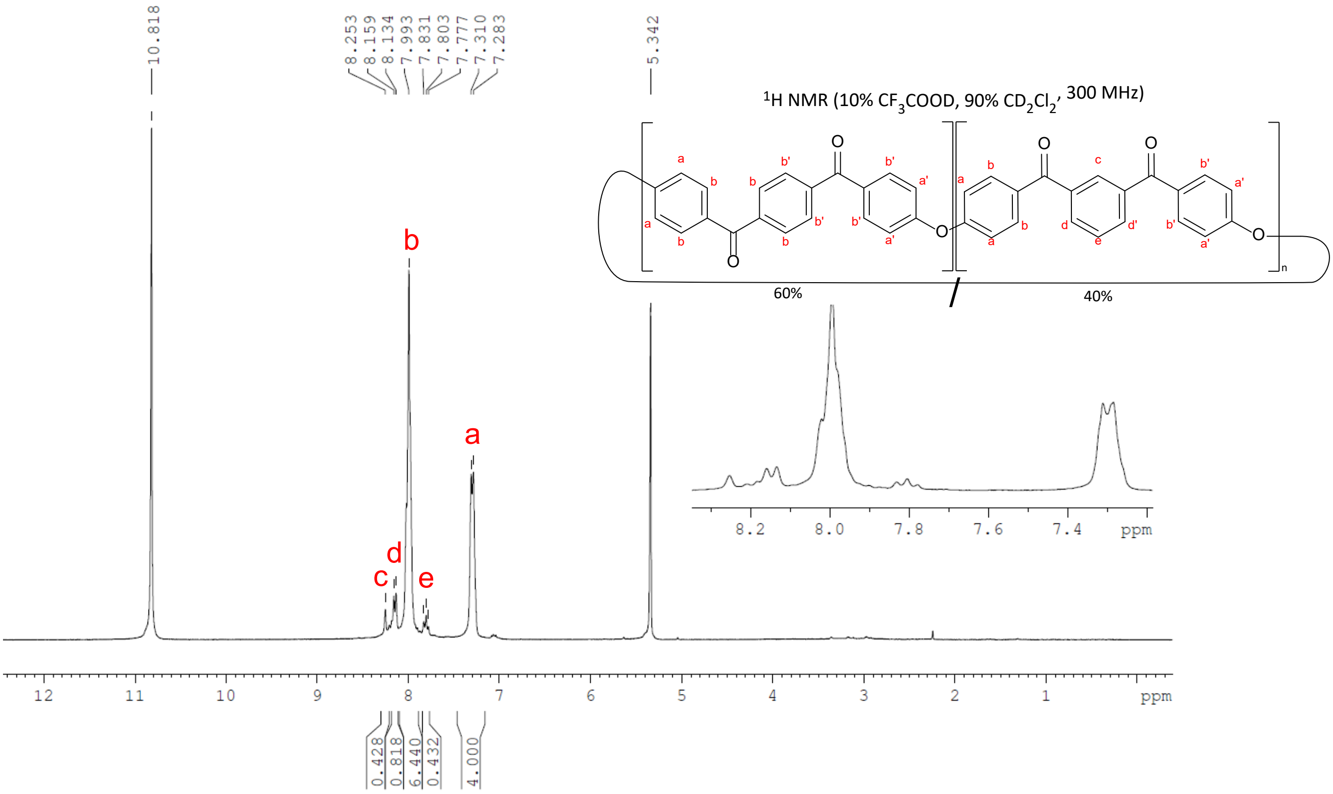

Figure S33: ^{1}H NMR (10% CF_3COOD, 90% CD_2Cl_2, 300 MHz) of 60/40 T/I MCOs

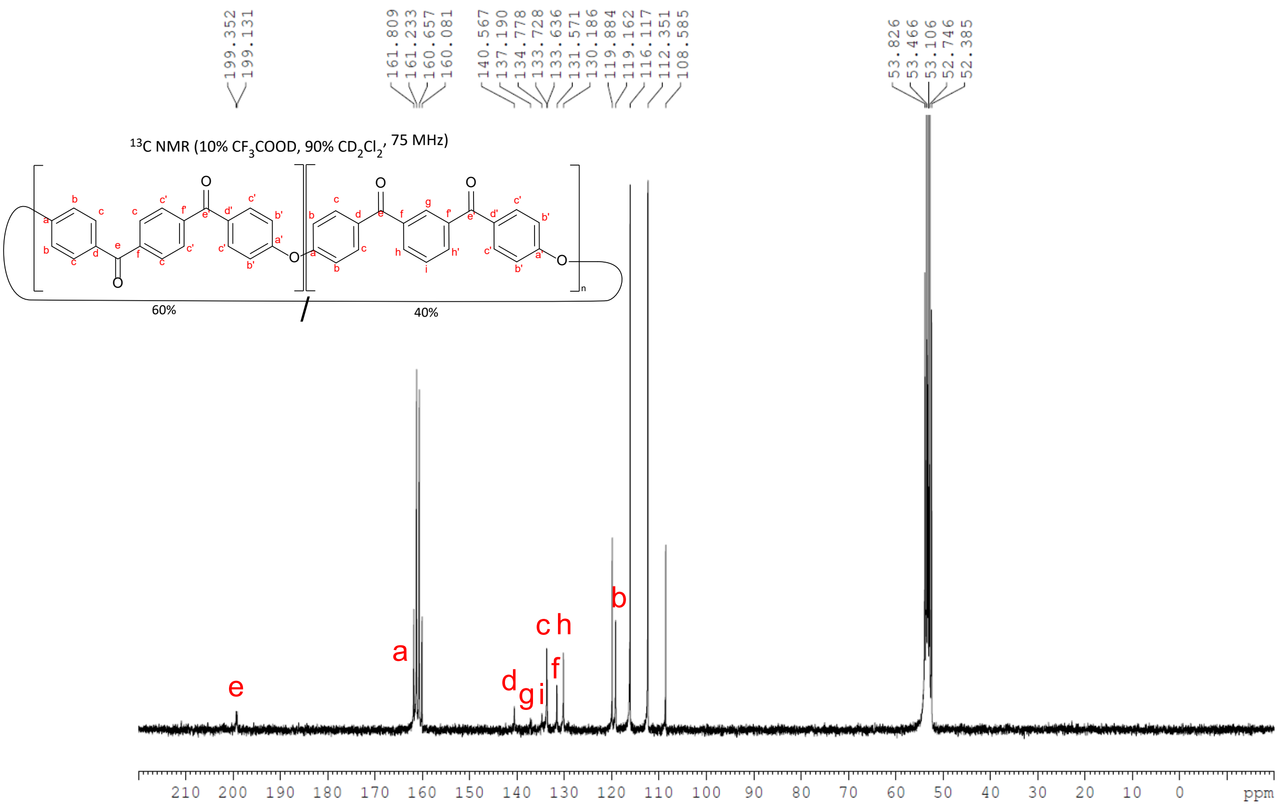

Figure S34: ^{13}C NMR (10% CF_3COOD, 90% CD_2Cl_2, 75 MHz) of 60/40 T/I PEKK MCOs

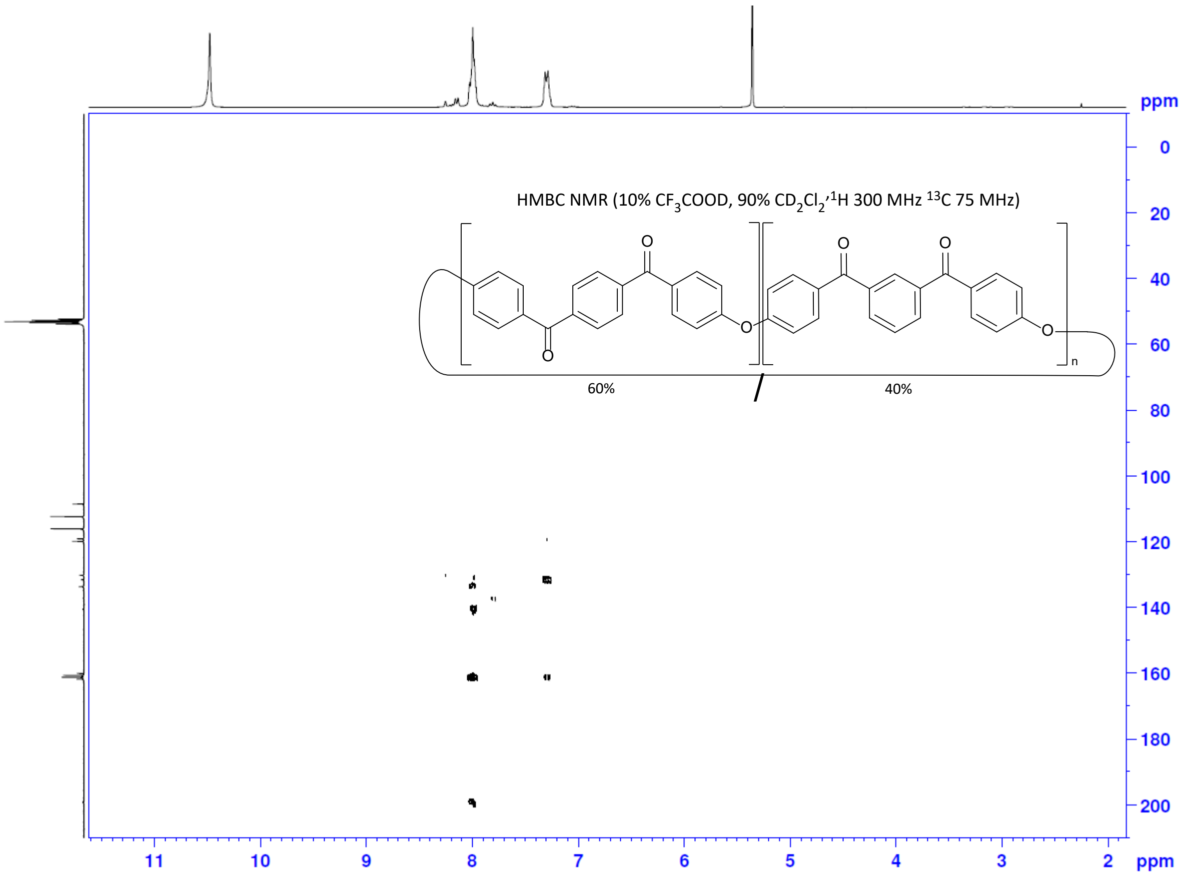

Figure S35: HMBC NMR (10% CF_3COOD, 90% CD_2Cl_2, ^1H 300 MHz ^{13}C 75 MHz) of 60/40 T/I PEKK MCOs

MALDI-ToF spectrum

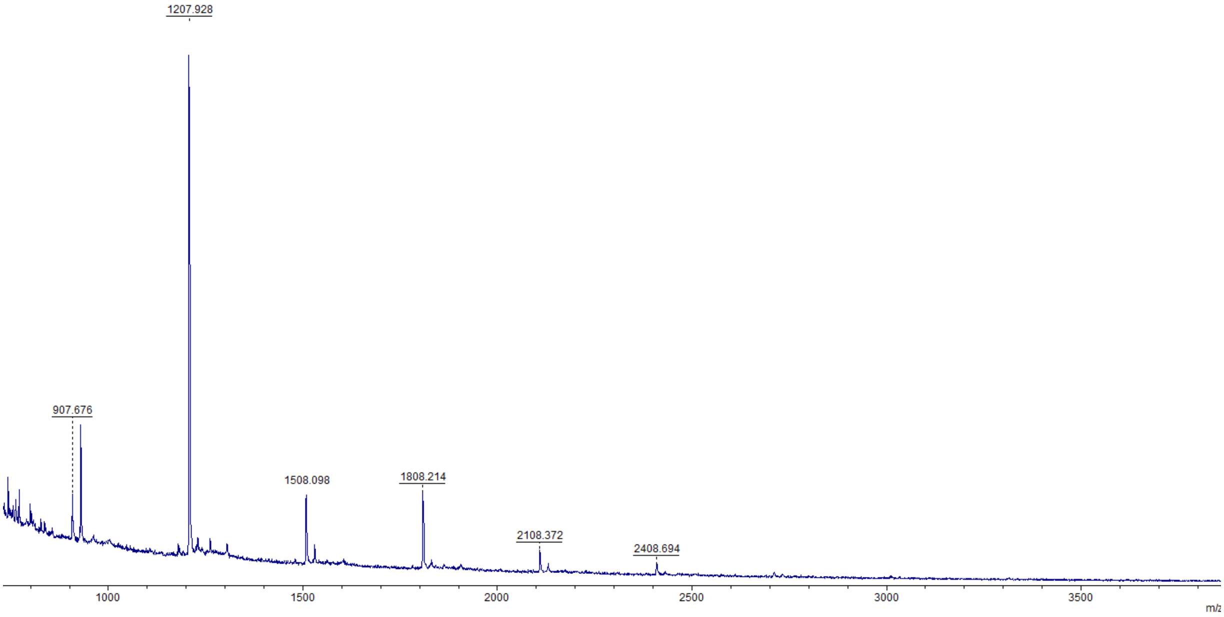

Figure S36: MALDI-ToF spectrum of 60/40 T/I PEKK MCOs with LiBr cationising agent. [M(cyclic monomer unit] Calcd for $C_{20}H_{12}O_3$=300.08 m/z. $[M(tetramer)+Li]^+$ Calcd for $C_{80}H_{48}O_{12}Li$ =1207.33 m/z, found 1207.93 m/z

Characterisation information for 70/30 T/I MCOs and their thermal properties

NMR (^{1}H, ^{13}C) spectra

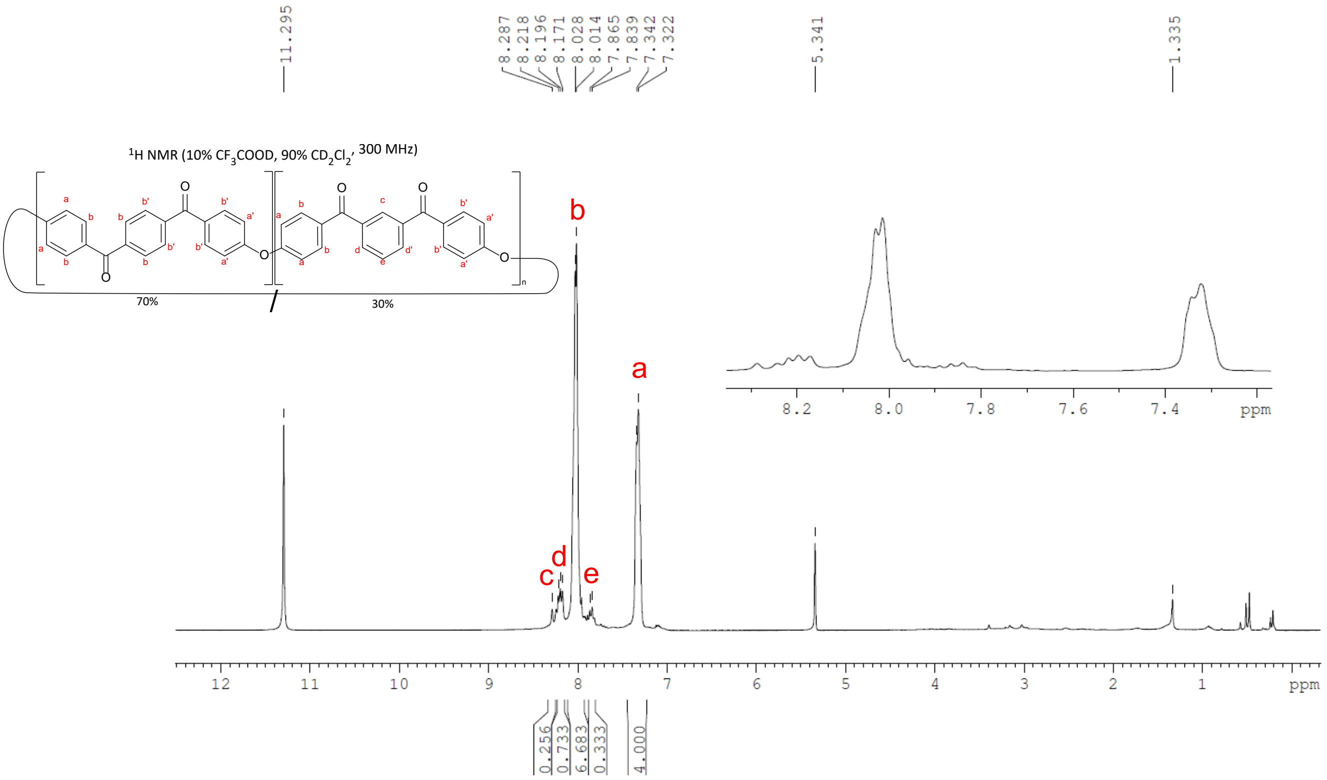

Figure S37: ^{1}H NMR (10% CF_3COOD, 90% CD_2Cl_2, 300 MHz) of 70/30 T/I MCOs

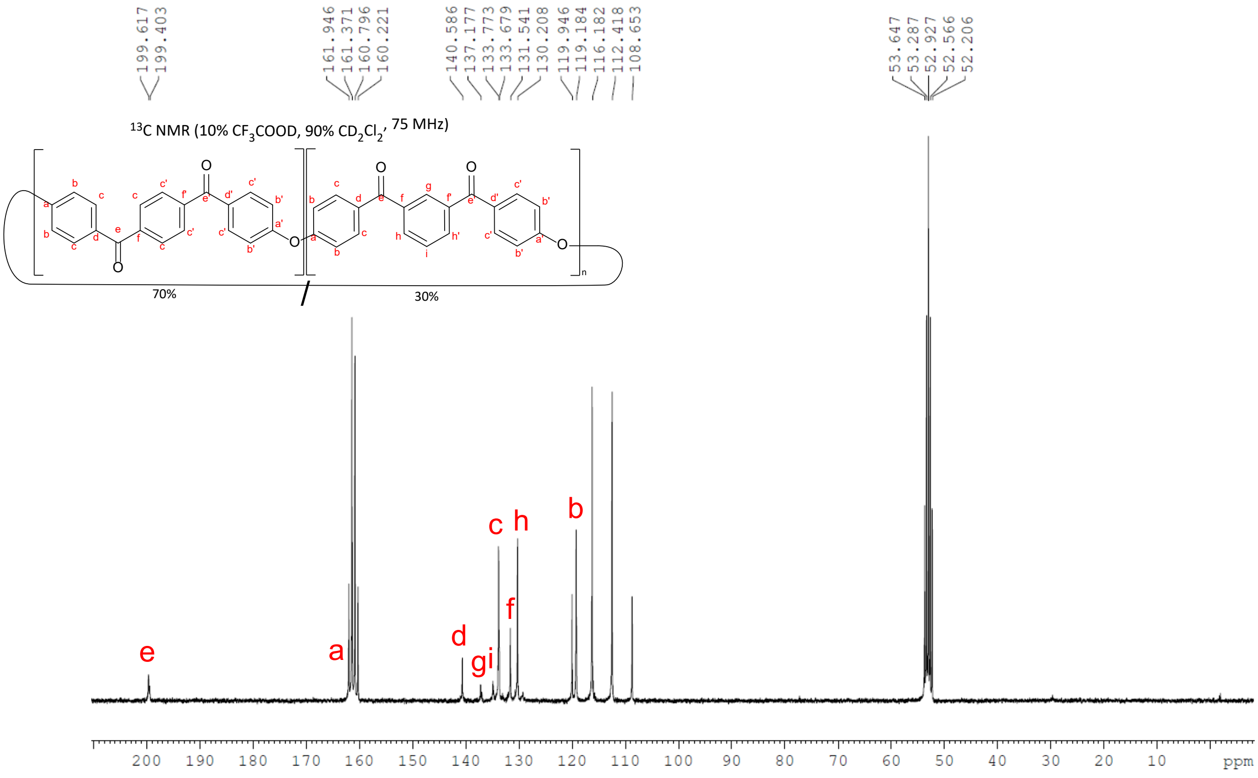

Figure S38: ^{13}C NMR (10% CF_3COOD, 90% CD_2Cl_2, 75 MHz) of 70/30 T/I PEKK MCOs

MALDI-ToF spectrum

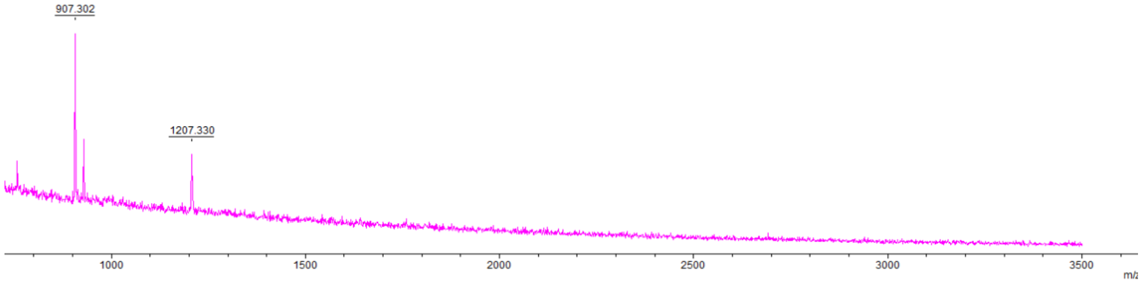

Figure S39: MALDI-ToF spectrum of 70/30 T/I PEKK MCOs with LiBr cationising agent. [M(cyclic monomer unit] Calcd for $C_{20}H_{12}O_3$=300.08 m/z. [M(tetramer)+Li]$^+$ Calcd for $C_{80}H_{48}O_{12}Li$ =1207.33 m/z, found 1207.33 m/z

DSC spectrum

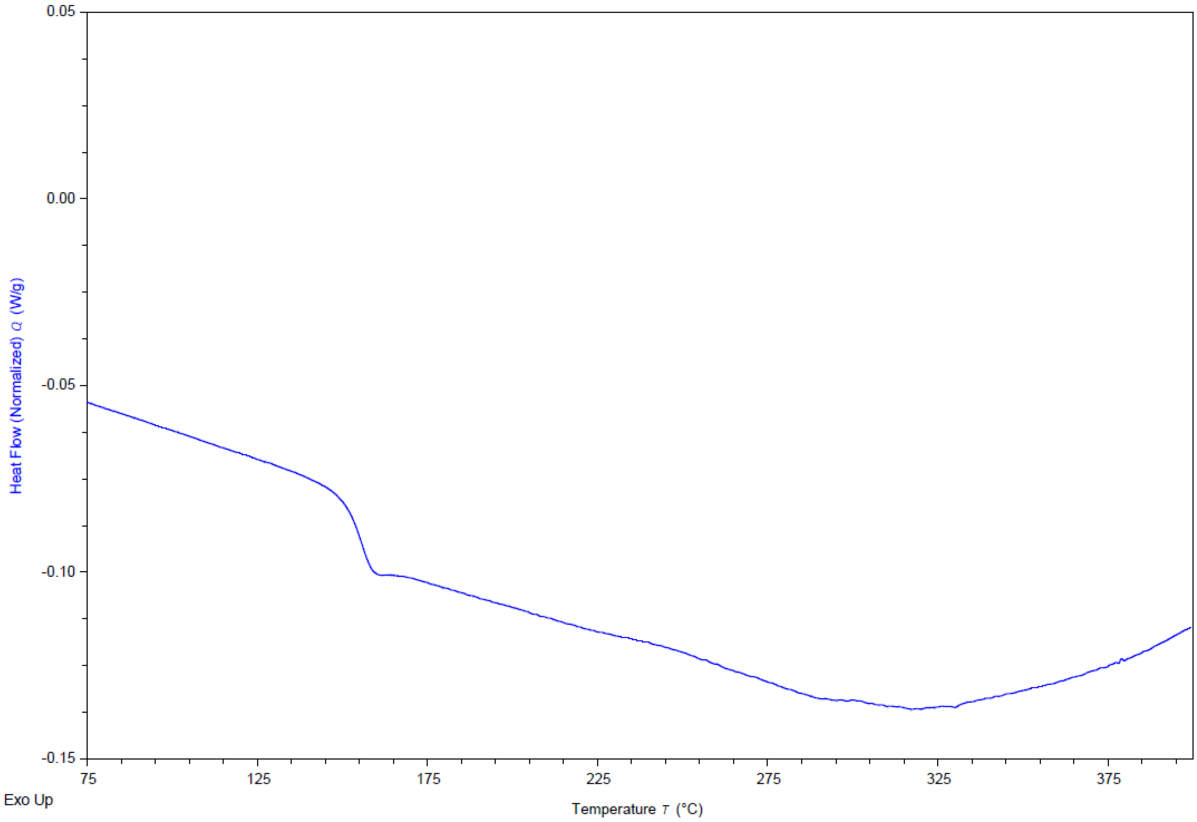

Figure S 40: DSC results of 70/30 T/I PEKK MCOs polymerised with 2 mol% CsF initiator, displaying a glass transition at 155 °C

Characterisation information for 80/20 T/I MCOs and their thermal properties

NMR (^{1}H, ^{13}C) spectra

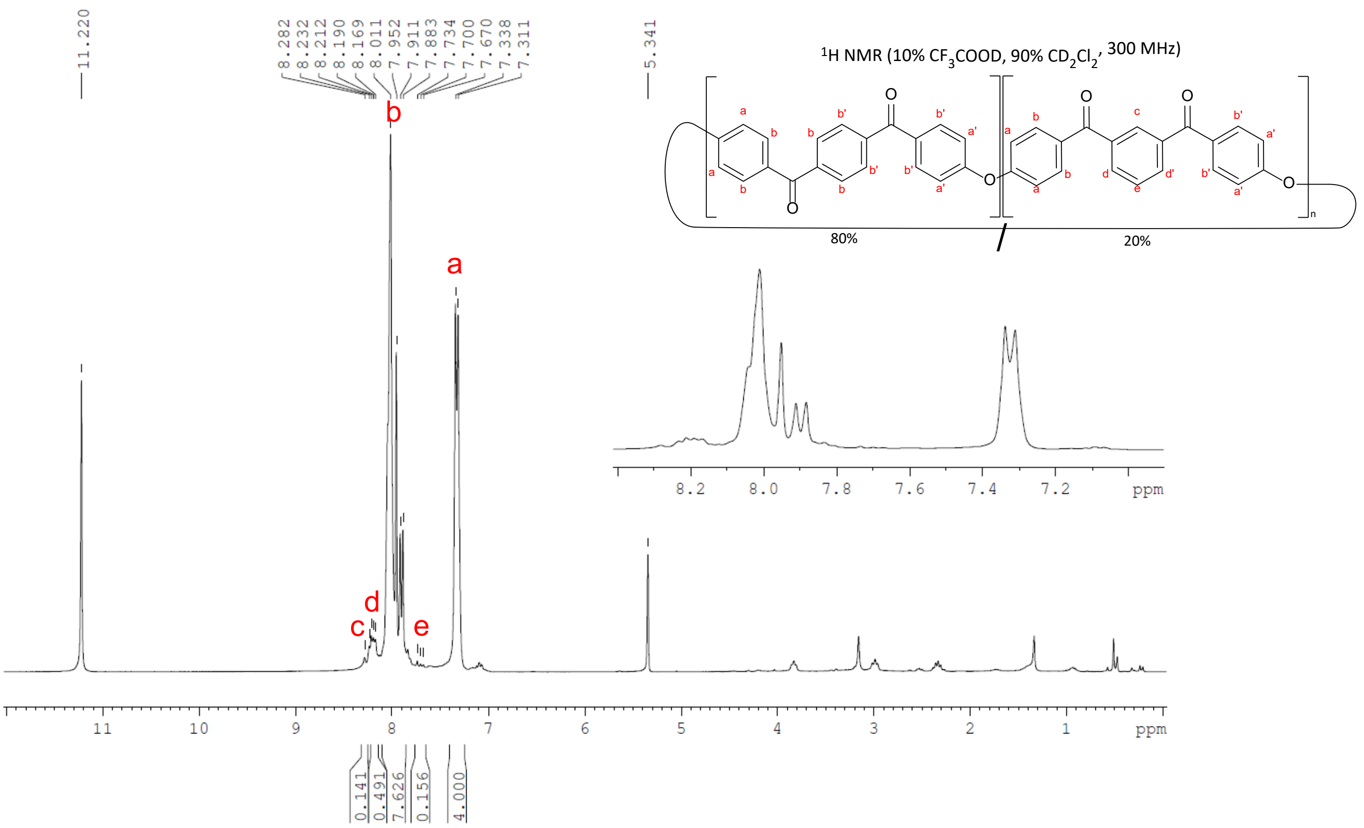

Figure S41: ^{1}H NMR (10% CF_3COOD, 90% CD_2Cl_2, 300MHz) of 80/20 T/I MCOs

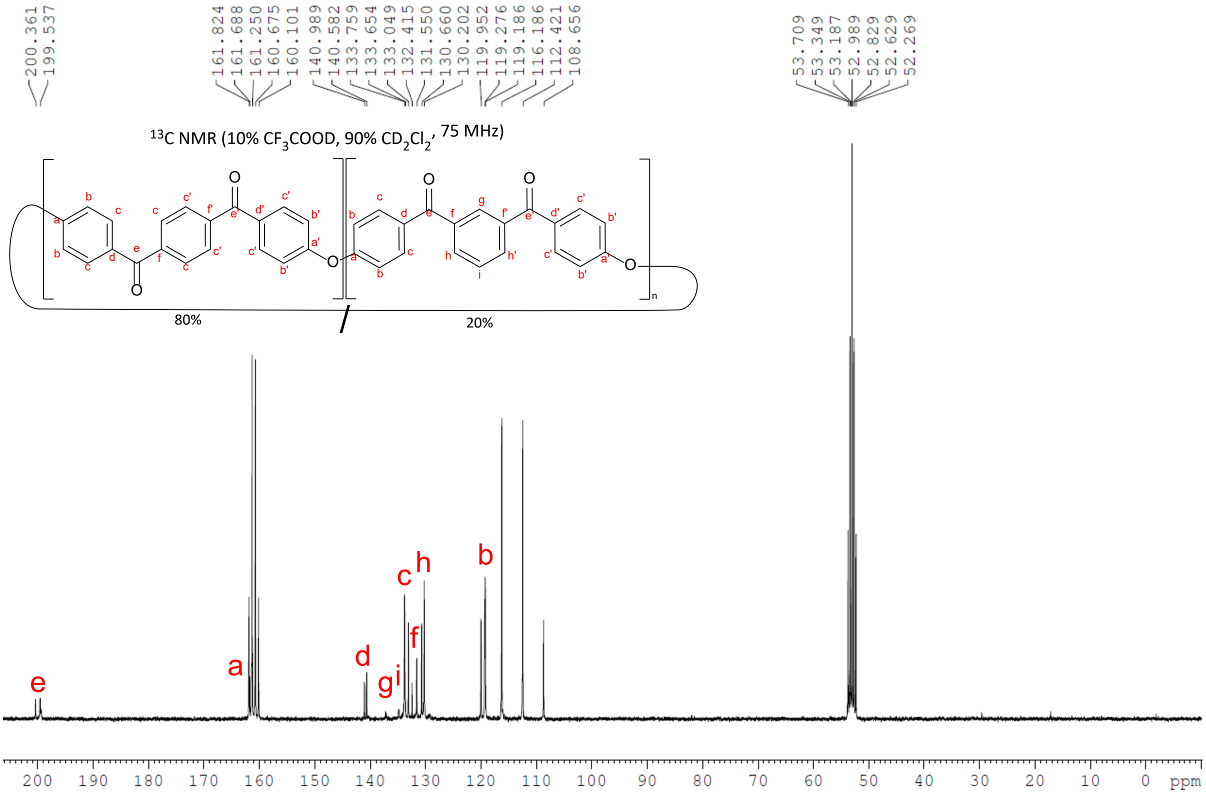

Figure S42: ^{13}C NMR (10% CF_3COOD, 90% CD_2Cl_2, 75 MHz) of 80/20 T/I PEKK MCOs

MALDI-ToF spectrum

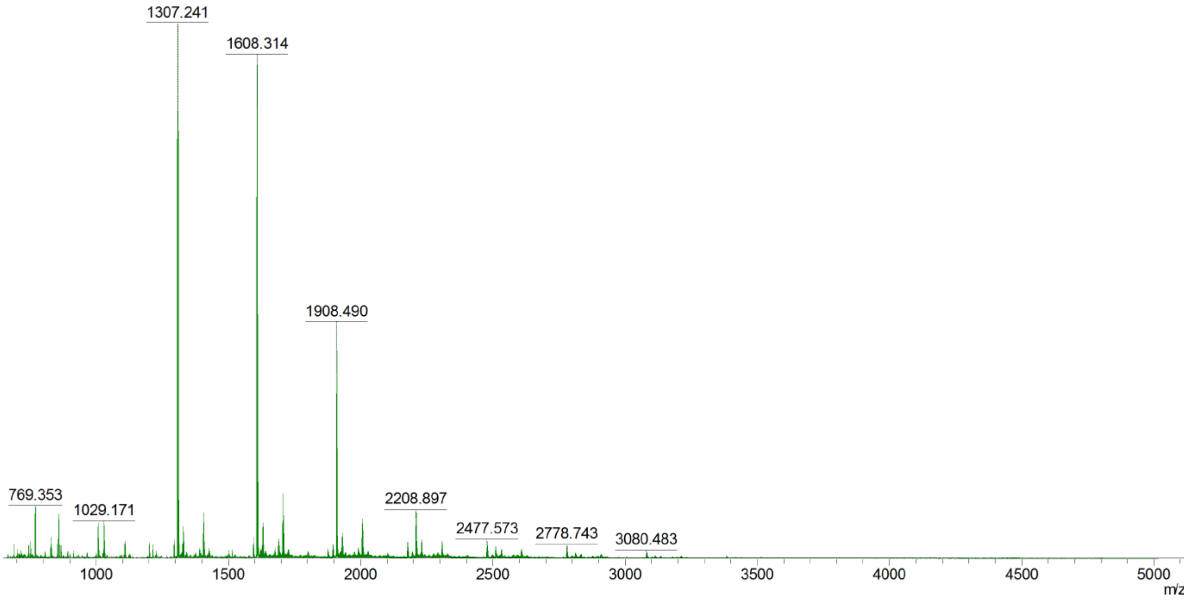

Figure S43: MALDI-ToF spectrum of 80/20 T/I PEKK MCOs with silver trifluoroacetate cationising agent. [M(cyclic monomer unit] Calcd for $C_{20}H_{12}O_3$=300.08 m/z. $[M(tetramer)+Ag]^+$ Calcd for $C_{80}H_{48}O_{12}Ag$ =1307.22 m/z, found 1307.24 m/z

DSC spectrum

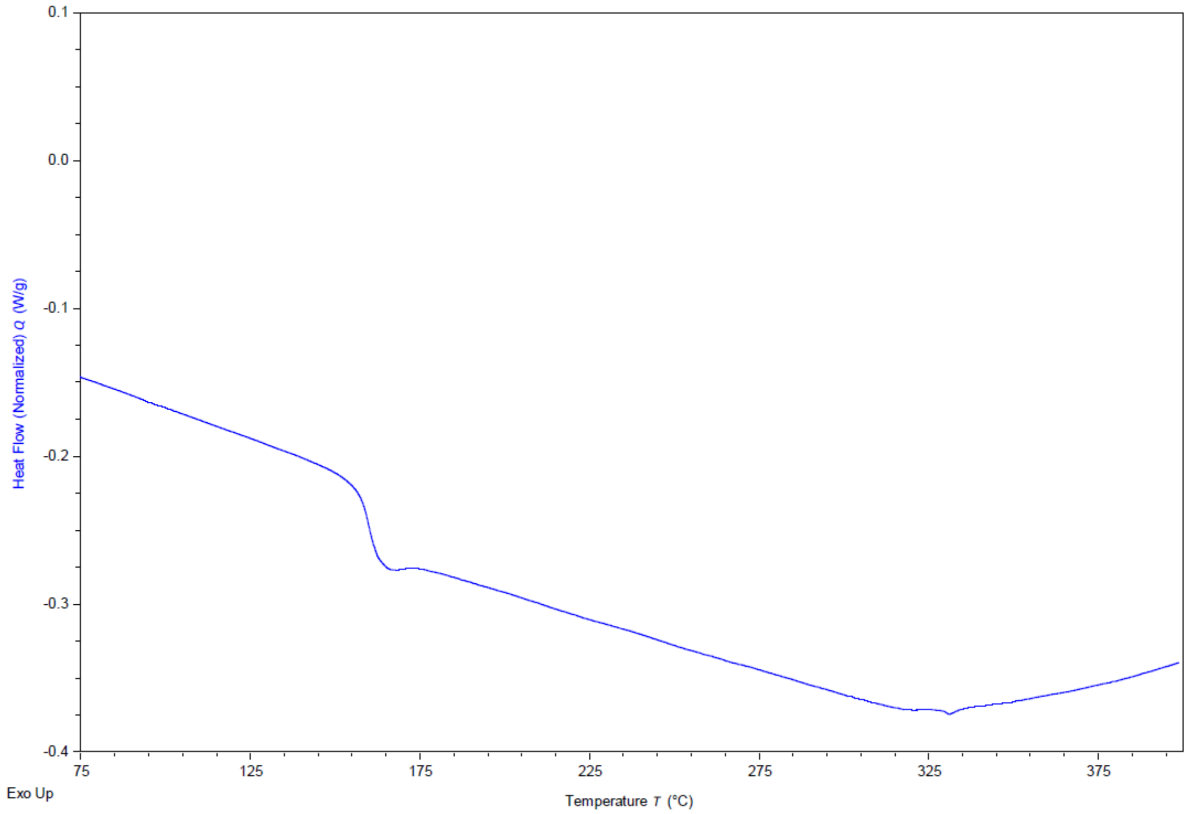

Figure S44: DSC results of 80/20 T/I PEKK MCOs polymerised with 2 mol% CsF initiator, displaying a glass transition at 160 °C

<u>**Characterisation information of ARKEMA Kepstan 6002 for comparative purposes**</u>

NMR (^{1}H, ^{13}C, HMBC) spectra

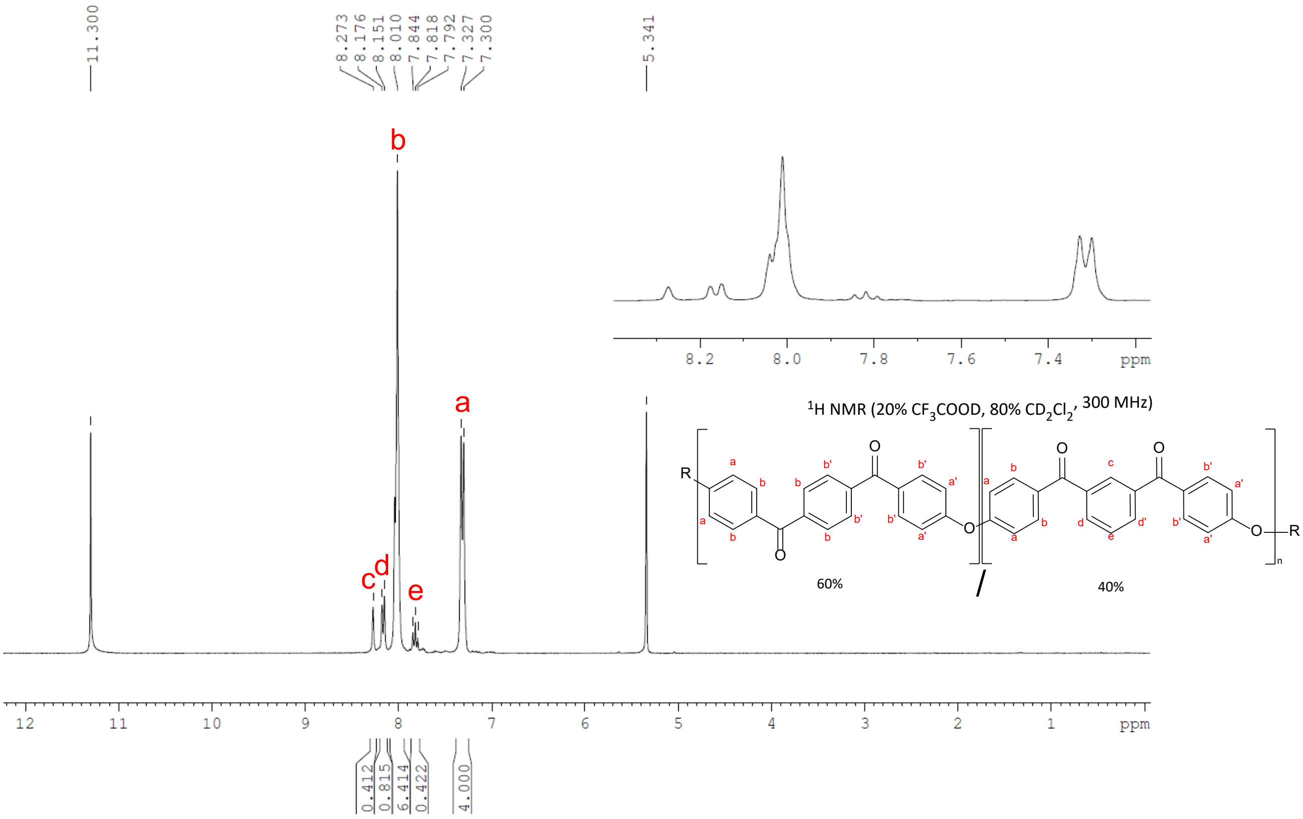

Figure S45: ^{1}H NMR (20% CF_3COOD, 80% CD_2Cl_2, 300MHz) of ARKEMA Kepstan 6002 with a 60/40 T/I ratio

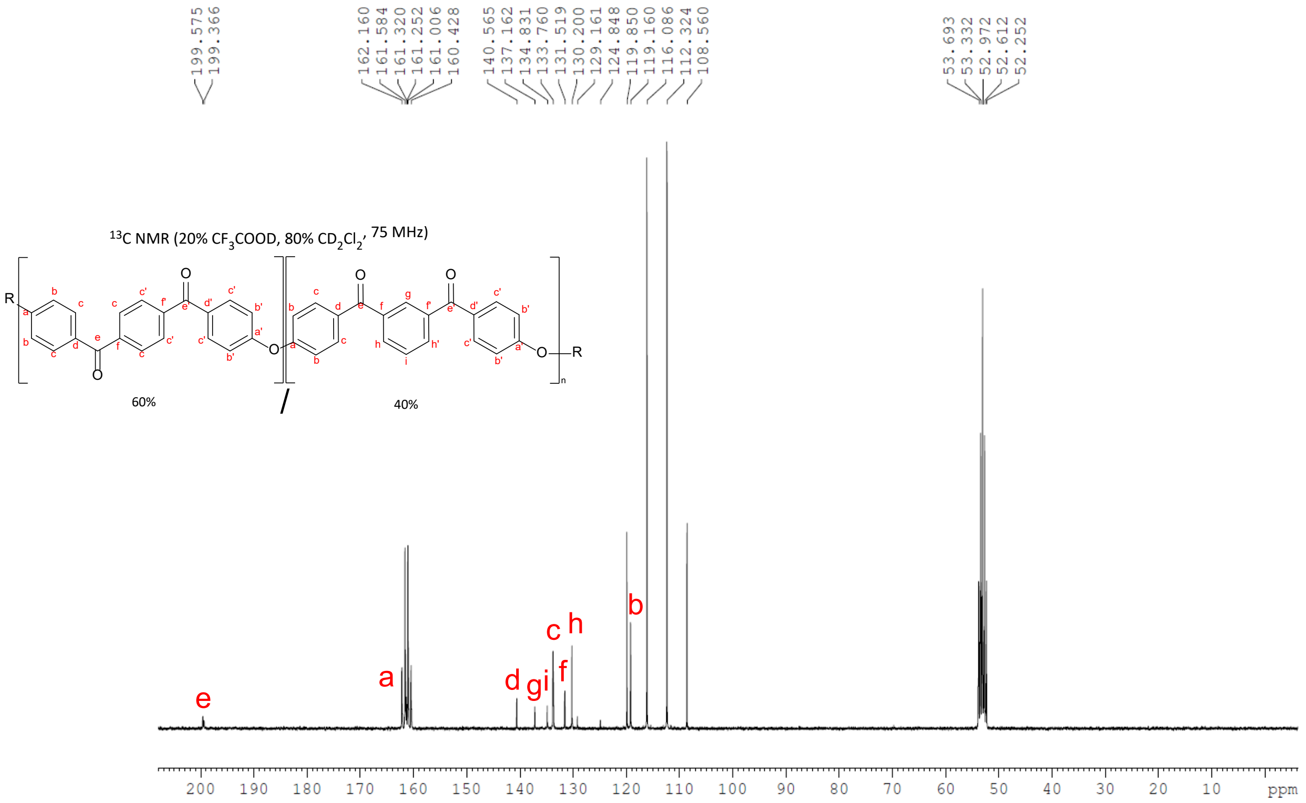

Figure S46: ^{13}C NMR (20% CF_3COOD, 80% CD_2Cl_2, 75 MHz) of ARKEMA Kepstan 6002 with a 60/40 T/I ratio

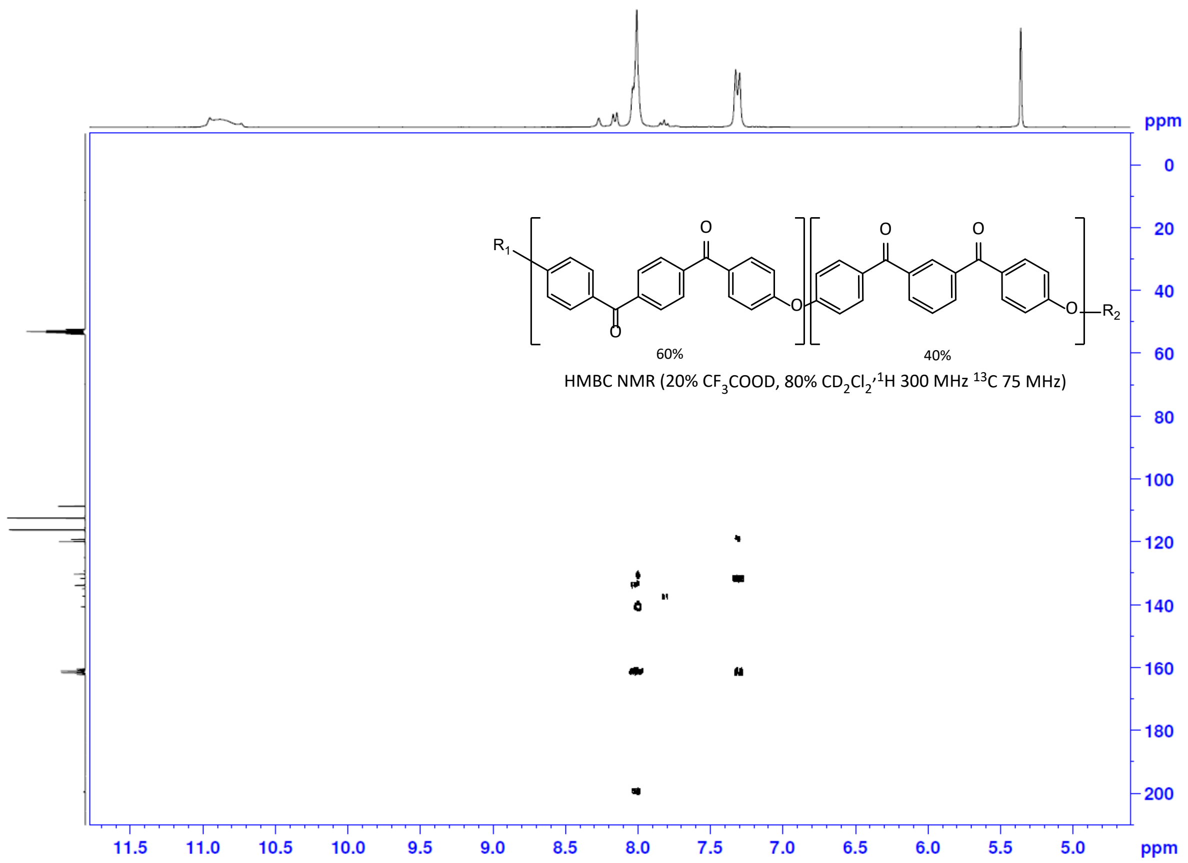

Figure S47: HMBC NMR (20% CF3COOD, 80% CD2Cl2, ¹H 300 MHz ¹³C 75 MHz) of ARKEMA Kepstan 6002 with a 60/40 T/I ratio

Characterisation information for the thioketal derivative of the polymer produced from m-PEKK MCOs

NMR (^{1}H, ^{13}C) spectra

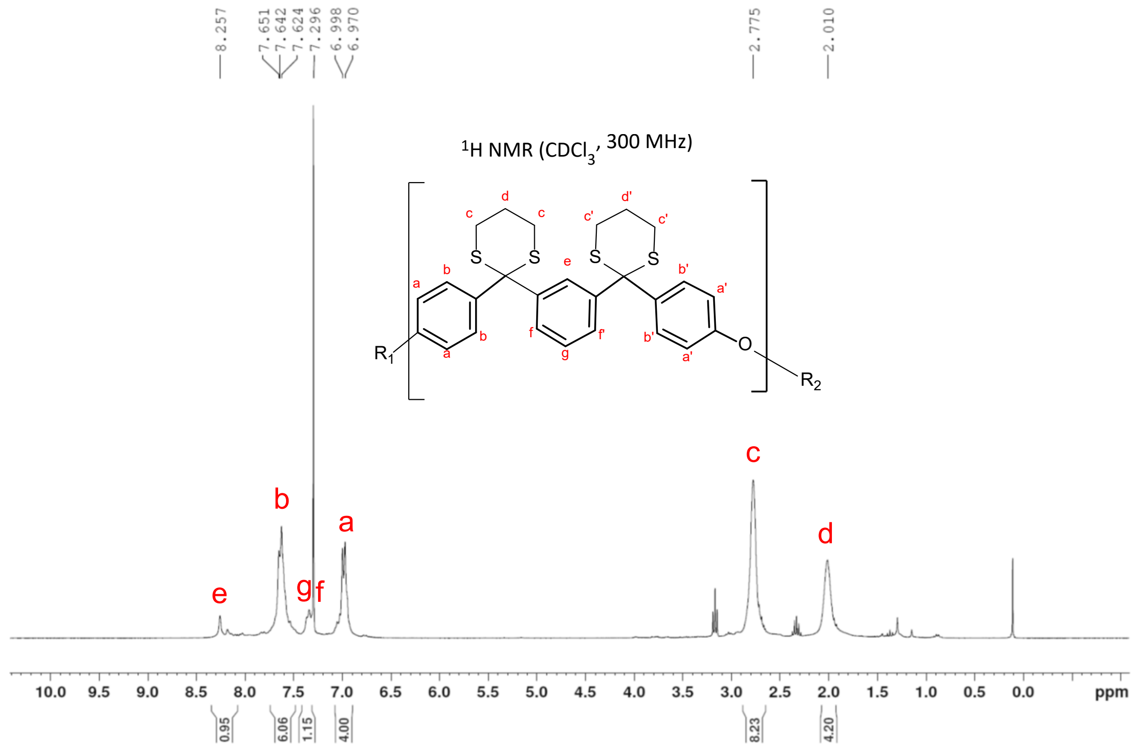

Figure S48: ^{1}H NMR (CDCl3, 300 MHz) of thioketal derivatised m-PEKK polymer formed from MCOs, with some residual 1,3 propanedithiol present as an impurity

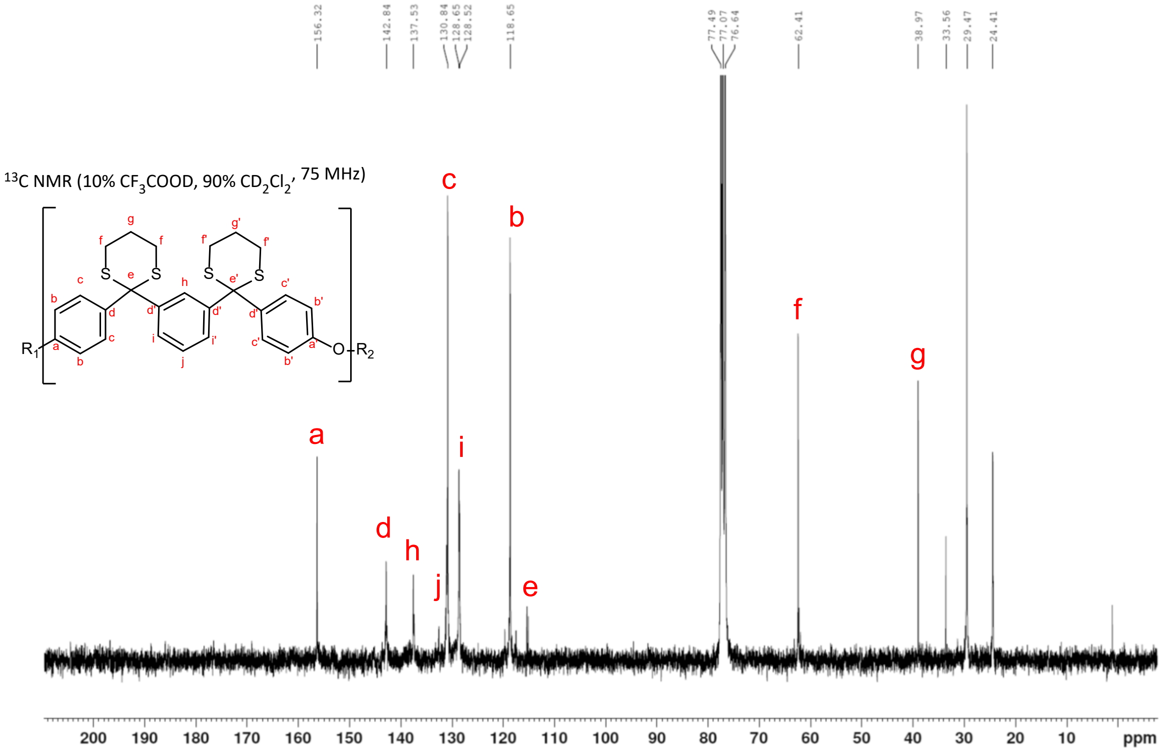

Figure S49: ^{13}C NMR (CDCl3, 75 MHz) of thioketal derivatised m-PEKK polymer formed from MCOs, with some residual 1,3 propanedithiol present as an impurity

GPC (uncorrected for thioketal derivatisation)

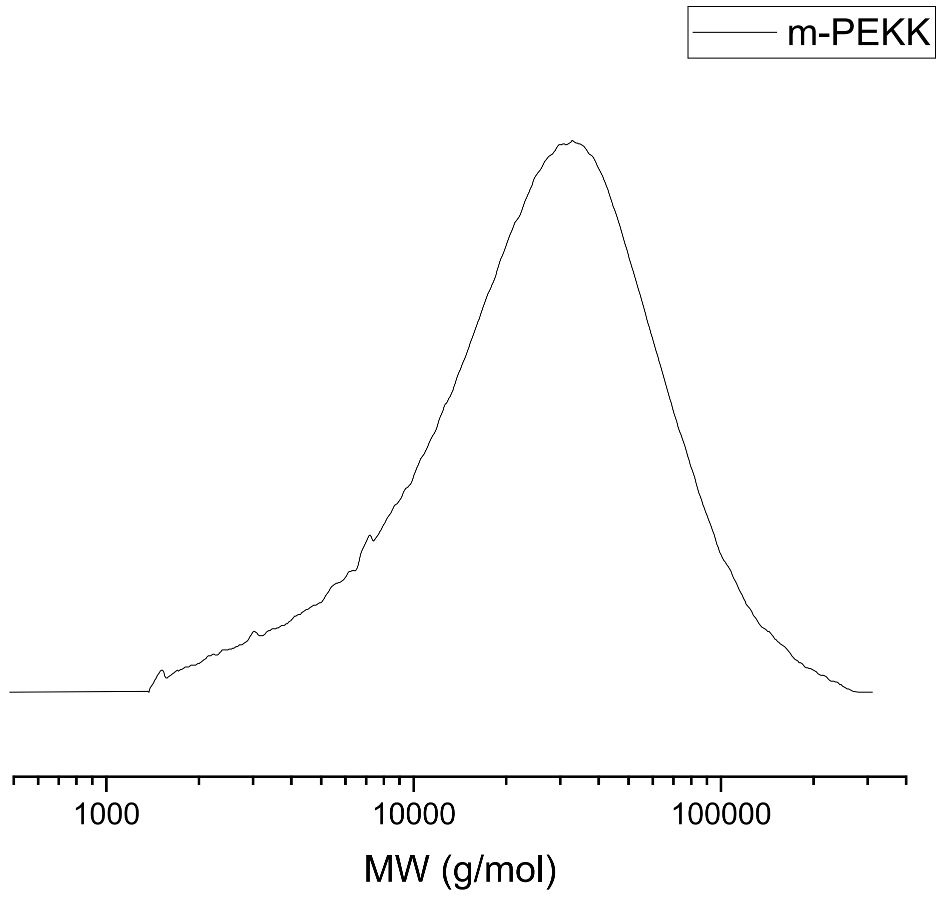

Figure S50: GPC chromatogram of the thioketal derivatised m-PEKK polymer produced from MCOs (uncorrected for derivatisation)

Characterisation information for the thioketal derivative of ARKEMA Kepstan 6002 for comparative purposes

NMR (^1H, ^{13}C) spectra

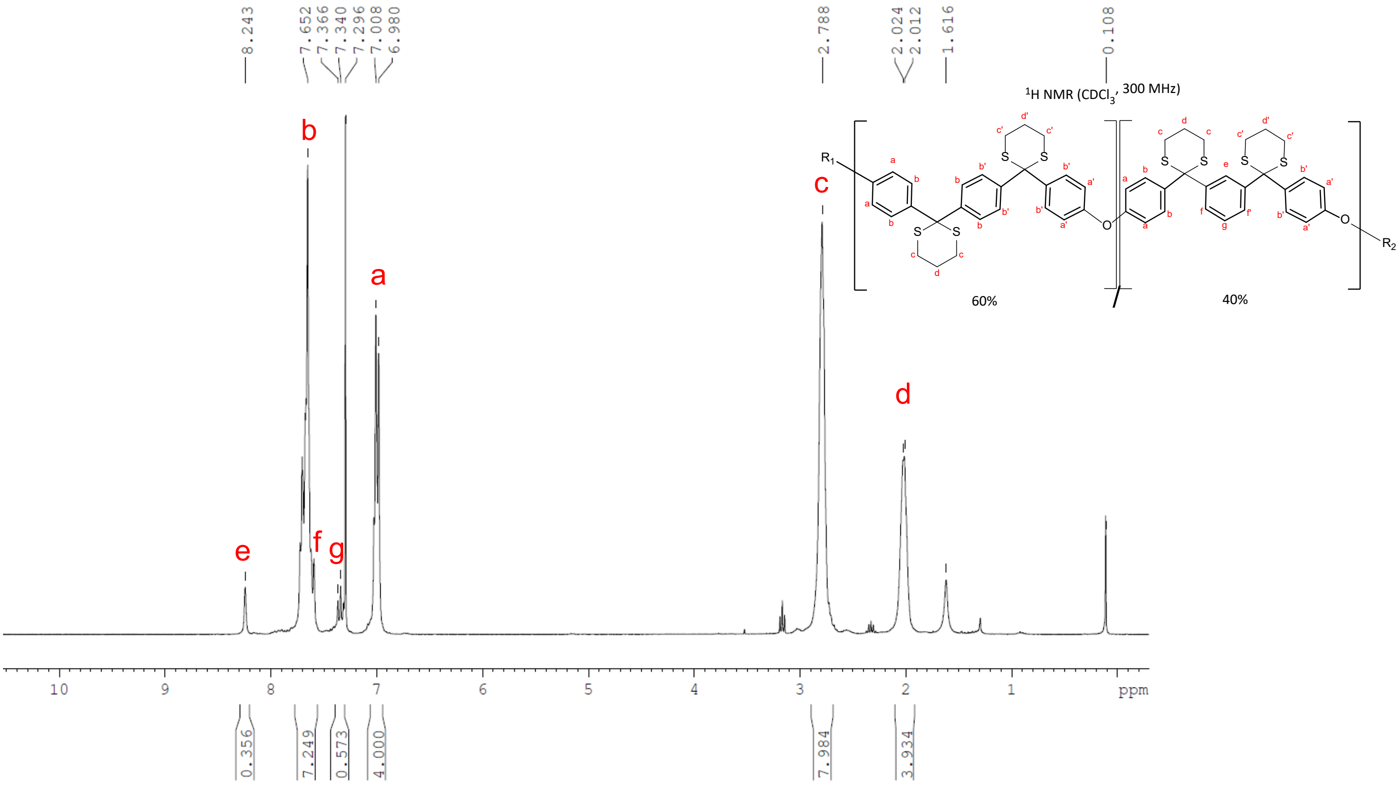

Figure S51: ^1H NMR ($CDCl_3$, 300 MHz) of thioketal derivatised ARKEMA Kepstan 6002 with a 60/40 T/I ratio

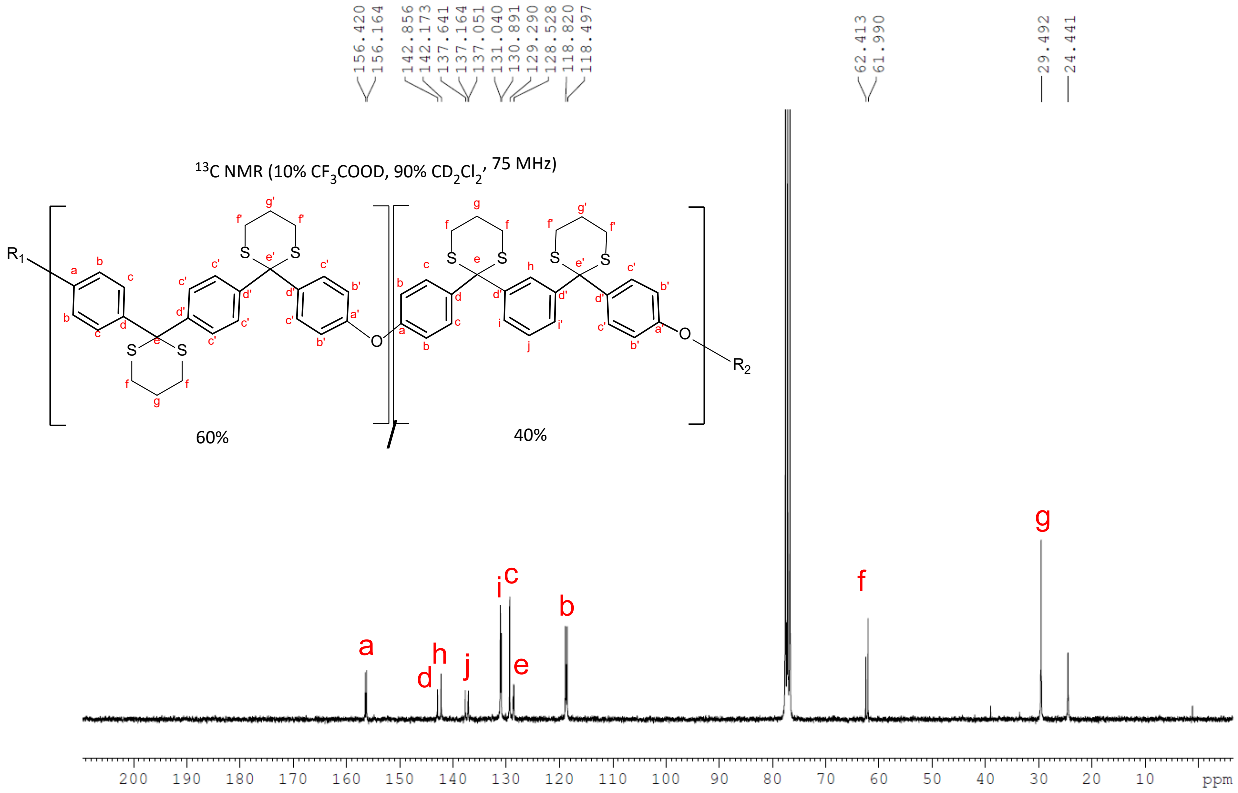

Figure S52: ^{13}C NMR ($CDCl_3$, 75 MHz) of thioketal derivatised ARKEMA Kepstan 6002 with a 60/40 T/I ratio

GPC (uncorrected for thioketal derivatisation)

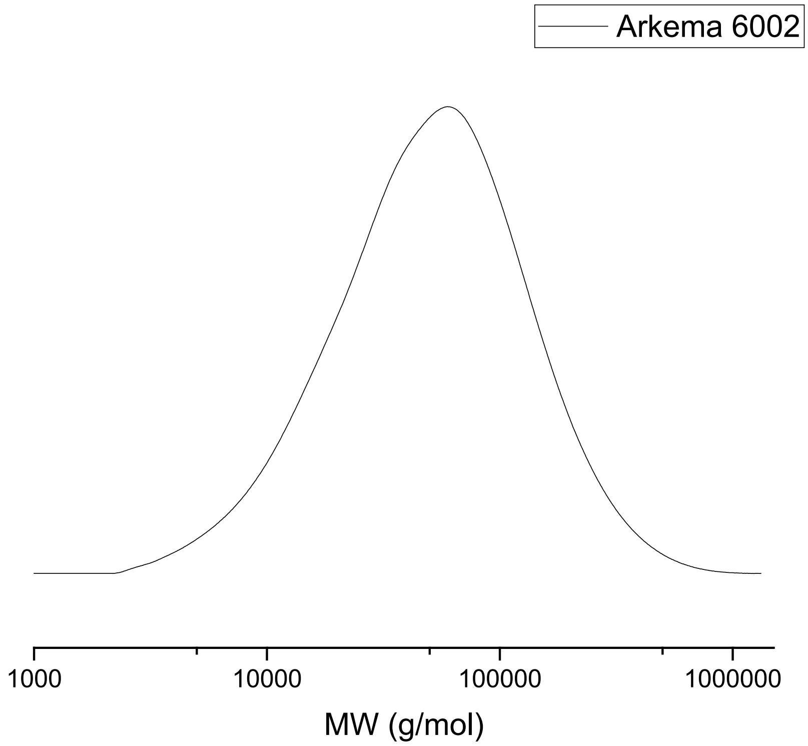

Figure S53: GPC chromatogram of thioketal derivatised ARKEMA Kepstan 6002 (uncorrected for derivatisation)
